# The Role of *cis* Regulatory Evolution in Maize Domestication

**DOI:** 10.1371/journal.pgen.1004745

**Published:** 2014-11-06

**Authors:** Zachary H. Lemmon, Robert Bukowski, Qi Sun, John F. Doebley

**Affiliations:** 1 Laboratory of Genetics, University of Wisconsin – Madison, Madison, Wisconsin, United States of America; 2 Bioinformatics Facility, Institute of Biotechnology, Cornell University, Ithaca, New York, United States of America; Stanford University, United States of America

## Abstract

Gene expression differences between divergent lineages caused by modification of *cis* regulatory elements are thought to be important in evolution. We assayed genome-wide *cis* and *trans* regulatory differences between maize and its wild progenitor, teosinte, using deep RNA sequencing in F_1_ hybrid and parent inbred lines for three tissue types (ear, leaf and stem). Pervasive regulatory variation was observed with approximately 70% of ∼17,000 genes showing evidence of regulatory divergence between maize and teosinte. However, many fewer genes (1,079 genes) show consistent *cis* differences with all sampled maize and teosinte lines. For ∼70% of these 1,079 genes, the *cis* differences are specific to a single tissue. The number of genes with *cis* regulatory differences is greatest for ear tissue, which underwent a drastic transformation in form during domestication. As expected from the domestication bottleneck, maize possesses less *cis* regulatory variation than teosinte with this deficit greatest for genes showing maize-teosinte *cis* regulatory divergence, suggesting selection on *cis* regulatory differences during domestication. Consistent with selection on *cis* regulatory elements, genes with *cis* effects correlated strongly with genes under positive selection during maize domestication and improvement, while genes with *trans* regulatory effects did not. We observed a directional bias such that genes with *cis* differences showed higher expression of the maize allele more often than the teosinte allele, suggesting domestication favored up-regulation of gene expression. Finally, this work documents the *cis* and *trans* regulatory changes between maize and teosinte in over 17,000 genes for three tissues.

## Author Summary

Modification of *cis* regulatory elements to produce differences in gene expression level, localization, and timing is an important mechanism by which organisms evolve divergent adaptations. To examine gene regulatory change during the domestication of maize from its wild progenitor, teosinte, we assessed allele-specific expression in a collection of maize and teosinte inbreds and their F_1_ hybrids using three tissues from different developmental stages. Our use of F_1_ hybrids represents the first study in a domesticated crop and wild progenitor that dissects *cis* and *trans* regulatory effects to examine characteristics of genes under various *cis* and *trans* regulatory regimes. We find evidence for consistent *cis* regulatory divergence that differentiates maize from teosinte in approximately 4% of genes. These genes are significantly correlated with genes under selection during domestication and crop improvement, suggesting an important role for *cis* regulatory elements in maize evolution. This work provides valuable insight into the evolution of gene regulatory elements during the domestication of an important crop plant.

## Introduction

Changes in the *cis* regulatory elements (CREs) of genes with functionally conserved proteins have been considered a key mechanism, if not the primary mechanism, by which the diverse forms of multicellular eukaryotic organisms evolved [1]–[3]. Variation in CREs allows for the deployment of tissue specific patterning of gene expression, differences in developmental timing of expression, and variation in the quantitative levels of gene expression. Furthermore, modification of CREs, as opposed to coding sequence changes, are assumed to have less pleiotropy and consequently have a lower risk of unintended deleterious effects in secondary tissues. The importance of CREs for the development of novel morphologies is supported by the growing catalog of examples for which differences in gene specific CREs between closely related species contributed to the evolution of diversity in form [4].

While compelling evidence for the importance of CREs in evolution has come from mapping causative variants to CREs, additional evidence has emerged from genomic analyses that show abundant *cis* regulatory variation both within [5]–[8] and between species [9]–[11]. Some studies have reported a bias such that genes with *cis* differences between species or ecotypes show preferential up-regulation of the alleles of one parent, possibly as a result of natural selection [7], [11], [12]. Consistent with the hypothesis that *cis* differences are a key element of adaptive evolution, divergence for *cis* regulation between yeast species is more often associated with positive selection than *trans* divergence [10], [13].

Crop plants offer a powerful system for the investigation of evolutionary mechanisms because they display considerable divergence in form from their wild progenitors, yet exhibit complete cross-fertility with these progenitors [14]–[16]. QTL fine-mapping experiments have provided multiple examples of modified CREs that underlie trait divergence between crops and their ancestors. These studies include examples in which *cis* changes confer the up-regulation of a gene during domestication [17], the down-regulation of a gene [18], [19], the loss of a tissue specific expression pattern [20], the gain of a tissue specific expression pattern [21], and a heterochronic shift in the expression profile [22]. These diverse modifications suggest that changes in CREs offer a powerful means to fine-tune gene expression to generate new plant morphologies.

Several transcriptional profiles contrasting crops and their ancestors have been performed, although the experimental designs used did not allow separation of *cis* and *trans* effects. These studies have shown that hundreds or even thousands of genes have altered expression in crops as compared to their progenitors and that genes with altered expression often show evidence for selection [23]–[25]. The data suggest massive alterations in gene expression profiles accompanied domestication. Work in cotton and maize shows a more frequent up-regulation of genes in the crop as compared to the wild ancestor, however whether this was due to *cis* or *trans* effects was not discernible [23], [24].

In this study, we used RNAseq to parse genome-wide expression differences between maize and its progenitor, teosinte (*Zea mays* ssp. *parviglumis*), into *cis* and *trans* effects. Three tissue types were assayed: immature ear, seedling leaf, and seedling stem. Approximately 70% of the 17,000 genes assayed show evidence of regulatory divergence between maize and teosinte. Over 1,000 genes show *cis* divergence that is highly consistent across our sampled maize and teosinte lines. For ∼70% of genes with consistent *cis* effects, the *cis* effects are specific to a single tissue type. The number of genes with *cis* differences is greatest for the ear, which underwent a profound transformation in form during domestication. Genes with *cis* regulatory differences between maize and teosinte are correlated with genes that show evidence for positive selection during domestication while *trans* genes are not. Maize also possesses less *cis* regulatory variation than teosinte over all genes and this deficit in maize is greatest for genes with *cis* regulatory divergence from teosinte. We observed a directional bias in genes with *cis* differences such that maize alleles were more frequently up-regulated than teosinte. Finally, our data provide a catalog of *cis* and *trans* regulatory variation for over 17,000 genes in three tissue types for 15 maize and teosinte inbred lines.

## Results

### RNAseq provides expression data for more than 17,000 genes in three tissues

RNAseq data for seedling leaf, seedling stem (including the shoot apical meristem), and immature ear from six maize inbreds, nine teosinte inbreds, and 29 of their F_1_ hybrids were used to examine variation in gene expression on a genome-wide scale. In total, 259 RNAseq libraries were constructed from an average of 1.96 biological replicates for each parent inbred and F_1_.

Overall, 996 million, 1.13 billion, and 1.21 billion F_1_ hybrid and 286 million, 283 million, and 276 million parent RNAseq reads were collected for ear, leaf, and stem tissue types, respectively (Table 1). These reads were aligned to custom-made parent specific pseudo-transcriptomes containing an average of 54,000 segregating sites (SNPs or small indels) in each of the 29 maize-teosinte contrasts. For F_1_ hybrid reads, 556 million, 672 million, and 716 million reads mapped to pseudo-transcriptomes in ear, leaf, and stem tissue, respectively. In parent inbred lines, 171 million, 170 million, and 163 million reads mapped to the pseudo-transcriptomes (Table 1). Thus, approximately the same percentage of reads (58.2% and 59.6%) mapped to pseudo-transcriptomes in both the F_1_ hybrids and parent datasets with about 7.15% of the total reads mapping to segregating sites in the individual F_1_ hybrids and their parents.

**Table 1:** Assignable RNAseq read counts from F_1_ hybrids and parents.

		F_1_ Hybrid Count	Parent Count	F_1_ Hybrid Percent of Total	Parent Percent of Total
Total Reads	Ear	996,210,711	286,233,926	-	-
	Leaf	1,133,517,167	282,553,096	-	-
	Stem	1,211,779,746	276,295,164	-	-
Aligned Reads	Ear	556,387,109	171,185,368	55.85%	59.81%
	Leaf	671,815,900	169,564,817	59.27%	60.01%
	Stem	716,223,906	162,866,225	59.11%	58.95%
Segregating Site Reads	Ear	74,556,872	85,296,872^a^	7.48%	29.80%^a^
	Leaf	73,094,422	78,878,805^a^	6.45%	27.92%^a^
	Stem	91,355,219	78,583,423^a^	7.54%	28.44%^a^

a A higher number and percentage of reads map to segregating sites in parents due to each set of parent reads being used in multiple comparisons. In contrast each of the F_1_ comparisons can only map to segregating sites between two pseudo-transcriptomes.

**Figure 1:**
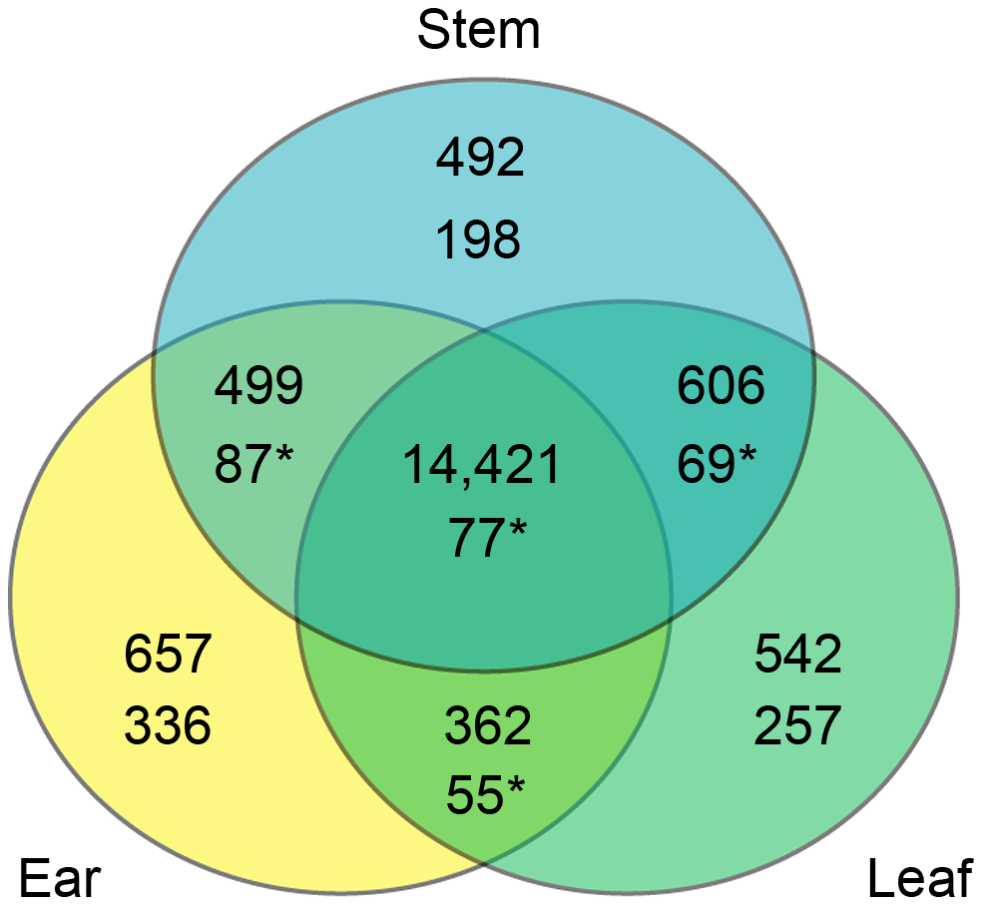
Overlap of genes assessed in the three tissues overall and in the CCT-AB gene list. Each compartment of the Venn diagram contains the tissue combination on top, number of genes overall in the middle, and number of genes from the CCT-AB gene list on bottom. CCT-AB overlap numbers marked by an “*” indicate significantly more overlap than expected by chance (permutation tests, p<1e-5). In the overall analysis the vast majority of genes (82%) were assayed in all three tissues. While this percent is much smaller for the CCT-AB candidate gene list (∼7%), this is still more of an overlap than expected by chance. The much higher degree of overlap of CCT-AB genes than expected suggests some CREs act in multiple tissues. Additionally, there are also many single tissue CCT-AB genes, which points towards the many *cis* elements that appear to function in tissue specific patterns.

RNAseq reads for all 29 F_1_ hybrids and 15 parents that aligned to segregating sites in the transcriptomes represent 23,816, 24,055, and 24,643 genes for ear, leaf and stem tissues, respectively (Table 2). The union of these three groups is 25,619 genes, which is 65% of the 39,423 genes from the maize filtered gene set (version 5b). We applied a filter to this list, requiring a read depth of 100 in both the parent inbreds and F_1_ hybrids. This filter reduced the lists to 15,939, 15,931, and 16,018 genes in ear, leaf, and stem tissues, respectively. The union of these three groups is 17,579 genes or ∼45% of the filtered gene set. There is a large degree of overlap among the genes expressed in the three tissues with 14,421 of 17,579 genes (82%) seen in all three tissues. Of the remaining genes, 1,467 are in two tissues and 1,691 are in only a single tissue (Figure 1).

**Table 2:** Number of total, CCT (*cis* only or *cis*+*trans*), *trans* only, and *cis* only genes for which RNAseq data was collected and expression was assayed.^1^

	Ear	Leaf	Stem	Union
Genes with mapped RNAseq reads	32,858	32,645	33,316	34,636
Genes with reads and segregating sites	23,816	24,055	24,643	25,619
Genes with 100 or more total depth	15,939	15,931	16,018	17,579
Total CCT genes	5,618	5,398	5,435	10,095
Filtered CCT genes^2^	4,770	4,494	4,601	8,396
ABC-List CCT	1,545	1,327	1,371	3,027
C-List CCT	990	869	940	2,326
B-List CCT	512	436	404	1,042
A-List CCT	43	22	27	69
ABC-List *trans* only	1143	1273	534	2665
C-List *trans only*	666	790	385	1721
B-List *trans only*	419	455	144	984
A-List *trans only*	58	28	5	90
ABC-List *cis* only	859	594	832	1866
C-List *cis* only	561	412	582	1380
B-List *cis* only	272	172	234	590
A-List *cis* only	26	10	16	41

1 Only genes from the maize filtered gene set (version 5b) were considered.

2 Filtered to include genes assayed in 15 F_1_ hybrids including three unique maize and five unique teosinte inbred lines.

### Prolific regulatory variation but few consistent *cis* differences

**Figure 2:**
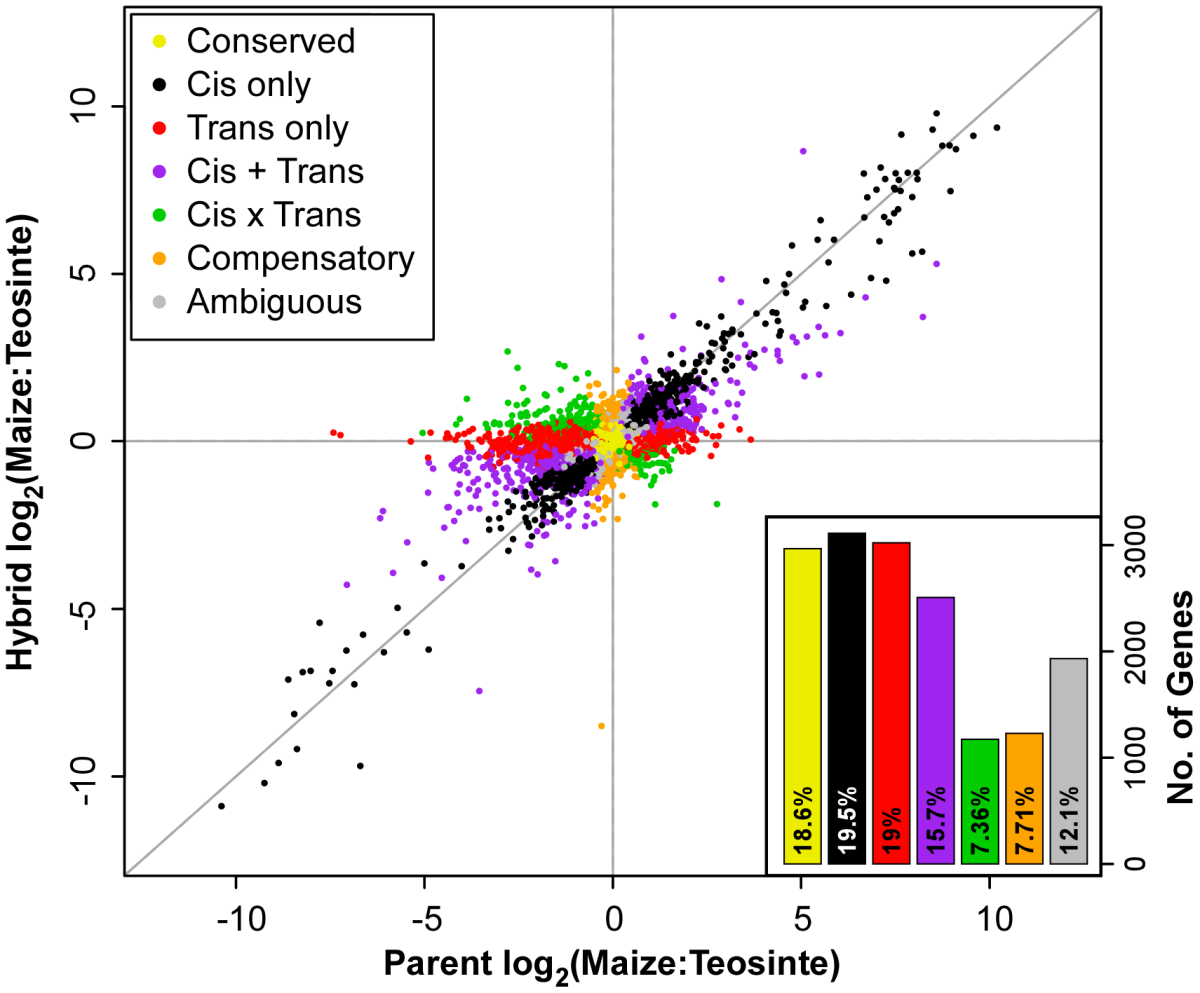
Log_2_ of parent versus hybrid ear tissue allele-specific expression ratios. The parent (x-axis) versus F_1_ hybrid (y-axis) allele-specific expression ratios for 15,939 genes are plotted against each other. Regulatory category in terms of the combination of significant statistical tests determined using the method described in methods is shown designated by color. Proportion and count of genes falling into the various regulatory categories are also shown in the lower right hand corner barplot.

We measured *cis* regulatory effects as log_2_ of the ratio of maize to teosinte read counts in F_1_ hybrids, and the *trans* effects as the difference between the F_1_ and parent log_2_ ratios. Binomial and Fisher's exact tests were used to determine whether these ratios deviated from 1∶1 and to assign genes to one of seven regulatory categories (Table 3; see Materials and Methods). In overall maize versus teosinte comparisons, about 70% of genes (69.27% ear, 74.21% leaf, and 63.82% stem genes) from the three tissues were classified as having significant *cis* and/or *trans* regulatory effects (Figure 2, Figure S1). The remaining genes were classified as having conserved (18.6%, 15.5%, and 20.7%) or ambiguous (12.1%, 10.2%, and 15.5%) expression patterns. All three tissues had similar proportions of genes falling into the different regulatory categories in the overall maize-teosinte comparison (Figure 2, Figure S1).

**Table 3:** Regulatory category as defined by significant (Sig.) with FDR<0.005 or not significant (Not Sig.) binomial tests (BT) and Fisher's exact tests (FET).

Category	Parent BT	Hybrid BT	FET	Favored allele?
*Cis*	Sig.	Sig.	Not Sig	-
*Trans*	Sig.	Not Sig	Sig.	-
*Cis*+*Trans*	Sig.	Sig.	Sig.	Same
*Cis*×*Trans*	Sig.	Sig.	Sig.	Opposite
Compensatory	Not Sig	Sig.	Sig.	-
Conserved	Not Sig	Not Sig	-	-
Ambiguous	All other patterns of significance or non-significance			

**Figure 3:**
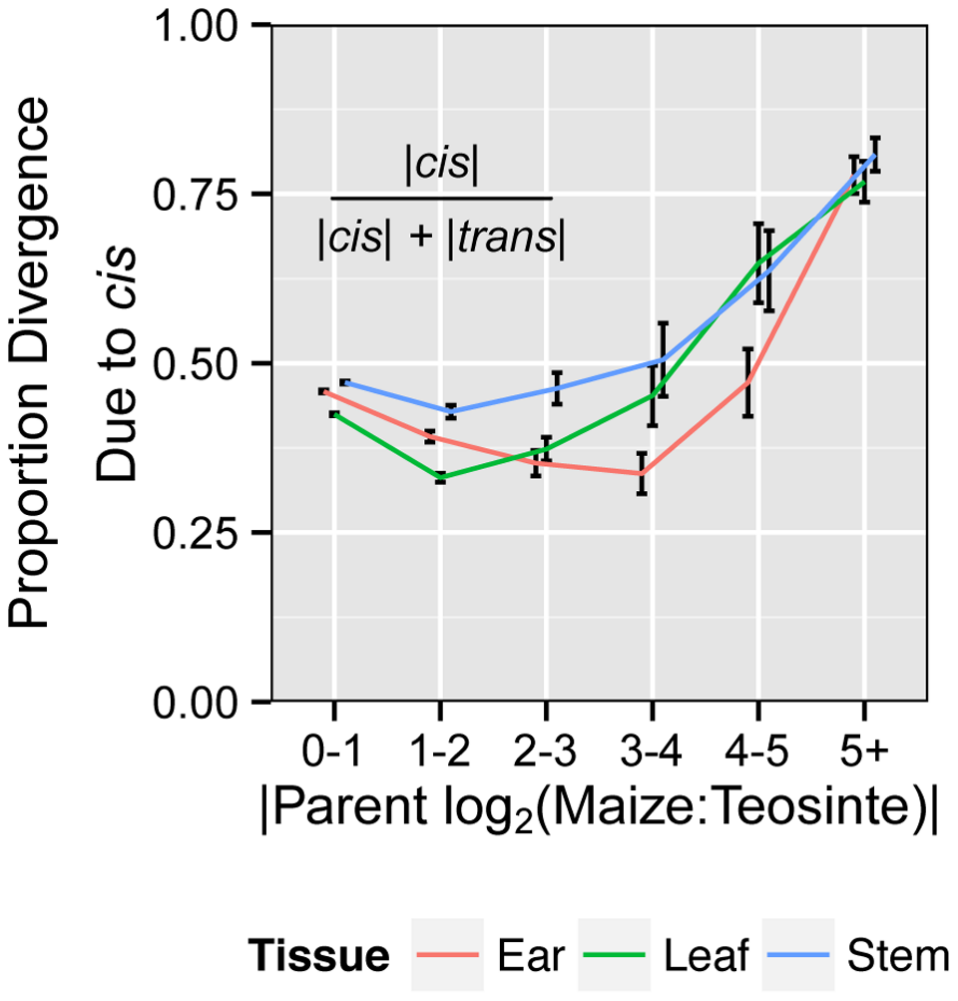
Proportion of expression divergence due to *cis* regulatory differences. The amount of total differential expression between the maize and teosinte parents due to the directly measured *cis* effect (F_1_ hybrid expression ratio) is shown with error bars depicting 95% confidence intervals. Total divergence (log_2_ of parent expression ratio) was binned from 0–1, 1–2, 2–3, 3–4, 4–5, and 5+. Divergence due to *cis* effects increases with total divergence, suggesting large expression differences tend to be caused by *cis* rather than *trans* regulatory differences.

We asked what proportion of regulatory divergence between maize and teosinte was due to *cis* effects by calculating the ratio: |*cis*|/(|*cis*|+|*trans*|) [11]. Overall, *cis* effects account for 45%, 42% and 47% of regulatory divergence for ear, leaf and stem tissue, respectively (Table S1). We further asked the relative contribution of *cis* and *trans* in generating large expression differences by binning genes based on the magnitude of overall expression difference (|log_2_ parent ratio|). This analysis shows that the proportion of divergence due to *cis* is greater with greater total divergence in expression (Figure 3). At high degrees of expression divergence between maize and teosinte (log_2_ change of 5 or more), over 75% of divergence is due to *cis*. Thus, large expression differences appear to be caused primarily through difference in *cis* regulation as opposed to *trans*.

A primary goal in this study was to identify genes with *cis* regulatory differences between maize and teosinte. Such genes in the *cis* only or *cis* plus *trans* regulatory categories (CCT genes) are candidates for direct targets of selection during maize domestication or improvement for altered gene expression. We identified 5,618 ear, 5,398 leaf and 5,435 stem CCT genes in the overall analysis (Table 2). To exclude CCT genes with little data, the list was filtered to include only genes assayed in at least 15 maize-teosinte F_1_s involving at least three maize and five teosinte inbred lines. This filtering resulted in 4,770 ear, 4,494 leaf, and 4,601 stem CCT genes (union of 8,396 genes). Next, we asked if genes on the filtered CCT list consistently favor expression of maize or teosinte alleles in the individual F_1_ hybrids. The goal was to exclude CCT genes for which the significant overall *cis* effect was caused by a large expression bias in one or a few F_1_ crosses. We defined three levels of consistency: groups A, B and C for which 100%, 90% and 80% of F_1_s showed the same directionality, respectively. Groups A, B, and C genes combined across tissues contained 69, 1,042, and 2,326 genes respectively (Table 2). Thus, relatively few of the 8,396 filtered CCT genes show a significant overall *cis* effect that is highly consistent among 15 or more F_1_ hybrids.

### Possible directional bias in *cis* evolution

**Figure 4:**
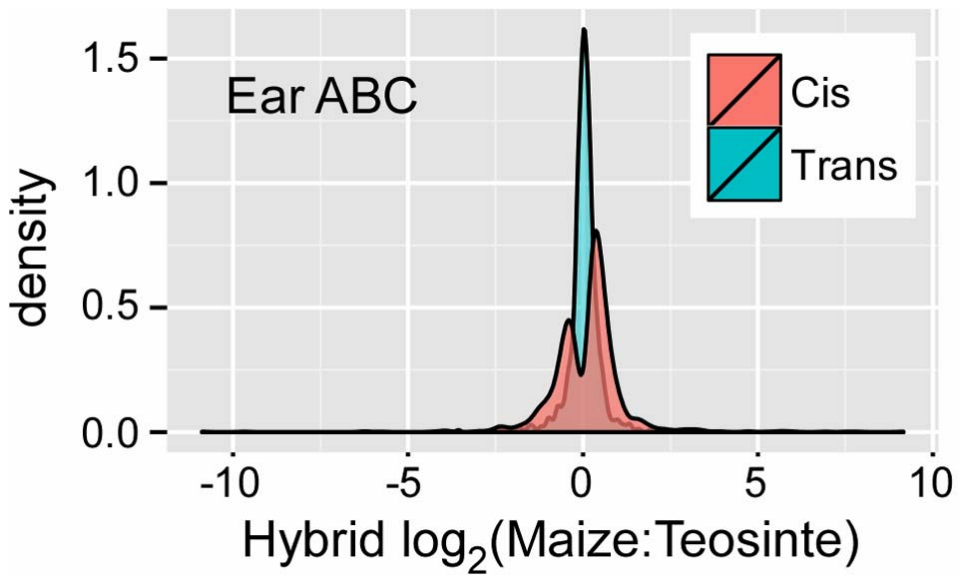
*Cis* versus estimated *trans* regulatory effect for CCT-ABC genes in the ear. CCT genes have a directional bias with more genes overall favoring the maize allele (positive ratio) than teosinte (negative ratio). Genes with consistent *cis* regulatory differences tend to favor the domesticated maize allele.

Visual examination of Figure 1 shows a greater density of *cis* genes (black points) with positive log_2_ hybrid expression ratios than with negative ratios, suggesting *cis* evolution during domestication more often favored alleles with increased expression in maize relative to teosinte. Consistent with this visual observation, the number of CCT (ABC list) genes with a positive (maize biased) versus negative (teosinte biased) log_2_ hybrid expression ratio are 947∶598, 844∶483 and 826∶545 for ear, leaf and stem, respectively (Table S2). All of these ratios are significantly different from a 50∶50 unbiased expectation (binomial test, p<0.001). Additionally, a plot of the distribution of log_2_ hybrid expression ratio for CCT genes shows a greater density of genes with positive values for all three tissues (Figure 4, Figure S2).

The apparent bias in directionality of *cis* evolution could be the result of error in our bioinformatics pipeline. One potential error is preferential alignment of maize RNAseq reads due to greater sequence divergence of teosinte lines from the reference transcriptome (B73) as compared to maize inbred lines. If such systematic error exists, the observed bias in directionality of *cis* evolution would be expected to be greatest for F_1_s involving the reference B73 (zero alignment bias of maize reads and high bias for teosinte) and less extreme for crosses between teosinte and non-reference maize lines (moderate bias for non-reference maize and high bias for teosinte).

To test this expectation, we calculated the number of CCT (ABC list) genes with positive versus negative log_2_ hybrid expression ratios for F_1_s involving B73 and non-B73 maize parents separately. For ear tissue, there are 569 teosinte-biased and 975 maize-biased genes for B73 F_1_s and 606 teosinte-biased and 939 maize-biased genes for non-B73 F_1_s. A Fisher's exact test fails to reject the null hypothesis of equivalent ratios (p = 0.18). There was also no evidence for non-equivalent ratios with the other two tissue types (Table S3). Thus, we see no evidence for significantly greater bias for maize alleles in crosses involving B73 versus the non-reference maize parents, suggesting alignment bias does not explain the excess of CCT genes with the maize allele expressed higher than the teosinte allele.

### Gene expression variation is greater in teosinte

**Figure 5:**
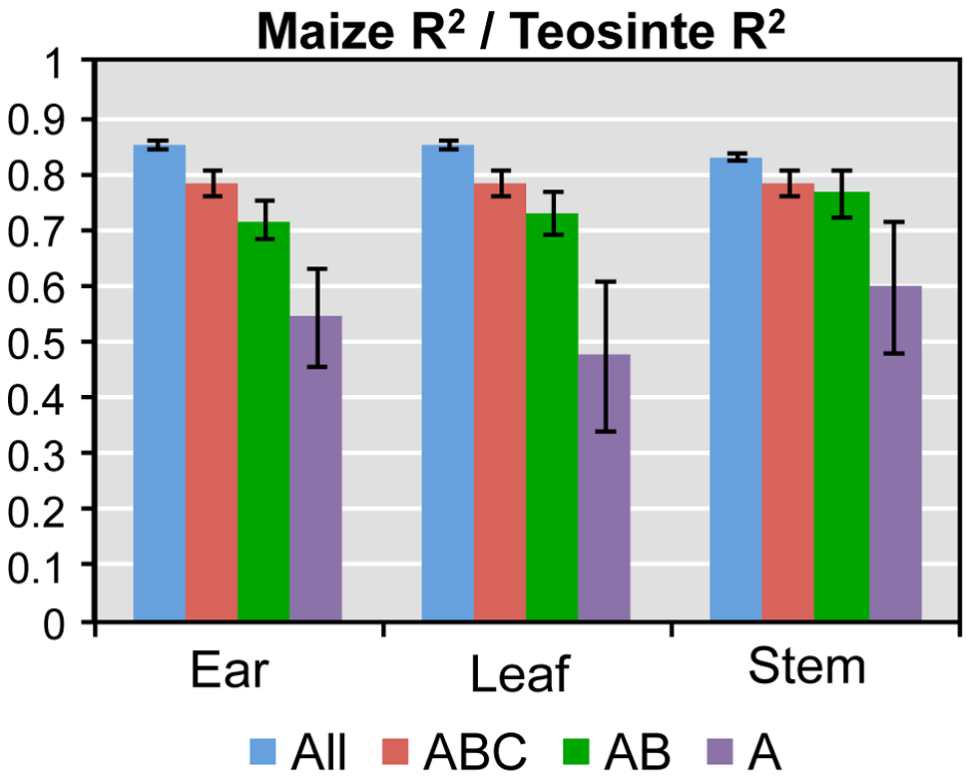
Ratio of the average maize to teosinte R^2^ values for individual genes from models explaining hybrid expression by maize and teosinte parent. In all three tissues, the proportion of maize to teosinte R^2^ decreases in candidate CCT gene lists with the strongest candidates (CCT-A) having the most extreme reduction in variation in maize relative to teosinte. Error bars indicate ± one standard error.

Both the domestication bottleneck and selection during domestication are expected to reduce variation in maize as compared to teosinte. We asked if these reductions in variation are apparent in our gene expression data. To quantify whether variation in maize or teosinte was the source of the variation in expression ratios among F_1_ hybrids, a linear model was fitted on a gene-by-gene basis with maize and teosinte inbred parent as explanatory factors for hybrid expression ratio. Among ∼13,000 genes included in this analysis, the maize parent explains only 85% as much variation as the teosinte parent (Figure 5, Table S4). The reduction is consistent across all seven regulatory categories (Figure S3). This represents the general reduction in diversity of maize as compared to teosinte, presumably a result of the domestication bottleneck.

While the bottleneck predicts reduced expression variation in maize overall, genes that were targets of selection for regulatory differences should have an even greater reduction in expression variation. Consistent with this expectation, we observed a greater reduction in variation in maize as compared to teosinte for CCT genes than the full set of ∼13,000 genes (Figure 5, Table S4). For the CCT-ABC genes, maize contributes 78% of teosinte variation, for the AB group ∼73%, and for the A group only 54% of teosinte variation. Thus, among our strongest *cis* regulatory candidate genes (A group), the data indicate that maize explains only about half as much of the *cis* regulatory variation as teosinte.

The reduction in gene expression variation in maize versus teosinte is also seen in the number of individual genes with significant effects due to the maize and/or teosinte parent on the F_1_ expression ratio (Table S5). In terms of numbers of genes on the AB list CCT genes, there were 2.0 to 2.5 fold more genes for which only the teosinte parent contributed significant variation to the F_1_ expression ratios than genes for which only the maize parent contributed. This difference is 5-fold in favor of teosinte for CCT genes on the A list.

### Selection candidate genes are enriched for CCT genes

We compared CCT genes to putative targets of selection during maize domestication and improvement from a recent study [26]. There is significant enrichment for CCT genes among selection candidate genes for all three tissues (Table 4). The strength of the association with selected genes is strongest for the union of CCT genes from all three tissues. For example, there are 139 CCT-AB genes among the selected genes, while 87.7 would be expected by chance. Also, there were 10 CCT (A-list) genes from stem tissue among selected genes when only 2.16 were expected, a nearly 5-fold enrichment.

**Table 4:** Fisher's exact tests for the overlap between domestication and improvement selection candidate genes and CCT genes from each of the three experimental tissues.

CCT Group	Overlap	Ear	Leaf	Stem	Union
A	Expected	3.42	1.41	2.16	5.63
	Observed	11	3	10	18
	p-value	3.52E-04	1.61E-01	1.89E-05	5.54E-06
AB	Expected	44.71	37.03	34.78	87.67
	Observed	70	63	60	139
	p-value	9.12E-05	1.67E-05	1.74E-05	1.27E-08
ABC	Expected	125.48	108.28	109.89	248.97
	Observed	174	140	139	318
	p-value	2.11E-06	6.33E-04	1.63E-03	2.50E-07

**Figure 6:**
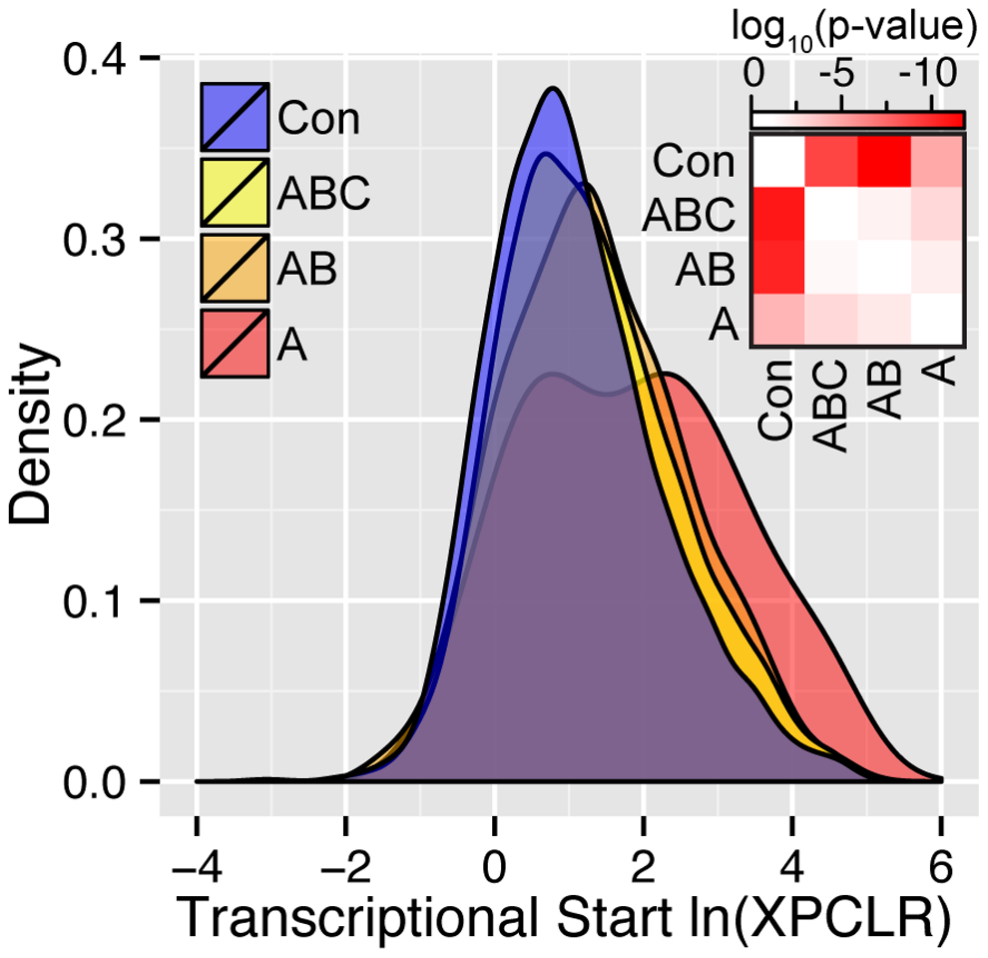
Density plots of the ln(XPCLR) score for the union of conserved versus CCT candidate genes across tissues. Union CCT (A, AB, ABC) gene lists consisting of all genes identified as CCT in any of the three tissues have a significantly higher XPCLR score in the 10 kb window holding the transcriptional start site than conserved (Con) genes. The natural log transformed XPCLR scores for CCT genes are progressively statistically higher than the union of genes identified as conserved in the initial analysis. The distributions of conserved and CCT genes are significantly different by both the shape sensitive Kolmogorov-Smirnov test (upper right of heatmap) and simple difference of the means t-test (lower right of heatmap). Heatmap scale is in log_10_(p-value).

XPCLR scores (cross population composite likelihood ratios) quantify the degree of support for positive selection on a genomic region. A comparison of the distributions of ln(XPCLR) scores at the transcriptional start site (calculated by Hufford *et al.* [26]) for the union of CCT genes (A, AB, and ABC) versus genes with conserved expression between maize and teosinte shows that CCT genes having a higher mean XPCLR than conserved genes (Figure 6). The distribution for conserved genes is significantly different than all three CCT gene lists in terms of shape (Kolmogorov-Smirnov test, p<1.59e-5) and overall mean (t-test, p<5.00e-5). This pattern was also observed for tissue specific comparisons (Figure S4).

A goal of this study was to explore the relative importance of *cis* versus *trans* regulatory divergence during maize domestication. To address this question, we examined the evidence for selection on genes with *cis* only effects in comparison to genes that have *trans* only effects. Fisher's exact tests on 2×2 contingency tables tabulating *cis* and *trans* genes with selection feature genes from Hufford *et al.* [26] show *cis* only genes are significantly enriched (p-value<0.05) for selection in 6 of 9 comparisons, while *trans* only genes are never enriched and are actually significantly underrepresented among selected genes in two cases (Table 5).

**Table 5:** Fisher's exact tests (1 tailed) for enrichment of *cis* and *trans* only genes in domestication/improvement selection features defined by Hufford *et al.* [26].

Tissue	Regulatory Category	F_1_ Comparisons Consistent	Observed	Expected	p-value
Ear	*Cis* only	A List	5	1.998	0.043
Leaf			1	0.658	0.499
Stem			3	1.316	0.138
Ear	*Trans* only	A List	4	5.327	0.818
Leaf			2	2.538	0.736
Stem			1	0.282	0.256
Ear	*Cis* only	AB List	36	24.449	0.018
Leaf			25	14.381	0.004
Stem			32	19.647	0.006
Ear	*Trans* only	AB List	28	41.954	0.020
Leaf			31	41.263	0.968
Stem			16	12.032	0.222
Ear	*Cis* only	ABC List	95	70.113	0.002
Leaf			56	46.902	0.091
Stem			84	65.615	0.016
Ear	*Trans* only	ABC List	78	97.036	0.033
Leaf			89	104.896	0.964
Stem			42	43.148	0.935

### Microarray and RNAseq data only partially correspond

We assessed the degree of correspondence between our CCT genes and 612 differentially expressed genes identified by a recent microarray study in maize [24]. We constructed 2×2 contingency tables for differentially expressed (DE) and non-differentially expressed (NDE) genes from the two studies. A Fisher's exact test shows a highly significant degree of correspondence between the two studies for all three tissues (Table 6). Using our CCT-AB lists, ∼24 genes are identified as DE in both studies per tissue while about 7 are expected by chance. However, the absolute level of correspondence between the two studies is rather low. For example, of the 350 leaf genes identified as DE by RNAseq, only 24 (7%) were also identified by the microarray study (Table S6). Thus, while the overlap between our two studies is statistically significant, the two methodologies resulted in largely different lists of DE genes.

**Table 6:** Fisher's exact tests for the overlap between differentially expressed genes from the microarray study [24] and CCT genes from each of the three experimental tissues in our work.

CCT Group	Overlap	Ear	Leaf	Stem	Union
A	Expected	0.5563	0.2757	0.3593	1.0388
	Observed	4	2	2	7
	p-value	2.14E-03	3.00E-02	4.92E-02	7.12E-05
AB	Expected	7.5008	6.8916	6.2475	16.0098
	Observed	23	24	25	47
	p-value	1.56E-06	8.98E-08	2.91E-09	1.03E-11
ABC	Expected	21.7735	19.9658	20.5788	45.9927
	Observed	52	48	46	90
	p-value	9.58E-10	4.78E-09	1.05E-07	5.76E-12

The largely different lists of DE genes identified by microarray and RNAseq analysis could be due in part to the fact that the microarray analysis includes genes with *trans* and *cis*×*trans* differences. To assess the proportion of the 612 DE genes from the microarray study that have *trans* versus *cis* effects, we examined the RNAseq-based regulatory categories of the ∼250 DE genes (241, 262, 259; ear, leaf, and stem) for which there is both microarray and RNAseq data (Table S7). About 20% of these genes are classified as *trans* only or *cis*×*trans* by RNAseq, while 55% are classified as either *cis* only or *cis*+*trans*. The remaining 25% are classified as conserved, ambiguous or compensatory. These results suggests the very different lists of DE genes from the two technologies can only be explain in part by inclusion of genes with *trans* only effects in the list of DE genes from the microarray study. Most of the difference between the lists is likely due to differences in tissue, germplasm, environment, sampling error, or other sources of error.

### CCT genes are unrelated to differentially methylated regions

In a recent study, Eichten *et al.* [27] identified differentially methylated regions (DMRs) in maize and teosinte. We compiled a list of the nearest genes both upstream and downstream of each DMR, which gave a list of 332 genes. Of these genes, we have RNAseq data from 115, 116, and 121 for the ear, leaf, and stem tissues, respectively. Of these genes, 19, 13, and 17 genes were on the CCT-ABC gene lists (Table S8). We asked if CCT-ABC list genes are over-represented among the DMR associated genes as compared to random expectation and found that they are not (Fisher's exact test, p>0.10). Finally, DMR methylation does not correspond with the allele specific expression of CCT-ABC list genes with only ∼50% agreement between methylation status and allele expression (Table S9).

### Dominant and additive gene expression inheritance

**Figure 7:**
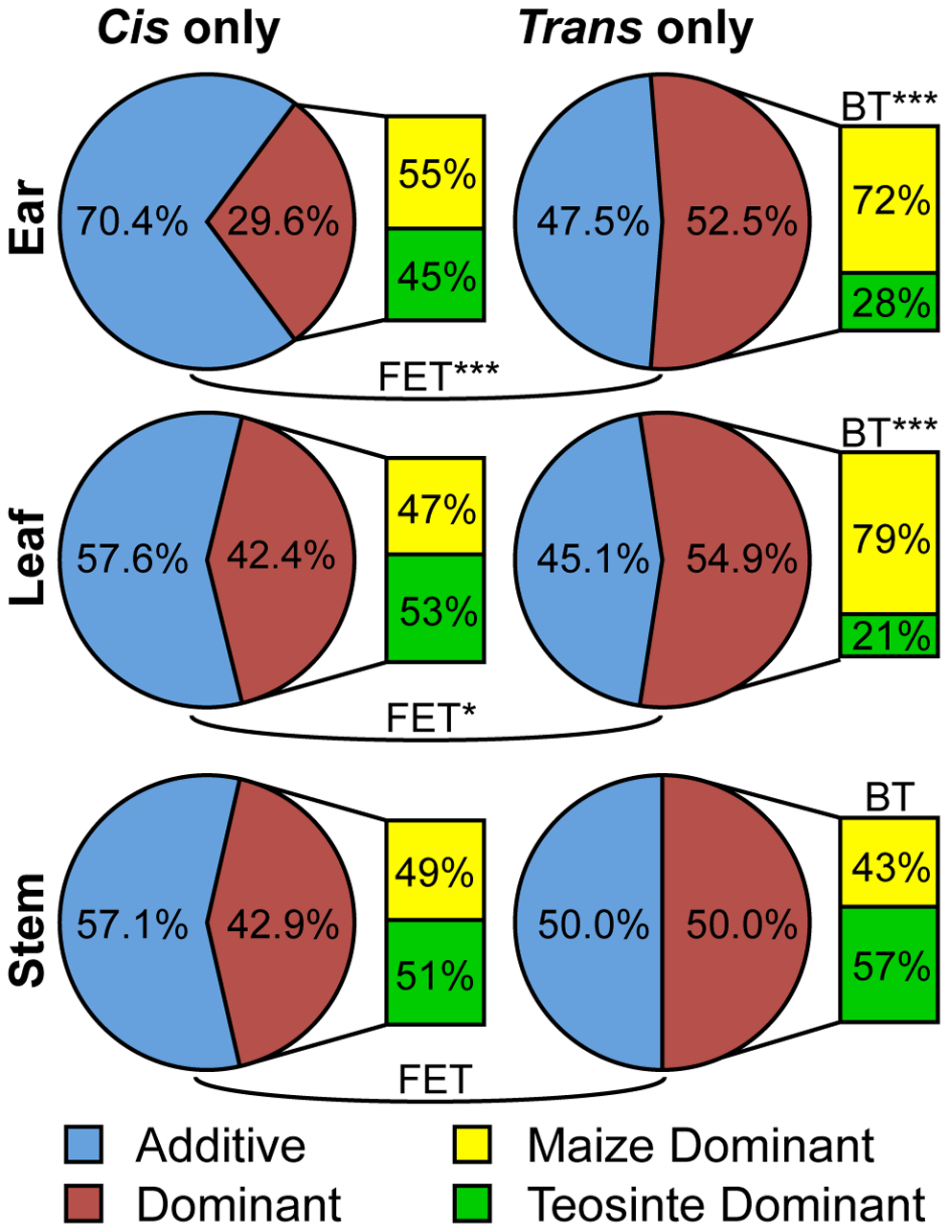
Proportion of *cis* only and *trans* only genes identified as having dominant or additive inheritance. The proportion of genes showing dominant (red) versus additive (blue) gene action for *cis* only and *trans* only AB lists. For all tissues, *trans* only genes have a higher rate of dominance, however this difference is only significant for the ear and leaf tissues (Fisher's exact test, FET). The proportion of genes in the *trans* only lists that are dominant for the teosinte allele (green) and the maize allele (yellow) is shown in the barplot to the right of each pie graph. There is significant deviation from the neutral expectation (1∶1) for the ear and leaf tissue (binomial test, BT). Fisher's exact and binomial test significance level indicated by “*” p<0.05, “**” p<0.005, and “***” p<0.0005.

We compared the proportions of genes showing dominant versus additive gene action in the *cis* only and *trans* only ABC lists. Dominant gene action of *trans* only genes will occur when there are haplo-sufficient loss of function (LOF) alleles at their *trans* regulators. In contrast, the effects of *cis* regulatory elements are expected to be purely additive in absence of transvection or similar mechanism [28]. *Cis* only genes classified as having dominant gene action may indicate error in classification due to *trans* effects below the level of statistical detection. Consistent with the expectation that dominance is more likely for *trans* only genes, the proportion of genes classified as dominant is higher for *trans* only genes in all three tissue types (Figure 7, Table S10).

It has been proposed that the allelic variants responsible for evolution during domestication are primarily recessive LOF alleles [29]. Under this model, a non-domesticated allele would be dominant to the recessive LOF domesticated allele. Among *cis* only genes with dominant gene action, dominance of the maize versus teosinte allele does not differ from the 50∶50 expectation (Figure 7, Table S10). Among *trans* only genes with dominant gene action, the maize allele is dominant to the teosinte allele more often than expected by chance. These results are counter to the proposal that domestication favored recessive LOF alleles.

### Candidate genes enrichment in various functional categories

We examined CCT-ABC genes for enrichment of several functional classes of genes including transcription factors, genes in known metabolic pathways, genes underlying QTL, and gene ontology (GO) groups. First, a list of maize transcription factors and their corresponding families were compiled from the transcription factor database [30]. Although CCT genes (AB-list) were found to be slightly enriched for several transcription factor families (ARF and MADS-MIKC) by Fisher's exact tests, these results do not stand up to Bonferroni multiple test correction (Table S11). We conclude that there is no compelling evidence that CCT genes are enriched for transcription factors.

CCT (AB list) genes were also compared with results from a recent QTL mapping experiment for a number of domestication and improvement traits [31]. We compared observed versus expected overlap between CCT genes from the three tissues to the genes located within 1.5 LOD QTL support intervals for 11 traits. Testing was done on a trait by trait basis and restricted to 1.5 LOD QTL intervals containing 20 or fewer genes. After correction for multiple testing (Bonferroni), no significant enrichment for CCT-AB genes in domestication QTL was observed (Table S12). The greatest enrichment was seen with the trait ear diameter for which there were four CCT genes assayed in ear tissue within the QTL intervals when only 1.22 were expected by chance (Fisher's exact test, p = 0.03).

A test for enrichment of CCT genes and *trans* only genes in 15 different metabolic pathways defined in the Kyoto Encyclopedia of Genes and Genomes (KEGG) was done using Fisher's exact test on 2×2 contingency tables. There was no compelling evidence for enrichment/depletion of either groups of genes in any of the 15 pathways tested (Table S13). The smallest p-value identified was for the fatty acid degradation pathway in leaf tissue for CCT ABC genes (p = 0.008), however this result does not remain significant after Bonferroni multiple test correction.

We tested for GO term enrichment and depletion in the CCT and *trans* only gene lists. These analyses found significant GO term associations in the leaf CCT-ABC gene list for depletion for DNA binding (Table S14). For *trans* only genes, significant enrichment for a number of GO terms in the ear tissue was detected for transcription factor and photosynthesis related terms with additional enrichment for ribosomal GO terms found in the leaf tissue (Table S14).

## Discussion

### Regulatory differences between maize and teosinte

Approximately 70% of the ∼17,000 assayed genes exhibit some form of regulatory difference between maize and teosinte, suggesting considerable regulatory divergence has occurred during maize domestication and improvement (Figure 2, Figure S1). Similar proportions of gene regulatory differences were observed in recent studies of *Drosophila* [11] and yeast [13] species. The high amount of expression divergence between maize and teosinte is not surprising given the incredible divergence in their morphology and the exceptional expression variation existing within maize itself. For example, a recent study found 27.9% of maize genes are only expressed in a subset of maize inbred lines with over a thousand genes absent from the reference B73 genome [32].

The high fraction of genes exhibiting regulatory divergence between maize and teosinte should be viewed with this perspective. It includes all genes with specific combinations of significant binomial and Fisher's exact tests as outlined in Table 3. Given the massive amount of data analyzed, statistically significant *cis* and *trans* effects were detected for as small as 1.02 fold expression differences, which seem unlikely to have biological significance. Moreover, many genes with significant regulatory differences between maize and teosinte “on average” show diversity in expression within maize and within teosinte such that the favored allele can change depending on the specific pair of maize-teosinte inbred lines.

### *cis* and *cis*+*trans* (CCT) candidate genes

Our primary focus was on genes with *cis* regulatory differences between maize and teosinte as this class of genes should include many direct targets of selection for domestication traits. The overall analysis classifies 8,396 genes (47.8%) as CCT genes that show *cis* divergence between maize and teosinte. This is a remarkably high proportion that reflects the considerable statistical power to detect small differences, likely with minimal biological importance, and does not consider whether the expression difference is consistent across maize-teosinte F_1_ comparisons. Thus, we focused our analysis on CCT A, B, and C gene lists showing consistent directionality of expression bias in 100%, 90% and 80% (respectively) of maize-teosinte comparisons. This approach narrows the list to 1,079 genes (A and B lists) that we consider our most robust candidates for genes with *cis* regulatory differences between maize and teosinte.

### Ear tissue possesses the most genes with *cis* regulatory differences

CCT candidate genes from the three tissues were largely different. Among the 1,079 CCT genes on A and B lists, ∼73% were identified in only a single tissue. This includes 336 ear, 257 leaf, and 198 stem specific genes. In contrast, only 77 of the 1,079 CCT genes were classified as CCT candidates in all three tissues. These results are consistent with previous studies that examined *cis* regulatory divergence between taxa in multiple tissues [8], [12]. These results highlight the importance of assaying multiple tissues and developmental stages. It also exposes a major weakness of genomic scale expression assays such as ours. Given the complex ways in which gene expression is regulated across different tissue types, genomic-scale assays in one or even multiple tissues are a very blunt instrument for exploring the evolution of gene expression.

While CCT genes are mostly tissue-specific, genes overall are not. Of the 17,579 genes assayed with at least 100 read depth in both the parents and F_1_ hybrids, 14,421 were expressed in all three tissues. This high proportion of genes (82%) expressed in all tissues creates a false impression that sampling one tissue type at one point in developmental time provides a reasonable assay of all tissues at all developmental times. The discordant observations that CCT genes are mostly tissue specific, while genes overall are expressed in all tissues can be explained by tissue specific enhancers or repressors.

Among the sampled tissues, it is notable that ear has the largest number of overall (555) and tissue specific (336) CCT genes (Figure 1). The greater number of ear CCT genes identified may be related to the profound morphological changes that differentiate the maize and teosinte ear. By comparison, the sampled leaf and stem tissues differ only by size between maize and teosinte and not morphological structure. Similar to our results, an imbalance in number of DE genes in different tissues was also observed in a recent study in *Arabidopsis* [12], where tissues differed by up to 80% in number of DE genes.

### Is there a bias toward increased expression of the maize allele?

In the F_1_ hybrid analysis, ∼55% of genes have higher maize expression than teosinte. Higher expression of the maize allele also occurs in the parent inbred lines, except for leaf, where an equal number of genes favor maize and teosinte alleles. This trend of up-regulated maize expression extends to the CCT gene lists, where ∼60% of genes favor the maize allele (Figure 4). Preferential expression for one of the parents (maize) is consistent with several previous studies in multiple organisms including maize [24], cotton [23], *Arabidopsis* [12], *Cirsium* [7], and fruit fly [11]. While our study mitigates alignment bias with parent specific pseudo-transcriptomes and perfect alignment to segregating sites, this method is unlikely to fully eliminate this bias. Consequently, we cannot exclude the possibility that bias for maize expression is an artifactual result. The mechanism for a general up-regulation of maize alleles across many genes is unclear. One possibility would be a remodeling of the epigenetic landscape during domestication.

### *cis* versus *trans* regulatory divergence

This study shows *cis* and *trans* regulatory differences account for ∼45% and ∼55% of regulatory divergence between maize and teosinte, respectively (Table S1). These values suggest relatively equal contributions of these two mechanisms to regulatory divergence. However, this ignores the contribution of *cis* effects to large expression differences where *cis* accounts for nearly 80% of the expression divergence (Figure 3). The observation that *cis* effects account for the majority of expression divergence of genes with large expression differences was also seen in *Drosophila* [11]. The prominence of *cis* effect among genes with the largest divergence in expression may indicate *cis* regulation is a more effective mechanism than *trans* for producing large changes in gene expression.

A recent study using microarrays [24] showed greater overlap than expected by chance with CCT candidate genes (Table 6). However, the two studies produced largely different lists of DE genes. One difference between the RNAseq and microarray study is that the latter includes DE of genes with *trans* only and *cis*×*trans* regulatory regimes, which are excluded from RNAseq based CCT lists. This difference offers only a partial explanation for the differences between the two studies. Of 262 microarray DE genes assayed by RNAseq in leaf tissue, RNAseq classifies 31 as *trans* only and 16 as *cis*×*trans* (Table S7). Another 153 genes on the microarray DE list are classified as *cis* only or *cis+trans* by RNAseq, leaving 62 genes (24%) for which the two studies disagree. Although much of this disagreement can likely be attributed to factors such as differences in sampling or other sources of error, it reminds us of the imprecision in these types of data.

### Regulatory variation was under selection during domestication

During domestication, maize experienced both a population bottleneck that caused a general reduction in genetic diversity as well as selection that further reduced diversity in targeted regions of the genome [33], [34]. A recent genome-wide analysis estimated that the maize genome possess approximately 81% of the nucleotide diversity found in teosinte [26]. Our data allows us to ask whether maize domestication has caused a parallel reduction in *cis* regulatory variation. Overall, maize possesses only ∼85% of the *cis* regulatory variation seen in teosinte (Figure 5, Table S4), a value very close to the reduction in nucleotide diversity. Moreover, the observed reduction in *cis* regulatory variation is greatest for genes that show evidence for *cis* differences between maize and teosinte. The loss of *cis* regulatory variation increases over C, B and A lists of CCT genes with CCT A-list genes possessing only ∼50% of the *cis* regulatory variation seen in teosinte. This trend suggests selection during the domestication process for *cis* regulatory variation. The high level of expression variation still present in teosinte also represents an untapped source of diversity for maize breeders, which could be explored with transcriptome profiling.

Genomic scans for evidence of selection during adaptive transitions have become a powerful tool in evolutionary biology [35]. Such scans provide both a measure of the prevalence of selection and a list of candidate genes for further study. In comparisons of RNAseq data with a recent genomic selection scan in maize [26], we sought to determine the specific target of selection in terms of *cis* and *trans*. If *cis* regulatory evolution was an important mechanism during maize domestication, then CCT genes should be enriched for selection candidates. In contrast, genes whose expression divergence between maize and teosinte are governed by *trans* effects should not be enriched for selection candidates, given that the *trans* regulators and not the *trans* responsive genes were the putative targets of selection. Consistent with these expectations, we observed a highly significant enrichment for selection candidate genes among CCT and *cis* only genes with no enrichment among *trans* only genes (Tables 4, 5).

Although greater than expected overlap was observed between selection candidates and CCT genes, the degree of correspondence is far from perfect. For example, 25 of 36 CCT A-list genes assessed in the genome selection scan from ear tissue do not show evidence for selection. The misalignment between the selection candidate and CCT lists is likely due to both biological factors and artifacts. Domestication genes such as *tga1* for which the causative change appears to have been an amino acid change will not appear on CCT gene lists [36]. Similarly, genes with *cis* differences that were the target of soft sweeps or for which the signature of selection is weak for other reasons are expected to be missed in selection scans. The candidate for the major gene (*ZmSh5.1*) responsible for the loss of seed shattering during maize domestication is not on the list of selection candidates [26], [37]. These properties of genomic scans remind us of their limits and the probabilistic arguments on which they are based.

### Does domestication favor recessive loss of function alleles?

It has been proposed that the allelic variants responsible for evolution during domestication are often recessive LOF alleles such that the wild progenitor allele would be dominant to the domesticated allele [29]. While there is some support for this hypothesis from rice in the form of increased frequency of deleterious amino acid changes [38], recent reviews of QTL studies found no compelling evidence for dominance of the progenitor alleles and few LOF alleles among positionally cloned domestication genes [39], [40]. There is some support that LOFs are relatively common among genes contributing to varietal differences within crops [39], [40].

Dominance is expected to be uncommon for gene expression of *cis* only genes, since dominance at *cis* only genes requires a mechanism such as transvection [28] and this is unknown in maize. In absence of mechanisms such as transvection, if a CRE in the progenitor allele of a *cis* only gene produced 5 RNAseq reads and disruption of the CRE reduced expression to 1 read per allele in the crop, then the diploid progenitor would have 10 reads, the crop would have 2 reads, and their F_1_ hybrid would have 6 (5+1) reads. The gene would be classified as purely additive. However, for *trans* only genes, if haplo-sufficient, dominance of the maize or teosinte allele could be observed.

Consistent with the first expectation, we found greater dominance among *trans* only genes versus *cis* only genes (Figure 7). The observed dominance effects among the *cis* only genes may be due to statistical error, *trans* effects that are below the level of statistical significance, or a molecular mechanism such as transvection. Among *trans* only genes with dominance, the maize allele is dominant more often than the teosinte allele (Figure 7). This observation fails to support the hypothesis that recessive LOFs were favored during domestication.

### GO terms for transcription activities enriched in *trans* genes

GO term analysis showed genes involved in sequence-specific DNA binding transcription factor activity were enriched in the *trans* only class (Table S14). These *trans* only genes are responding to unknown upstream regulators that differ between maize and teosinte. It is the putative upstream regulators and not the *trans* only genes themselves that are the potential targets of selection during domestication/improvement. This result suggests that transcription factors are frequently downstream in regulatory cascades that were targets of selection during maize domestication and improvement. The identity of the upstream regulators of our *trans* only genes are unknown but they likely include genes involved in signal transduction, hormonal regulation of gene expression, and other transcription factors. The CCT gene lists are candidates for these unknown regulators of the *trans* only genes.

### A catalog of genes with *cis* regulatory variants

A product of this study is a resource for researchers looking for preliminary data on the expression patterns of specific genes. To facilitate this use, two supplemental datasets have been made available from the Dryad Digital Repository: http://dx.doi.org/10.5061/dryad.4kh67. Supplemental dataset 1 contains overall data for the complete set of 25,619 genes including regulatory classification, summed RNAseq read counts, expression ratios, and other summary information for each gene. Supplemental dataset 2 contains read counts for F_1_ hybrid and parent contrasts on a cross-by-cross basis.

An example of the value in these supplemental datasets is *barren stalk1* (*ba1*), a known maize single gene mutant that causes a defect in branch initiation for both the whole plant and tassel [41]. In our study, *ba1* was one of our strongest candidates with all assayed crosses showing higher expression of the maize allele in the ear. The overall shift in expression was substantial (∼4-fold) and this shift was caused solely by *cis* regulatory differences. *ba1* was also found to be under selection during maize domestication in two independent studies [26], [41]. These combined observations suggest that selection for a CRE drove up-regulation of *ba1* in the ear, perhaps resulting in a greater number of rows (branches) of kernels in the maize ear as compared to teosinte. Compelling evidence for this hypothesis could be obtained by fine-mapping and identifying the hypothesized CRE and demonstrating with expression assays that the maize and teosinte alleles of the CRE have the imagined effects on gene expression during ear development and on phenotype (kernel row number) in the adult ear. *ba1* illustrates the power of genomic scans to identify strong candidates for future study that can inform us about the fine details of evolution under domestication.

## Materials and Methods

### Plant material, RNA preparation, and sequencing

Six maize inbred lines, nine teosinte inbred lines, and 29 of their 54 possible maize-teosinte F_1_ hybrids were used in this experiment (Table S15). An average of 1.96 biological replicates of each genotype was used. Plants were grown in growth chambers with a 12 hour dark-light cycle for up to 6 weeks, after which they were moved to a greenhouse. Fifty to 100 milligram samples of the immature ear, leaf, and seedling stem were harvested for RNA extraction during this time. Leaf and seedling stem (including the shoot apical meristem) tissue was collected at the v4 leaf stage. Single ears from maize and F_1_ hybrid plants were collected when the ears weighed 50 to 100 milligrams with silks just beginning to be visible. Teosinte ears were also collected when silks just started to appear, however, due to the small size of teosinte ears 7 to 16 ears (average of 11.27) from each plant were pooled to obtain ∼50 milligrams of tissue. These three tissue types are referred to as the ear, leaf, and stem tissues.

Total RNA was extracted from the plant tissues using a standard TRIzol protocol, quantified by spectrophotometer, and normalized to 1 µg/µL in nuclease free water. Starting with 5 µg total RNA, we generated polyA selected, strand specific, barcoded RNAseq libraries with a previously published protocol using a five minute fragmentation time and 12 PCR amplification cycles [42]. Library adapters used barcode sequences of four and five base pairs (Table S16) designed to balance percent nucleotide composition within the first five base pairs of sequence reads and to have at least two base pair differences from any other barcode. RNAseq libraries were then pooled in groups of 14 (F_1_s) or 15 (parents), and the pooled libraries sequenced on one lane (parents) or two lanes (F_1_s) of an Illumina HiSeq2000 sequencer. The raw sequence data has been deposited in NCBI Sequence Read Archive with accessions SRX710894-711341 and the Gene Expression Omnibus (GEO) Series with accession number GSE61810 (http://www.ncbi.nlm.nih.gov/geo/query/acc.cgi?acc=GSE61810).

### Bioinformatics

Mitigating mapping bias through use of multiple references or enhancing the reference with segregating sites is critical for allele specific studies [43], [44]. We investigated parent specific *de novo* transcriptome assemblies using Trinity [45], but ultimately pursued an enhanced reference approach due to poor Trinity assembly qualities (Text S1). The pipeline developed in this study, based on the work by Wang *et al.* [46], accounts for mapping bias through parent specific pseudo-transcriptomes generated by incorporating polymorphisms derived from non-B73 genomic paired-end reads into the B73 reference followed by alignment and evaluation of RNAseq read depth at segregating sites.

Pseudo-transcriptomes were constructed using the B73 reference genome (version AGPv2) and transcriptome (version ZmB73_5a_WGS) plus ∼403.1 million (17.5× coverage) paired-end genomic sequencing reads from each of the other 14 inbred lines (Table S17). For each of the 14 non-B73 inbreds, paired-end genomic sequencing reads were aligned to the reference genome with the BWA aligner (version 0.5.9) [47]. Only uniquely mapping reads with up to two mismatches were used to limit false polymorphism detection due to paralogous read alignment. Single nucleotide polymorphisms (SNPs) and small insertions and deletions (indels) with respect to B73 were called using the GATK package (version 1.0.5588) [48], [49] and filtered (Text S1) to include only polymorphisms that were homozygous in the inbred with read depth of at least 4×. A strand bias filter was also applied to ensure that the polymorphism was detected on both the plus and minus strand. Polymorphisms surviving these filters were then inserted into the reference B73 transcriptome to make a pseudo-transcriptome for each parent.

For each of the 29 maize-teosinte pairs, a robust set of segregating sites was determined by comparing the pseudo-transcriptomes of the two parents and taking the sites where: the two parental alleles differed, coverage in genomic read alignment was at least four for both parents within the read length (88 bp) of the site, and no heterozygous polymorphisms were detected in genomic read alignments of the two parents within the read length of the site.

RNAseq reads from each F_1_ hybrid and each corresponding pair of inbred parents were then aligned to the combined pseudo-transcriptomes of the two parents (B73 reference transcriptome used for the B73 parent) using the Bowtie aligner (version 0.12.7) [50]. We assessed allele specific expression by counting read depth from each parent at segregating sites (Table S18). Since only perfect alignments were allowed, assignment of reads to parents was straightforward (a read from a given parent could only align to this parent's allele at a segregating site).

### Maize∶teosinte gene expression ratios

We calculated F_1_ hybrid and parent maize∶teosinte expression ratios for each gene of the 29 individual F_1_ hybrid comparisons. Total depth at segregating sites summed over genes was highly correlated between biological replicates (average of 95%, Table S19) and consequently read depth was pooled for the various genotypes. Gene expression ratios for F_1_s (e.g. B73×TIL01) were then calculated by dividing total maize read depth by total teosinte read depth summed over all segregating sites in the gene. The parent expression ratios for individual maize-teosinte comparisons were calculated the same way from parental RNAseq reads, except total parental read depths at segregating sites were corrected for differences in total number of reads between the two parent lines (Text S1). The result of these calculations was a set of 29 matched F_1_ and parent expression ratios consisting of maize∶teosinte read counts for each gene.

We produced overall maize-teosinte expression ratios for each gene by summing read depth over all maize-teosinte hybrid comparisons. To calculate the overall F_1_ expression ratio, the maize and teosinte read depths from the F_1_ hybrids were simply summed over all segregating sites in a gene and across all hybrids. The calculation of the overall parent expression ratio required weighting to avoid counting the parent reads multiple times for each of the F_1_ comparisons in which it was a parent and to compensate for the fact that different parents had variable total numbers of reads (Text S1). Only genes with a read depth of at least 100 in both the F_1_ and its parent comparison were included. The result of these calculations was an overall F_1_ and parent ratio of read counts for each gene. To check whether single F_1_s caused aberrant estimates of the *cis* effect with these overall ratios, we performed a *drop1* analysis and found that inclusion/excluding of single F_1_s had on negligible effects (Text S1 and Figure S5). Finally, as a exercise in proof of concept, we compared allele specific expression results for several specific genes of known importance in domestication with expectations from the literature and found a good fit between our data and published results (Text S1 and Table S20).

### Testing for *cis* and *trans* effects

The combination of F_1_ hybrid and parent inbred expression data allows estimation of both the *cis* and *trans* effects on gene expression. For the F_1_ hybrids, the maize and teosinte alleles at each gene are in a common *trans* cellular environment, and thus any deviation of the maize∶teosinte F_1_ expression ratio from 1∶1 represents purely *cis* effects. By contrast, the maize∶teosinte parent expression ratio is a combination of the *cis* and *trans* effects and any deviation of this ratio from 1∶1 reflects the combined *cis* plus *trans* effects. Therefore, the *trans* effects can be estimated by subtracting the F_1_ hybrid ratio (*cis*) from the parent ratio (*cis* plus *trans*).

Maize and teosinte gene expression as measured by the read depth counts at genes was used for statistical testing of *cis* and *trans* effects. Significant *cis* and *trans* effects were determined using binomial and Fisher's exact tests as described in McManus *et al.* [11]. In brief, two binomial tests were used to identify genes with maize∶teosinte expression ratios significantly different from 1∶1 in the F_1_ hybrid and parent comparisons. Genes with an expression ratio significantly different from 1∶1 for the F_1_ hybrid and/or parent comparison were then subjected to a Fisher's exact test to determine if the parent and F_1_ hybrid maize∶teosinte expression ratios were different from one another. An FDR rate of 0.5% using Storey's q-value [51] was used to compensate for the large number of statistical tests being performed. We investigated a higher FDR cutoff of 5% to include additional genes in downstream analyses, but only observed a minor increase in the number of candidate genes (Text S1, Table S21). The combination of the two binomial tests and Fisher's exact test allowed us to classify each gene into one of seven different regulatory categories (Table 3) as described in McManus *et al.* [11].

### Candidate genes

Genes under selection for expression during maize domestication are expected to show a maize∶teosinte *cis* expression ratio that is significantly different from 1∶1. These genes can fall into the *cis* only (C) or *cis* plus *trans* (CT) groups on Table 3. We call this combined group CCT genes and these differential expression candidates are the focus of many of our analyses.

The list of CCT genes from the overall test was large (5,618 ear; 5,398 leaf; 5,435 stem) and reflects the considerable statistical power to detect slight overall expression biases for genes with thousands of reads aligning to segregating sites. We observed significant maize∶teosinte expression biases as small as 1.02-fold in the overall tests. Such small differences seem unlikely to have biological importance and genes showing these small differences are weak candidates for genes with *cis* expression variation that is causal in maize domestication and improvement. Therefore, we applied filters to identify candidates with the strongest and most consistent regulatory differences.

To narrow down the CCT gene list to candidate genes that show the strongest evidence for differential *cis* regulation between maize and teosinte, we applied two filters. (1) Genes with the strongest evidence for *cis* differences will fall in the CCT group and have data from a majority of sampled maize and teosinte parents. Thus, we filtered the initial list of CCT genes for those with data from at least fifteen F_1_ hybrids that include at least three different maize inbreds and five different teosinte inbreds. (2) For genes with *cis* differences that contributed to maize domestication/improvement, the direction of the expression bias should be highly consistent among the individual F_1_ hybrids. Consequently, CCT genes were classified for consistency of directionality of expression bias among the F_1_s with several levels of candidate genes partitioned at 100%, 90% and 80% of F_1_s showing the same directionality. In calculating these percentages, we used read depth for each F_1_ at the gene to weight the contribution of the F_1_s to the overall percentage. We refer to the CCT genes with 100%, 90% and 80% consistent directionality among the F_1_s as the A-list, B-list and C-list, respectively. For comparative purposes, we made similar A, B and C lists of genes for the *cis* only or *trans* only classes. These CCT and *cis* only gene lists (A, AB, and ABC) were used in downstream analyses in comparison with conserved or *trans* only genes to explore the role of *cis* regulatory variation in maize domestication.

### Proportion of *cis* variation in maize and teosinte

CRE diversity within maize and teosinte is expected to display as variation in the F_1_ hybrid expression ratios. We asked whether *cis* expression variation among F_1_ hybrid ratios was more heavily influenced by maize or teosinte inbred parent. Since three teosinte inbreds (TIL05, TIL10, and TIL15) were involved in only a single F_1_ each, the three F_1_s involving these inbreds were removed in order to balance the number of maize and teosinte inbred parents for this analysis. Genes were tested for variation among the F_1_ expression ratios (*cis* variation) using a linear model on a gene-by-gene basis that fitted the log_2_(maize∶teosinte) F_1_ expression ratio to the maize and teosinte parents as independent variables. Significant maize and teosinte parent terms were identified with an F-test (p<0.05) using the R *drop1* function. The data for each F_1_ was weighted by total depth at the gene to account for variable read depth in the F_1_ hybrids.

### Additive and dominant gene expression

Our gene expression dataset consisting of parent inbred and hybrid expression ratios gives the opportunity to address dominant and additive gene expression on a genome-wide scale. We calculated the additive effect, dominant effect, and dominant/additive (D/A) ratio for each gene and maize-teosinte F_1_ hybrid comparison. The overall maize-teosinte average D/A ratio was then calculated after exclusion of outlier D/A ratios (Figure S6) by iteratively applying the Dixon method [52]. Genes were classified as additive if |D/A|<0.25 and as having dominant gene action if 0.75<|D/A|<1.25. Following calculation of D/A ratios, we examined ratios for *cis* only and *trans* only genes for altered degrees of dominance.

### CCT gene enrichment in various functional categories

We assessed whether genes in different expression classes (CCT, *cis* only and *trans* only) are over or under represented in several functional categories as compared to all genes or genes with conserved expression levels between maize and teosinte. Generally, we tested all CCT gene lists (A, AB, and ABC) with the most weight given to the CCT-AB gene lists, which we consider our best candidates genes. The categories we tested include transcription factors, several metabolic pathways, gene ontology (GO) categories, selection candidates, and domestication QTL. A list of maize transcription factors and their associated families was obtained from the plant transcription factor database [30]. Metabolic enzyme cDNA sequences for starch and lipid metabolism pathways in maize were downloaded from the Kyoto Encyclopedia of Genes and Genomes (KEGG) [53], [54] and matched with genes from the maize filtered gene set (version 5b) by BLAST. Matches (single gene hit with percent identity greater than 95%) were found for 370 out of 379 genes and used to test for enrichment of genes in the various metabolic pathways. Genes under positive selection during maize domestication and improvement were taken from a recent genomic scan for selection [26]. We obtained a list of QTL associated with maize domestication and improvement traits from Table A.1 of a recent QTL analysis [31].

In general, enrichment or depletion of genes in expression classes among various functional categories was tested with Fisher's exact tests on 2×2 contingency tables. For QTL, enrichment of CCT genes among the genes within QTL 1.5 LOD support intervals were tested separately for each trait and only included QTL whose 1.5 LOD support intervals included 20 or fewer genes. For genes under positive selection during domestication and improvement [26], we also tested for a significant difference in the cross population composite likelihood ratio (XPCLR) at the transcription start site between CCT genes (A, AB, and ABC) versus genes with conserved expression using the Kolmogorov-Smirnov and simple t-tests. Finally, GO term enrichment and depletion was tested using the goseq package [55] in R [56] using median gene length to adjust the GO term reference. The base background GO term reference consisted of genes assessed in 15 crosses, three unique maize, and five unique teosinte inbred lines with a cumulative depth of 100 at segregating sites in F_1_ and parent comparisons. GO terms occurring at least five times in the background reference were tested with p-values corrected for multiple testing using the Benjamini-Hochberg method [57].

## Supporting Information

Figure S1 Log_2_ of parent versus hybrid leaf and stem tissue allele-specific expression ratios. The parent (x-axis) versus F_1_ hybrid (y-axis) allele-specific expression ratios are plotted against each other. Regulatory category in terms of the combination of significant statistical tests determined using the method described in methods is shown designated by color. Proportion and count of genes falling into the various regulatory categories are also shown in the lower right hand corner barplot. (A) The leaf tissue analysis included 15,931 genes and displayed a similar pattern to that seen in the ear tissue in Figure 2. (B) The stem tissue analysis of 16,018 genes also showed a similar overall pattern to that seen in leaf and ear.
(TIF)

Figure S2 *Cis* versus estimated *trans* regulatory effect for CCT-ABC genes in the leaf and stem. CCT genes have a directional bias with more genes overall favoring the maize allele than teosinte. Genes with consistent *cis* regulatory differences tend to favor the domesticated maize allele. The (A) leaf tissue and (B) stem tissue display similar bias to that seen in the ear tissue in Figure 4.
(TIF)

Figure S3 Ratio of the average maize to teosinte R^2^ values grouped by regulatory category. In all three tissues, the proportion of maize to teosinte R^2^ varies between 80% and 90%, consistent with the average loss of nucleotide diversity seen during maize domestication. This suggests no single regulatory category captures more genes under selection than others.
(TIF)

Figure S4 Tissue specific density plots of the ln(XPCLR) score for conserved versus CCT candidate genes. CCT (A, AB, and ABC) gene lists have a significantly higher XPCLR score in the 10 kb window holding the transcriptional start site than conserved (Con) genes. The natural log transformed XPCLR scores for CCT genes are progressively statistically higher than genes identified as conserved in the initial analysis. The distributions of conserved and CCT genes are significantly different by both the shape sensitive Kolmogorov-Smirnov test (upper right of heatmap) and simple difference of the means t-test (lower right of heatmap). Heatmap scale is in log_10_(p-value).
(TIF)

Figure S5 Density plots of the average difference (by gene) in *cis* effect caused by dropping read depth from a single F_1_. The average difference in *cis* effect for each assessed gene caused by dropping read depth from each F_1_ one by one was calculated. Density plots show a strong peak at zero with minimal variance, suggesting individual F_1_ read depths have minor effect on the overall expression ratio.
(TIF)

Figure S6 Histogram of the number of outlier D/A ratios removed by the Dixon method. Cross specific D/A ratios were calculated for all genes and outlier D/A ratios were excluded by iteratively applying the Dixon method. The majority of cases removed two or fewer outliers.
(TIF)

Table S1 Proportion of divergence due to *cis* regulatory effect grouped by overall parental divergence calculated as described in McManus *et al.* [11].
(DOCX)

Table S2 The number of genes for which the maize or teosinte allele is expressed at a higher level.
(DOCX)

Table S3 Bias for the maize allele grouped by inbred line for the three tissues in the CCT-ABC gene list.
(DOCX)

Table S4 Proportion of the variation among the maize∶teosinte expression ratios for the F_1_ hybrids that is explained by maize and teosinte parents.
(DOCX)

Table S5 Number of genes for which the maize and/or teosinte parent contributed to the variance among the F1 hybrid gene expression ratios (heterogeneous) and genes for which there was no variance in expression attributable to the maize or teosinte parent (homogeneous). CCT genes in groups A, B, and C in the three tissue types are shown.
(DOCX)

Table S6 Comparison of observed and expected numbers of genes classified as differentially expressed (DE) or not differentially expressed (NDE) by RNAseq [this study] and microarray assays [24] in groups A, B, and C in the three tissue types.
(DOCX)

Table S7 RNAseq-based regulatory categories for genes identified as differentially expressed in the microarray study [24].
(DOCX)

Table S8 Fisher's exact tests for the overlap between genes associated with regions that are differentially methylated in maize and teosinte (DMRs) [27] and CCT-ABC genes from each of the three experimental tissues in our work.
(DOCX)

Table S9 Number of candidate genes neighboring regions that are differentially methylated (DMRs) between maize and teosinte [27] and proportion in which the RNAseq expression data agrees with methylated status.
(DOCX)

Table S10 Additive and dominant gene counts for the A, AB, and ABC *cis* and *trans* only candidate lists. Dominance cells contain the number of genes for which the maize∶teosinte allele was dominant. Fisher's exact tests (FET) interrogate whether the degree of dominance/additivity differs between the *cis* and *trans* classes. A binomial test (*) indicates significantly different maize∶teosinte count (p<0.005).
(DOCX)

Table S11 Degree of overlap between our CCT (AB list) genes and genes in different transcription factor families.
(DOCX)

Table S12 Degree of overlap between CCT (AB list) differentially expressed genes and genes in the 1.5 support intervals for QTL from a previous study.
(DOCX)

Table S13 Degree overlap between our CCT and *trans* only differentially expressed genes and genes in metabolic pathways defined in KEGG.
(DOCX)

Table S14 Significantly enriched and depleted GO terms from CCT and *trans* only gene lists including tissue, group, accession, description, counts, rate of occurrence, and FDR corrected p-values.
(DOCX)

Table S15 Numbers of biological replicates of F_1_ hybrid and parent inbred lines for RNAseq expression study with hybrid replicates internal and parent around the perimeter (shaded gray).
(DOCX)

Table S16 Adapter name, barcode sequence, and barcode length for Illumina adapters used in RNAseq libraries.
(DOCX)

Table S17 Number of genomic paired end reads and coverage obtained for constructing pseudo-transcriptomes.
(DOCX)

Table S18 F_1_ and parent maize∶teosinte read depth ratios for the 29 maize-teosinte comparisons.
(DOCX)

Table S19 Spearman correlation between all biological replicate pairs of the same genotype.
(DOCX)

Table S20 Comparison of RNAseq expression data with expectations identified in the literature.
(DOCX)

Table S21 Increase in size and percent of CCT A, B, and C gene lists when using 5% Storey's FDR for significant binomial and Fisher's exact tests.
(DOCX)

Text S1 Supplemental methods and results.
(DOCX)

## References

[1] Carroll SB (2005) Evolution at two levels: on genes and form. PLoS Biol 3: e245 10.1371/journal.pbio.0030245 16000021 PMC1174822

[2] Carroll SB (2008) Evo-devo and an expanding evolutionary synthesis: a genetic theory of morphological evolution. Cell 134: 25–36 10.1016/j.cell.2008.06.030 18614008

[3] Wittkopp PJ, Kalay G (2012) Cis-regulatory elements: molecular mechanisms and evolutionary processes underlying divergence. Nat Rev Genet 13: 59–69 10.1038/nrg3095 22143240

[4] Stern DL, Orgogozo V (2008) The loci of evolution: how predictable is genetic evolution? Evolution (N Y) 62: 2155–2177 10.1111/j.1558-5646.2008.00450.x PMC2613234 18616572

[5] Springer NM, Stupar RM (2007) Allele-specific expression patterns reveal biases and embryo-specific parent-of-origin effects in hybrid maize. Plant Cell 19: 2391–2402 10.1105/tpc.107.052258 17693532 PMC2002603

[6] Zhang X, Borevitz JO (2009) Global analysis of allele-specific expression in Arabidopsis thaliana. Genetics 182: 943–954 10.1534/genetics.109.103499 19474198 PMC2728882

[7] Bell GDM, Kane NC, Rieseberg LH, Adams KL (2013) RNA-seq analysis of allele-specific expression, hybrid effects, and regulatory divergence in hybrids compared with their parents from natural populations. Genome Biol Evol 5: 1309–1323 10.1093/gbe/evt072 23677938 PMC3730339

[8] Song G, Guo Z, Liu Z, Cheng Q, Qu X, et al (2013) Global RNA sequencing reveals that genotype-dependent allele-specific expression contributes to differential expression in rice F1 hybrids. BMC Plant Biol 13: 221 10.1186/1471-2229-13-221 24358981 PMC3878109

[9] Tirosh I, Reikhav S, Levy A a, Barkai N (2009) A yeast hybrid provides insight into the evolution of gene expression regulation. Science (80-) 324: 659–662 10.1126/science.1169766 19407207

[10] Emerson JJ, Hsieh L-C, Sung H-M, Wang T-Y, Huang C-J, et al (2010) Natural selection on cis and trans regulation in yeasts. Genome Res 20: 826–836 10.1101/gr.101576.109 20445163 PMC2877579

[11] McManus CJ, Coolon JD, Duff MO, Eipper-Mains J, Graveley BR, et al (2010) Regulatory divergence in Drosophila revealed by mRNA-seq. Genome Res 20: 816–825 10.1101/gr.102491.109 20354124 PMC2877578

[12] He F, Zhang X, Hu J, Turck F, Dong X, et al (2012) Genome-wide Analysis of Cis-regulatory Divergence between Species in the Arabidopsis Genus. Mol Biol Evol 29: 3385–3395 10.1093/molbev/mss146 22641789

[13] Schaefke B, Emerson JJ, Wang T-Y, Lu M-YJ, Hsieh L-C, et al (2013) Inheritance of gene expression level and selective constraints on trans- and cis-regulatory changes in yeast. Mol Biol Evol 30: 2121–2133 10.1093/molbev/mst114 23793114

[14] Doebley JF, Gaut BS, Smith BD (2006) The molecular genetics of crop domestication. Cell 127: 1309–1321 10.1016/j.cell.2006.12.006 17190597

[15] Purugganan MD, Fuller DQ (2009) The nature of selection during plant domestication. Nature 457: 843–848 10.1038/nature07895 19212403

[16] Olsen KM, Wendel JF (2013) A bountiful harvest: genomic insights into crop domestication phenotypes. Annu Rev Plant Biol 64: 47–70 10.1146/annurev-arplant-050312-120048 23451788

[17] Studer A, Zhao Q, Ross-Ibarra J, Doebley J (2011) Identification of a functional transposon insertion in the maize domestication gene tb1. Nat Genet 43: 1160–1163 10.1038/ng.942 21946354 PMC3686474

[18] Yang Q, Li Z, Li W, Ku L, Wang C, et al (2013) CACTA-like transposable element in ZmCCT attenuated photoperiod sensitivity and accelerated the postdomestication spread of maize. Proc Natl Acad Sci 110: 16969–16974 10.1073/pnas.1310949110 24089449 PMC3801022

[19] Lang Z, Wills DM, Lemmon ZH, Shannon LM, Bukowski R, et al (2014) Defining the Role of prolamin-box binding factor1 Gene During Maize Domestication. J Hered 10.1093/jhered/esu019 PMC6373496 24683184

[20] Konishi S, Izawa T, Lin SY, Ebana K, Fukuta Y, et al (2006) An SNP caused loss of seed shattering during rice domestication. Science (80-) 312: 1392–1396 10.1126/science.1126410 16614172

[21] Wills DM, Whipple CJ, Takuno S, Kursel LE, Shannon LM, et al (2013) From many, one: genetic control of prolificacy during maize domestication. PLoS Genet 9: e1003604 10.1371/journal.pgen.1003604 23825971 PMC3694832

[22] Frary A, Nesbitt TC, Grandillo S, Knaap E, Cong B, et al (2000) fw2.2: a quantitative trait locus key to the evolution of tomato fruit size. Science 289: 85–88 10.1126/science.289.5476.85 10884229

[23] Rapp RA, Haigler CH, Flagel L, Hovav RH, Udall JA, et al (2010) Gene expression in developing fibres of Upland cotton (Gossypium hirsutum L.) was massively altered by domestication. BMC Biol 8: 139 10.1186/1741-7007-8-139 21078138 PMC2992495

[24] Swanson-Wagner R, Briskine R, Schaefer R, Hufford MB, Ross-Ibarra J, et al (2012) Reshaping of the maize transcriptome by domestication. Proc Natl Acad Sci 109: 11878–11883 10.1073/pnas.1201961109 22753482 PMC3406829

[25] Koenig D, Jiménez-Gómez JM, Kimura S, Fulop D, Chitwood DH, et al (2013) Comparative transcriptomics reveals patterns of selection in domesticated and wild tomato. Proc Natl Acad Sci 110: E2655–62 10.1073/pnas.1309606110 23803858 PMC3710864

[26] Hufford MB, Xu X, van Heerwaarden J, Pyhäjärvi T, Chia J-M, et al (2012) Comparative population genomics of maize domestication and improvement. Nat Genet 44: 808–811 10.1038/ng.2309 22660546 PMC5531767

[27] Eichten SR, Briskine R, Song J, Li Q, Swanson-Wagner R, et al (2013) Epigenetic and genetic influences on DNA methylation variation in maize populations. Plant Cell 25: 2783–2797 10.1105/tpc.113.114793 23922207 PMC3784580

[28] Duncan IW (2002) Transvection effects in Drosophila. Annu Rev Genet 36: 521–556 10.1146/annurev.genet.36.060402.100441 12429702

[29] Lester RN (1989) Evolution under domestication involving disturbance of genic balance. Euphytica 44: 125–132 10.1007/BF00022606

[30] Jin J, Zhang H, Kong L, Gao G, Luo J (2014) PlantTFDB 3.0: a portal for the functional and evolutionary study of plant transcription factors. Nucleic Acids Res 42: D1182–7 10.1093/nar/gkt1016 24174544 PMC3965000

[31] Shannon LM (2012) The Genetic Architecture of Maize Domestication and Range Expansion. Ph.D. dissertation. University of Wisconsin - Madison.

[32] Hansey CN, Vaillancourt B, Sekhon RS, de Leon N, Kaeppler SM, et al (2012) Maize (Zea mays L.) genome diversity as revealed by RNA-sequencing. PLoS One 7: e33071 10.1371/journal.pone.0033071 22438891 PMC3306378

[33] Tenaillon MI, U'Ren J, Tenaillon O, Gaut BS (2004) Selection versus demography: a multilocus investigation of the domestication process in maize. Mol Biol Evol 21: 1214–1225 10.1093/molbev/msh102 15014173

[34] Eyre-Walker A, Keightley PD (2009) Estimating the rate of adaptive molecular evolution in the presence of slightly deleterious mutations and population size change. Mol Biol Evol 26: 2097–2108 10.1093/molbev/msp119 19535738

[35] Strasburg JL, Sherman NA, Wright KM, Moyle LC, Willis JH, et al (2012) What can patterns of differentiation across plant genomes tell us about adaptation and speciation? Philos Trans R Soc Lond B Biol Sci 367: 364–373 10.1098/rstb.2011.0199 22201166 PMC3233712

[36] Wang H, Nussbaum-Wagler T, Li B, Zhao Q, Vigouroux Y, et al (2005) The origin of the naked grains of maize. Nature 436: 714–719 10.1038/nature03863 16079849 PMC1464477

[37] Lin Z, Li X, Shannon LM, Yeh C-T, Wang ML, et al (2012) Parallel domestication of the Shattering1 genes in cereals. Nat Genet 44: 720–724 10.1038/ng.2281 22581231 PMC3532051

[38] Lu J, Tang T, Tang H, Huang J, Shi S, et al (2006) The accumulation of deleterious mutations in rice genomes: a hypothesis on the cost of domestication. Trends Genet 22: 126–131 10.1016/j.tig.2006.01.004 16443304

[39] Burger JC, Chapman MA, Burke JM (2008) Molecular insights into the evolution of crop plants. Am J Bot 95: 113–122 10.3732/ajb.95.2.113 21632337

[40] Gross BL, Olsen KM (2010) Genetic perspectives on crop domestication. Trends Plant Sci 15: 529–537 10.1016/j.tplants.2010.05.008 20541451 PMC2939243

[41] Gallavotti A, Zhao Q, Kyozuka J, Meeley RB, Ritter MK, et al (2004) The role of barren stalk1 in the architecture of maize. Nature 432: 630–635 10.1038/nature03148 15577912

[42] Zhong S, Joung J, Zheng Y, Chen Y, Liu B, et al (2011) High-throughput illumina strand-specific RNA sequencing library preparation. Cold Spring Harb Protoc 2011: 940–949 10.1101/pdb.prot5652 21807852

[43] Satya RV, Zavaljevski N, Reifman J (2012) A new strategy to reduce allelic bias in RNA-Seq readmapping. Nucleic Acids Res 40: e127 10.1093/nar/gks425 22584625 PMC3439884

[44] Huang S, Holt J, Kao C-Y, McMillan L, Wang W (2014) A novel multi-alignment pipeline for high-throughput sequencing data. Database (Oxford) 2014: 1–12 10.1093/database/bau057 PMC4062837 24948510

[45] Grabherr MG, Haas BJ, Yassour M, Levin JZ, Thompson DA, et al (2011) Full-length transcriptome assembly from RNA-Seq data without a reference genome. Nat Biotechnol 29: 644–652 10.1038/nbt.1883 21572440 PMC3571712

[46] Wang X, Soloway PD, Clark AG (2011) A survey for novel imprinted genes in the mouse placenta by mRNA-seq. Genetics 189: 109–122 10.1534/genetics.111.130088 21705755 PMC3176116

[47] Li H, Durbin R (2009) Fast and accurate short read alignment with Burrows-Wheeler transform. Bioinformatics 25: 1754–1760 10.1093/bioinformatics/btp324 19451168 PMC2705234

[48] McKenna A, Hanna M, Banks E, Sivachenko A, Cibulskis K, et al (2010) The Genome Analysis Toolkit: a MapReduce framework for analyzing next-generation DNA sequencing data. Genome Res 20: 1297–1303 10.1101/gr.107524.110 20644199 PMC2928508

[49] DePristo MA, Banks E, Poplin R, Garimella KV, Maguire JR, et al (2011) A framework for variation discovery and genotyping using next-generation DNA sequencing data. Nat Genet 43: 491–498 10.1038/ng.806 21478889 PMC3083463

[50] Langmead B, Trapnell C, Pop M, Salzberg SL (2009) Ultrafast and memory-efficient alignment of short DNA sequences to the human genome. Genome Biol 10: R25 10.1186/gb-2009-10-3-r25 19261174 PMC2690996

[51] Storey JD (2002) A direct approach to false discovery rates. J R Stat Soc Ser B (Statistical Methodol 64: 479–498 10.1111/1467-9868.00346

[52] Dean RB, Dixon WJ (1951) Simplified Statistics for Small Numbers of Observations. Anal Chem 23: 636–638 10.1021/ac60052a025

[53] Kanehisa M (2000) KEGG: Kyoto Encyclopedia of Genes and Genomes. Nucleic Acids Res 28: 27–30 10.1093/nar/28.1.27 10592173 PMC102409

[54] Kanehisa M, Goto S, Sato Y, Furumichi M, Tanabe M (2012) KEGG for integration and interpretation of large-scale molecular data sets. Nucleic Acids Res 40: D109–14 10.1093/nar/gkr988 22080510 PMC3245020

[55] Young MD, Wakefield MJ, Smyth GK, Oshlack A (2010) Gene ontology analysis for RNA-seq: accounting for selection bias. Genome Biol 11: R14 10.1186/gb-2010-11-2-r14 20132535 PMC2872874

[56] R Development Core Team (2013) R: A language and environment for statistical computing.

[57] Benjamini Y, Hochberg Y (1995) Controlling the false discovery rate: a practical and powerful approach to multiple testing. J R Stat Soc Ser B 57: 289–300.

