# Supplementary figures and images for: The Role of cis Regulatory Evolution in Maize Domestication
Source: PLoS Genet. 2014 Nov 6;10(11):e1004745. doi: 10.1371/journal.pgen.1004745 (PMC4222645; doi:10.1371/journal.pgen.1004745)

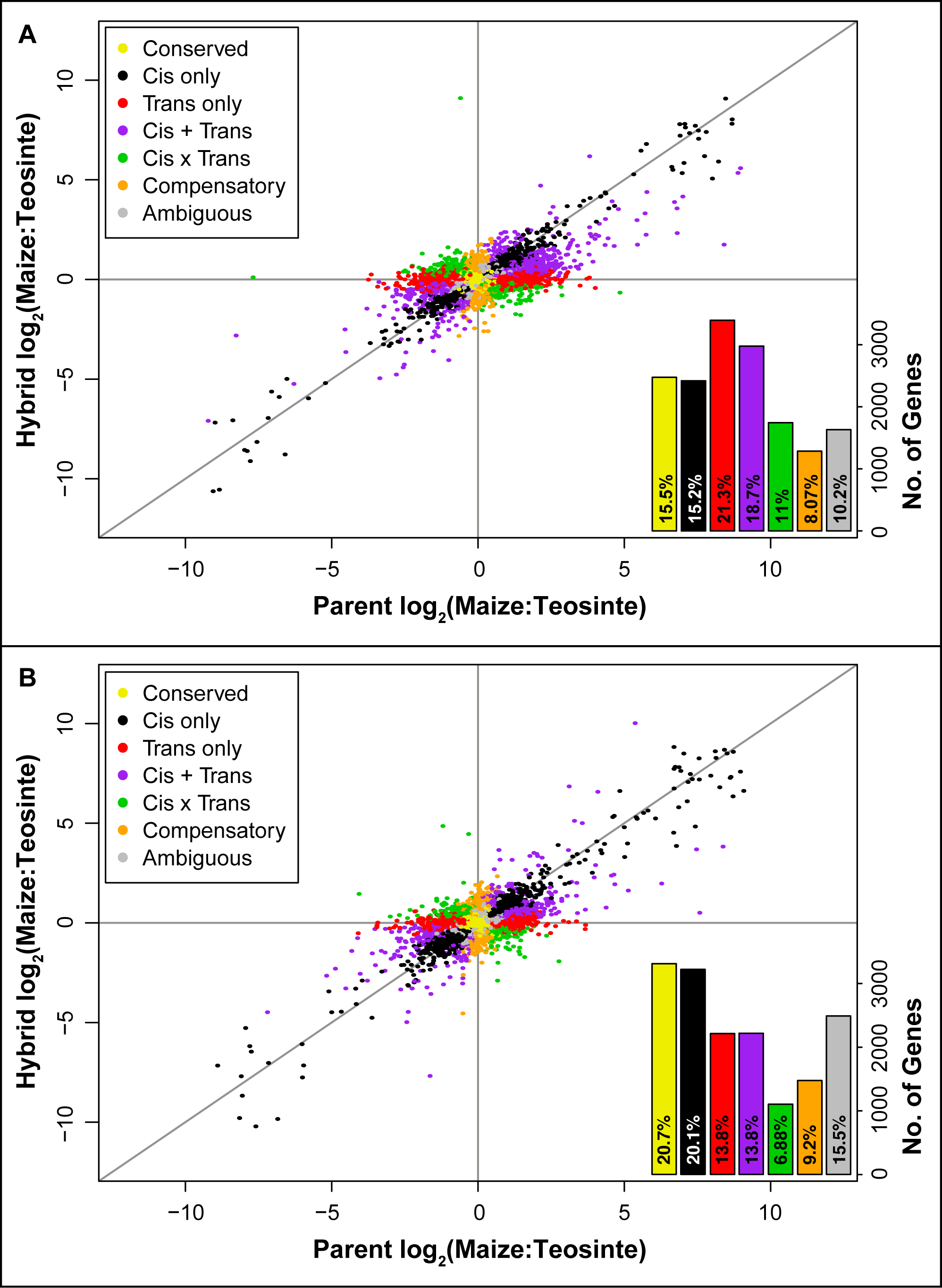

Supplement: Figure S1 — Log2 of parent versus hybrid leaf and stem tissue allele-specific expression ratios. The parent (x-axis) versus F1 hybrid (y-axis) allele-specific expression ratios are plotted against each other. Regulatory category in terms of the combination of significant statistical tests determined using the method described in methods is shown designated by color. Proportion and count of genes falling into the various regulatory categories are also shown in the lower right hand corner barplot. (A) The leaf tissue analysis included 15,931 genes and displayed a similar pattern to that seen in the ear tissue in Figure 2. (B) The stem tissue analysis of 16,018 genes also showed a similar overall pattern to that seen in leaf and ear. (TIF) [file pgen.1004745.s001.tif]

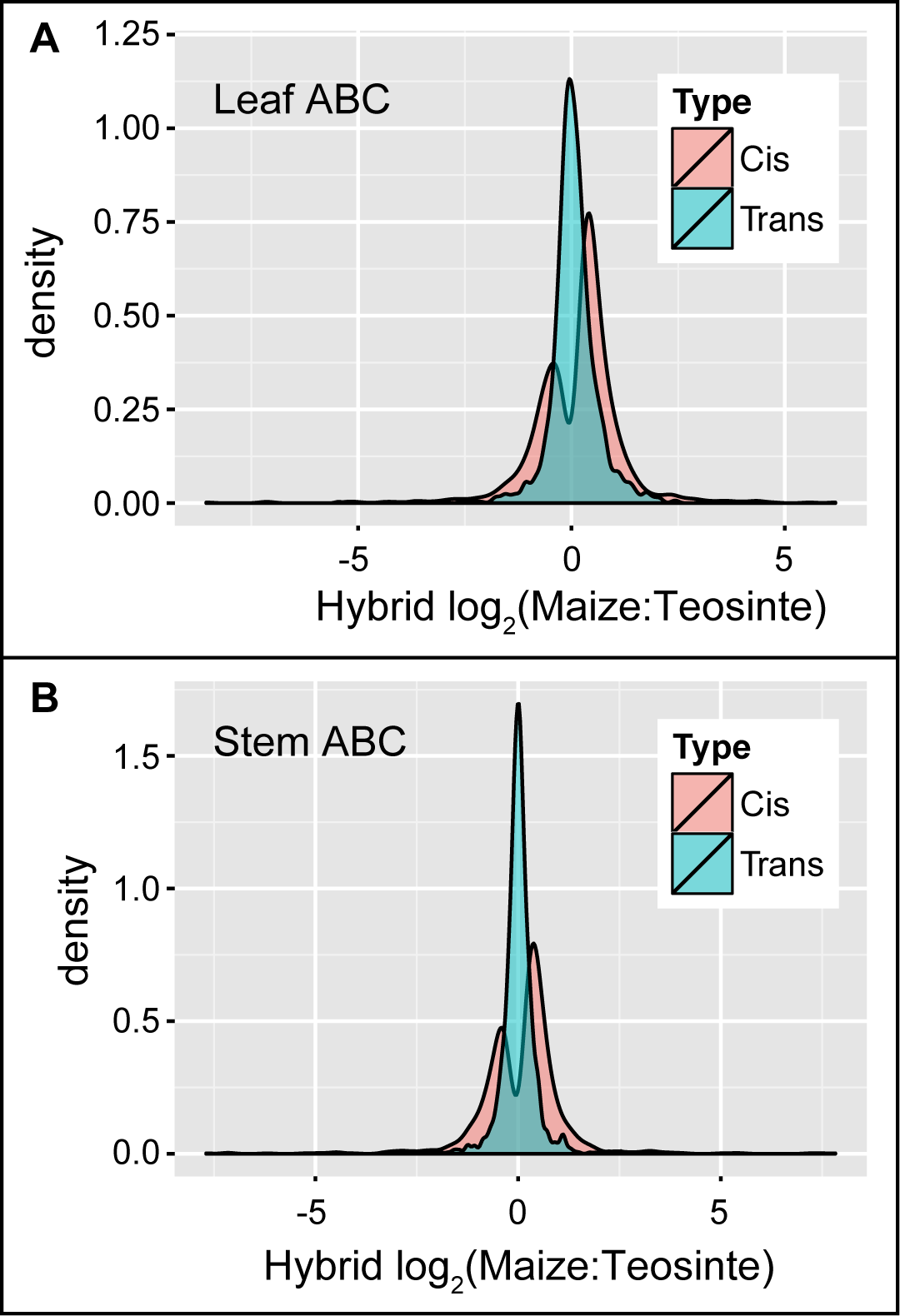

Supplement: Figure S2 — Cis versus estimated trans regulatory effect for CCT-ABC genes in the leaf and stem. CCT genes have a directional bias with more genes overall favoring the maize allele than teosinte. Genes with consistent cis regulatory differences tend to favor the domesticated maize allele. The (A) leaf tissue and (B) stem tissue display similar bias to that seen in the ear tissue in Figure 4. (TIF) [file pgen.1004745.s002.tif]

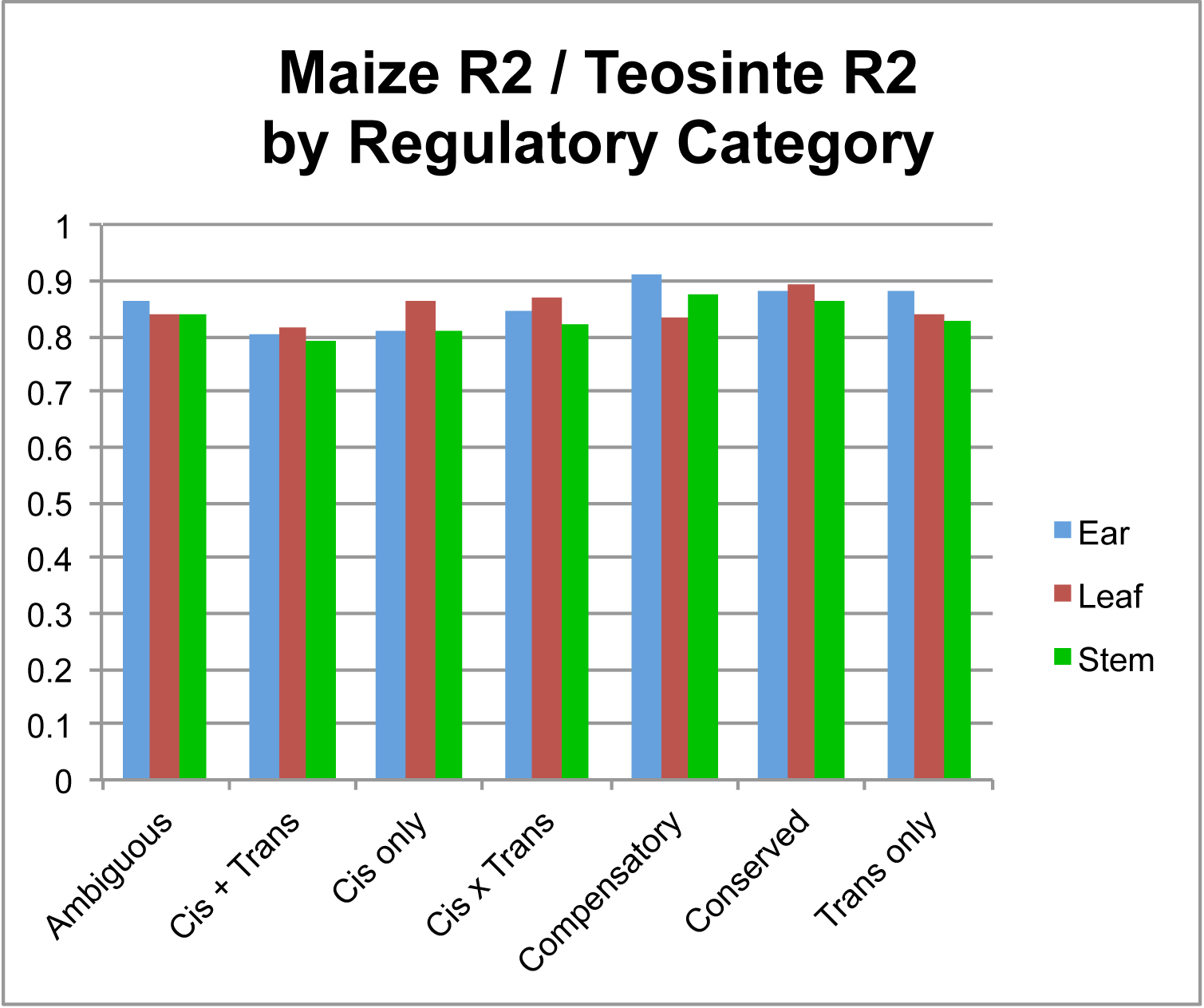

Supplement: Figure S3 — Ratio of the average maize to teosinte R2 values grouped by regulatory category. In all three tissues, the proportion of maize to teosinte R2 varies between 80% and 90%, consistent with the average loss of nucleotide diversity seen during maize domestication. This suggests no single regulatory category captures more genes under selection than others. (TIF) [file pgen.1004745.s003.tif]

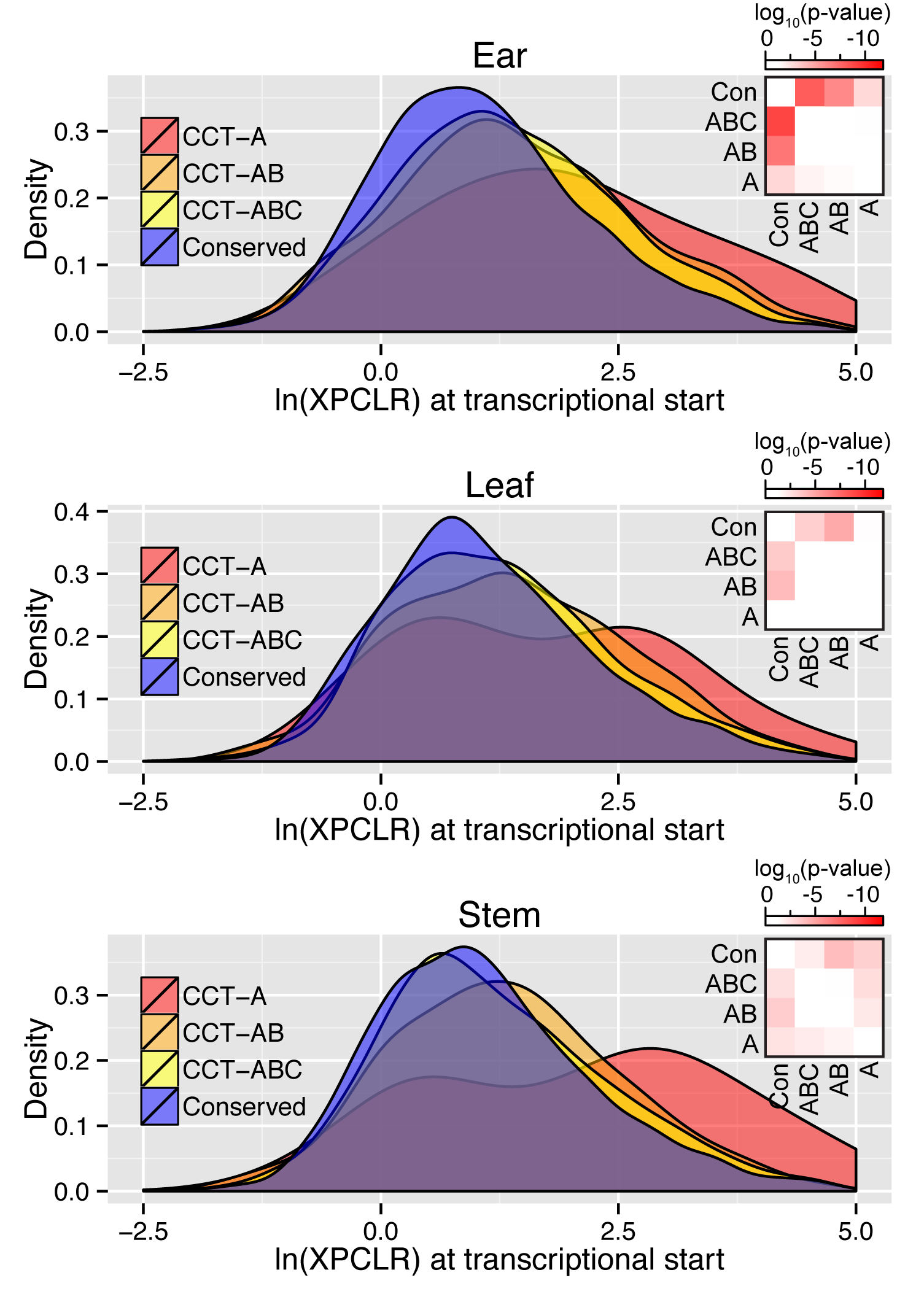

Supplement: Figure S4 — Tissue specific density plots of the ln(XPCLR) score for conserved versus CCT candidate genes. CCT (A, AB, and ABC) gene lists have a significantly higher XPCLR score in the 10 kb window holding the transcriptional start site than conserved (Con) genes. The natural log transformed XPCLR scores for CCT genes are progressively statistically higher than genes identified as conserved in the initial analysis. The distributions of conserved and CCT genes are significantly different by both the shape sensitive Kolmogorov-Smirnov test (upper right of heatmap) and simple difference of the means t-test (lower right of heatmap). Heatmap scale is in log10(p-value). (TIF) [file pgen.1004745.s004.tif]
