# Supplementary material for: The Role of cis Regulatory Evolution in Maize Domestication
Source: PLoS Genet. 2014 Nov 6;10(11):e1004745. doi: 10.1371/journal.pgen.1004745 (PMC4222645; doi:10.1371/journal.pgen.1004745)
Supplement: Table S1 — Proportion of divergence due to cis regulatory effect grouped by overall parental divergence calculated as described in McManus et al. [11]. (DOCX) [file pgen.1004745.s007.docx]

Table S1: Proportion of divergence due to *cis* regulatory effect grouped by overall parental divergence calculated as described in McManus *et al.* [11].

| Gene Group^1^ | N | Tissue | % *cis* ± SE |
| --- | --- | --- | --- |
| All genes | 15939 | Ear | 0.4519 ± 0.0021 |
| 0-1 | 14140 | Ear | 0.4583 ± 0.0022 |
| 1-2 | 1312 | Ear | 0.3918 ± 0.0081 |
| 2-3 | 268 | Ear | 0.3524 ± 0.0188 |
| 3-4 | 95 | Ear | 0.3370 ± 0.0298 |
| 4-5 | 45 | Ear | 0.4713 ± 0.0495 |
| 5+ | 79 | Ear | 0.7777 ± 0.0273 |
| All genes | 15931 | Leaf | 0.4151 ± 0.0021 |
| 0-1 | 13787 | Leaf | 0.4246 ± 0.0022 |
| 1-2 | 1740 | Leaf | 0.3311 ± 0.0066 |
| 2-3 | 278 | Leaf | 0.3732 ± 0.0174 |
| 3-4 | 51 | Leaf | 0.4522 ± 0.0445 |
| 4-5 | 21 | Leaf | 0.6476 ± 0.0584 |
| 5+ | 54 | Leaf | 0.7679 ± 0.0301 |
| All genes | 16018 | Stem | 0.4704 ± 0.0021 |
| 0-1 | 14746 | Stem | 0.4715 ± 0.0022 |
| 1-2 | 1000 | Stem | 0.4284 ± 0.0096 |
| 2-3 | 149 | Stem | 0.4629 ± 0.0233 |
| 3-4 | 40 | Stem | 0.5051 ± 0.0539 |
| 4-5 | 23 | Stem | 0.6365 ± 0.0590 |
| 5+ | 60 | Stem | 0.8081 ± 0.0248 |

^1^ Group (except for “All genes”) indicates grouping of genes by the absolute value of the parent log_2_(Maize:Teosinte) ratio.
