# Supplementary material for: The Role of cis Regulatory Evolution in Maize Domestication
Source: PLoS Genet. 2014 Nov 6;10(11):e1004745. doi: 10.1371/journal.pgen.1004745 (PMC4222645; doi:10.1371/journal.pgen.1004745)
Supplement: Table S2 — The number of genes for which the maize or teosinte allele is expressed at a higher level. (DOCX) [file pgen.1004745.s008.docx]

Table S2: The number of genes for which the maize or teosinte allele is expressed at a higher level in F_1_ hybrids.

| **CCT Group** | **Tissue** | **+log2(M/T)** | **Zero log2(M/T)** | **-log2(M/T)** |
| --- | --- | --- | --- | --- |
| A | Ear | 9 | 0 | 34 |
| A | Leaf | 5 | 0 | 17 |
| A | Stem | 8 | 0 | 19 |
| B | Ear | 193 | 0 | 319 |
| B | Leaf | 164 | 0 | 272 |
| B | Stem | 155 | 0 | 249 |
| C | Ear | 396 | 0 | 594 |
| C | Leaf | 314 | 0 | 555 |
| C | Stem | 382 | 0 | 558 |
| AB | Ear | 202 | 0 | 353 |
| AB | Leaf | 169 | 0 | 289 |
| AB | Stem | 163 | 0 | 268 |
| ABC | Ear | 598 | 0 | 947 |
| ABC | Leaf | 483 | 0 | 844 |
| ABC | Stem | 545 | 0 | 826 |
| Overall | Ear | 5870 | 38 | 7286 |
| Overall | Leaf | 5832 | 25 | 7307 |
| Overall | Stem | 5990 | 21 | 7294 |
