# Supplementary material for: The Role of cis Regulatory Evolution in Maize Domestication
Source: PLoS Genet. 2014 Nov 6;10(11):e1004745. doi: 10.1371/journal.pgen.1004745 (PMC4222645; doi:10.1371/journal.pgen.1004745)
Supplement: Table S3 — Bias for the maize allele grouped by inbred line for the three tissues in the CCT-ABC gene list. (DOCX) [file pgen.1004745.s009.docx]

Table S3: Bias for the maize allele grouped by inbred line for the three tissues in the CCT-ABC gene list.

| **Maize Inbred** | **CCT Group** | **Tissue** | **Teosinte Bias** | **No Bias** | **Maize Bias** | **M:T Ratio** |
| --- | --- | --- | --- | --- | --- | --- |
| B73 | ABC | Ear^a^ | 569 | 1 | 975 | 1.713533 |
| CML103 |  |  | 661 | 6 | 839 | 1.269289 |
| Ki3 |  |  | 602 | 5 | 915 | 1.519934 |
| Mo17 |  |  | 605 | 12 | 845 | 1.396694 |
| Oh43 |  |  | 594 | 1 | 949 | 1.597643 |
| W22 |  |  | 640 | 4 | 889 | 1.389063 |
| non-B73 |  |  | 606 | 0 | 939 | 1.549505 |
| B73 | ABC | Leaf^b^ | 466 | 0 | 861 | 1.847639 |
| CML103 |  |  | 570 | 6 | 711 | 1.247368 |
| Ki3 |  |  | 506 | 5 | 795 | 1.571146 |
| Mo17 |  |  | 496 | 4 | 785 | 1.582661 |
| Oh43 |  |  | 478 | 0 | 846 | 1.769874 |
| W22 |  |  | 507 | 2 | 809 | 1.595660 |
| non-B73 |  |  | 505 | 0 | 822 | 1.627722 |
| B73 | ABC | Stem^c^ | 524 | 0 | 847 | 1.616412 |
| CML103 |  |  | 582 | 7 | 739 | 1.269759 |
| Ki3 |  |  | 555 | 2 | 793 | 1.428829 |
| Mo17 |  |  | 520 | 4 | 806 | 1.550000 |
| Oh43 |  |  | 512 | 1 | 857 | 1.673828 |
| W22 |  |  | 546 | 1 | 814 | 1.490842 |
| non-B73 |  |  | 545 | 0 | 826 | 1.515596 |

^a^ Fisher’s exact test for B73 versus cumulative non-B73 ratio, p = 0.1824.

^b^ Fisher’s exact test for B73 versus cumulative non-B73 ratio, p = 0.1256.

^c^ Fisher’s exact test for B73 versus cumulative non-B73 ratio, p = 0.4332.
