# Supplementary material for: The Role of cis Regulatory Evolution in Maize Domestication
Source: PLoS Genet. 2014 Nov 6;10(11):e1004745. doi: 10.1371/journal.pgen.1004745 (PMC4222645; doi:10.1371/journal.pgen.1004745)
Supplement: Table S4 — Proportion of the variation among the maize∶teosinte expression ratios for the F1 hybrids that is explained by maize and teosinte parents. (DOCX) [file pgen.1004745.s010.docx]

Table S4: Proportion of the variation among the maize:teosinte expression ratios for the F_1_ hybrids that is explained by maize and teosinte parents.

| **Tissue** | **Category** | **R^2^ maize** | **R^2^ teosinte** | **Maize/Teosinte** | **Gene Count** |
| --- | --- | --- | --- | --- | --- |
| Ear | All genes | 32.48% | 38.21% | 85.01% | 13194 |
| Leaf | All genes | 32.11% | 37.85% | 84.83% | 13164 |
| Stem | All genes | 32.04% | 38.56% | 83.09% | 13305 |
| Ear | ABC | 32.25% | 41.37% | 77.96% | 1545 |
| Leaf | ABC | 32.07% | 40.91% | 78.38% | 1327 |
| Stem | ABC | 32.20% | 41.26% | 78.05% | 1371 |
| Ear | AB | 30.76% | 42.95% | 71.64% | 555 |
| Leaf | AB | 31.14% | 42.64% | 73.03% | 458 |
| Stem | AB | 32.28% | 42.22% | 76.45% | 431 |
| Ear | A | 26.58% | 48.86% | 54.41% | 43 |
| Leaf | A | 23.17% | 48.78% | 47.50% | 22 |
| Stem | A | 28.86% | 48.26% | 59.80% | 27 |
