# Supplementary material for: The Role of cis Regulatory Evolution in Maize Domestication
Source: PLoS Genet. 2014 Nov 6;10(11):e1004745. doi: 10.1371/journal.pgen.1004745 (PMC4222645; doi:10.1371/journal.pgen.1004745)
Supplement: Table S5 — Number of genes for which the maize and/or teosinte parent contributed to the variance among the F1 hybrid gene expression ratios (heterogeneous) and genes for which there was no variance in expression attributable to the maize or teosinte parent (homogeneous). CCT genes in groups A, B, and C in the three tissue types are shown. (DOCX) [file pgen.1004745.s011.docx]

Table S5: Number of genes for which the maize and/or teosinte parent contributed to the variance among the F1 hybrid gene expression ratios (heterogeneous) and genes for which there was no variance in expression attributable to the maize or teosinte parent (homogeneous). CCT genes in groups A, B, and C in the three tissue types are shown.

| **Tissue** | **Category** | **Heterogeneous** | | | **Homogenous** | **Total** |
| --- | --- | --- | --- | --- | --- | --- |
|  |  | **Maize** | **Teosinte** | **Maize+Teosinte** |  |  |
| Ear | All genes | 1880 | 2959 | 2504 | 5851 | 13194 |
| Leaf | All genes | 1837 | 3093 | 2517 | 5717 | 13164 |
| Stem | All genes | 1924 | 3215 | 2645 | 5521 | 13305 |
| Ear | ABC | 195 | 417 | 350 | 583 | 1545 |
| Leaf | ABC | 167 | 364 | 305 | 491 | 1327 |
| Stem | ABC | 193 | 374 | 321 | 483 | 1371 |
| Ear | AB | 67 | 157 | 120 | 211 | 555 |
| Leaf | AB | 62 | 134 | 100 | 162 | 458 |
| Stem | AB | 57 | 128 | 105 | 141 | 431 |
| Ear | A | 3 | 17 | 5 | 18 | 43 |
| Leaf | A | 3 | 8 | 1 | 10 | 22 |
| Stem | A | 2 | 8 | 7 | 10 | 27 |
