# Supplementary material for: The Role of cis Regulatory Evolution in Maize Domestication
Source: PLoS Genet. 2014 Nov 6;10(11):e1004745. doi: 10.1371/journal.pgen.1004745 (PMC4222645; doi:10.1371/journal.pgen.1004745)
Supplement: Table S6 — Comparison of observed and expected numbers of genes classified as differentially expressed (DE) or not differentially expressed (NDE) by RNAseq [this study] and microarray assays [24] in groups A, B, and C in the three tissue types. (DOCX) [file pgen.1004745.s012.docx]

Table S6: Comparison of observed and expected numbers of genes classified as differentially expressed (DE) or not differentially expressed (NDE) by RNAseq [this study] and microarray assays [24] in groups A, B, and C in the three tissue types.

| **CCT Group** | **Tissue** |  | **Observed** | | **Expected** | |
| --- | --- | --- | --- | --- | --- | --- |
|  |  |  | MicroArray-NDE | MicroArray -DE | MicroArray -NDE | MicroArray -DE |
| A | Ear | RNAseq-NDE | 9587 | 184 | 9583.56 | 187.44 |
| A | Ear | RNAseq-DE | 25 | 4 | 28.44 | 0.56 |
| A | Leaf | RNAseq-NDE | 9796 | 195 | 9794.28 | 196.72 |
| A | Leaf | RNAseq-DE | 12 | 2 | 13.72 | 0.28 |
| A | Stem | RNAseq-NDE | 9804 | 198 | 9802.36 | 199.64 |
| A | Stem | RNAseq-DE | 16 | 2 | 17.64 | 0.36 |
| A | Union | RNAseq-NDE | 10104 | 204 | 10098.04 | 209.96 |
| A | Union | RNAseq-DE | 44 | 7 | 49.96 | 1.04 |
| AB | Ear | RNAseq-NDE | 9244 | 165 | 9228.50 | 180.50 |
| AB | Ear | RNAseq-DE | 368 | 23 | 383.50 | 7.50 |
| AB | Leaf | RNAseq-NDE | 9482 | 173 | 9464.89 | 190.11 |
| AB | Leaf | RNAseq-DE | 326 | 24 | 343.11 | 6.89 |
| AB | Stem | RNAseq-NDE | 9532 | 175 | 9513.25 | 193.75 |
| AB | Stem | RNAseq-DE | 288 | 25 | 306.75 | 6.25 |
| AB | Union | RNAseq-NDE | 9409 | 164 | 9378.01 | 194.99 |
| AB | Union | RNAseq-DE | 739 | 47 | 769.99 | 16.01 |
| ABC | Ear | RNAseq-NDE | 8529 | 136 | 8498.77 | 166.23 |
| ABC | Ear | RNAseq-DE | 1083 | 52 | 1113.23 | 21.77 |
| ABC | Leaf | RNAseq-NDE | 8842 | 149 | 8813.97 | 177.03 |
| ABC | Leaf | RNAseq-DE | 966 | 48 | 994.03 | 19.97 |
| ABC | Stem | RNAseq-NDE | 8835 | 154 | 8809.58 | 179.42 |
| ABC | Stem | RNAseq-DE | 985 | 46 | 1010.42 | 20.58 |
| ABC | Union | RNAseq-NDE | 7980 | 121 | 7935.99 | 165.01 |
| ABC | Union | RNAseq-DE | 2168 | 90 | 2212.01 | 45.99 |
