# Supplementary material for: The Role of cis Regulatory Evolution in Maize Domestication
Source: PLoS Genet. 2014 Nov 6;10(11):e1004745. doi: 10.1371/journal.pgen.1004745 (PMC4222645; doi:10.1371/journal.pgen.1004745)
Supplement: Table S7 — RNAseq-based regulatory categories for genes identified as differentially expressed in the microarray study [24]. (DOCX) [file pgen.1004745.s013.docx]

Table S7: RNAseq-based regulatory categories for genes identified as differentially expressed in the microarray study [24].

|  | **Ear** | **Leaf** | **Stem** |
| --- | --- | --- | --- |
| Ambiguous | 5.81% | 7.25% | 9.65% |
| *Cis* + *Trans* | 25.73% | 30.92% | 22.39% |
| *Cis* only | 26.14% | 27.48% | 30.89% |
| *Cis* x *Trans* | 6.64% | 6.11% | 8.49% |
| Compensatory | 7.05% | 7.63% | 6.56% |
| Conserved | 13.28% | 8.78% | 12.74% |
| *Trans* only | 15.35% | 11.83% | 9.27% |
| Total Genes | 241 | 262 | 259 |
