# Supplementary material for: The Role of cis Regulatory Evolution in Maize Domestication
Source: PLoS Genet. 2014 Nov 6;10(11):e1004745. doi: 10.1371/journal.pgen.1004745 (PMC4222645; doi:10.1371/journal.pgen.1004745)
Supplement: Table S8 — Fisher's exact tests for the overlap between genes associated with regions that are differentially methylated in maize and teosinte (DMRs) [27] and CCT-ABC genes from each of the three experimental tissues in our work. (DOCX) [file pgen.1004745.s014.docx]

Table S8: Fisher’s exact tests for the overlap between genes associated with regions that are differentially methylated in maize and teosinte (DMRs) [42] and CCT-ABC genes from each of the three experimental tissues in our work.

| **Overlap** | **Ear** | **Leaf** | **Stem** | **Union** |
| --- | --- | --- | --- | --- |
| Expected | 13.4664 | 11.6934 | 12.4683 | 27.493 |
| Observed | 19 | 13 | 17 | 33 |
| p-value | 0.1092 | 0.6424 | 0.1755 | 0.2358 |
