# Supplementary material for: The Role of cis Regulatory Evolution in Maize Domestication
Source: PLoS Genet. 2014 Nov 6;10(11):e1004745. doi: 10.1371/journal.pgen.1004745 (PMC4222645; doi:10.1371/journal.pgen.1004745)
Supplement: Table S9 — Number of candidate genes neighboring regions that are differentially methylated (DMRs) between maize and teosinte [27] and proportion in which the RNAseq expression data agrees with methylated status. (DOCX) [file pgen.1004745.s015.docx]

Table S9: Number of candidate genes neighboring regions that are differentially methylated (DMRs) between maize and teosinte [42] and proportion in which the RNAseq expression data agrees with methylated status.

|  | **Ear** | | **Leaf** | **Stem** |
| --- | --- | --- | --- | --- |
| Total | | 19 | 13 | 17 |
| A | | 1 | 0 | 0 |
| B | | 3 | 2 | 3 |
| C | | 15 | 11 | 14 |
| Total-agree | | 57.9% | 53.8% | 58.8% |
| A-agree | | 100% | NA | NA |
| B-agree | | 100% | 50.0% | 33.3% |
| C-agree | | 46.7% | 54.5% | 64.3% |
