# Supplementary material for: The Role of cis Regulatory Evolution in Maize Domestication
Source: PLoS Genet. 2014 Nov 6;10(11):e1004745. doi: 10.1371/journal.pgen.1004745 (PMC4222645; doi:10.1371/journal.pgen.1004745)
Supplement: Table S10 — Additive and dominant gene counts for the A, AB, and ABC cis and trans only candidate lists. Dominance cells contain the number of genes for which the maize∶teosinte allele was dominant. Fisher's exact tests (FET) interrogate whether the degree of dominance/additivity differs between the cis and trans classes. A binomial test (*) indicates significantly different maize∶teosinte count (p<0.005). (DOCX) [file pgen.1004745.s016.docx]

Table S10: Additive and dominant gene counts for the A, AB, and ABC *cis* and *trans* only candidate lists. Dominance cells contain the number of genes for which the maize:teosinte allele was dominant. Fisher’s exact tests (FET) interrogate whether the degree of dominance/additivity differs between the *cis* and *trans* classes. A binomial test (*) indicates significantly different maize:teosinte count (p < 0.005).

|  |  | Ear | |  | Leaf | |  | Stem | |
| --- | --- | --- | --- | --- | --- | --- | --- | --- | --- |
|  |  | Add | Dom |  | Add | Dom |  | Add | Dom |
| A | *Cis* only | 11 | 1:0 |  | 3 | 1:1 |  | 3 | 2:1 |
|  | *Trans* only | 13 | 19:2* |  | 5 | 4:1 |  | 2 | 0:2 |
|  |  | FET p<0.005 | |  | FET p>0.05 | |  | FET p>0.05 | |
|  |  |  |  |  |  |  |  |  |  |
|  |  |  |  |  |  |  |  |  |  |
| AB | *Cis* only | 95 | 22:18 |  | 49 | 17:19 |  | 52 | 19:20 |
|  | *Trans* only | 112 | 89:35* |  | 87 | 84:22* |  | 23 | 10:13 |
|  |  | FET p<0.005 | |  | FET p<0.05 | |  | FET p>0.05 | |
|  |  |  |  |  |  |  |  |  |  |
|  |  |  |  |  |  |  |  |  |  |
| ABC | *Cis* only | 266 | 62:65 |  | 142 | 52:59 |  | 178 | 68:71 |
|  | *Trans* only | 203 | 112:68* |  | 138 | 117:67* |  | 67 | 35:42 |
|  |  | FET p<0.005 | |  | FET p<0.005 | |  | FET p<0.05 | |
|  |  |  |  |  |  |  |  |  |  |

* = BT p-value < 0.005
