# Supplementary material for: The Role of cis Regulatory Evolution in Maize Domestication
Source: PLoS Genet. 2014 Nov 6;10(11):e1004745. doi: 10.1371/journal.pgen.1004745 (PMC4222645; doi:10.1371/journal.pgen.1004745)
Supplement: Table S11 — Degree of overlap between our CCT (AB list) genes and genes in different transcription factor families. (DOCX) [file pgen.1004745.s017.docx]

Table S11: Degree of overlap between our CCT (AB list) genes and genes in different transcription factor families.

| **Family** | **Tissue** | **Assayed Genes** | **Observed Overlap** | **Expected Overlap** | **FET p-value** |
| --- | --- | --- | --- | --- | --- |
| AP2 | Ear | 6 | 0 | 0.25 | 1.000 |
| ARF | Ear | 27 | 4 | 1.14 | 0.025 |
| ARR-B | Ear | 8 | 0 | 0.34 | 1.000 |
| B3 | Ear | 18 | 1 | 0.76 | 0.539 |
| BBR-BPC | Ear | 4 | 0 | 0.17 | 1.000 |
| BES1 | Ear | 3 | 0 | 0.13 | 1.000 |
| bHLH | Ear | 42 | 1 | 1.77 | 0.836 |
| bZIP | Ear | 51 | 0 | 2.15 | 1.000 |
| C2H2 | Ear | 28 | 2 | 1.18 | 0.331 |
| C3H | Ear | 42 | 1 | 1.77 | 0.836 |
| CAMTA | Ear | 8 | 0 | 0.34 | 1.000 |
| CO-like | Ear | 3 | 0 | 0.13 | 1.000 |
| CPP | Ear | 7 | 1 | 0.29 | 0.260 |
| DBB | Ear | 4 | 0 | 0.17 | 1.000 |
| Dof | Ear | 7 | 0 | 0.29 | 1.000 |
| E2F/DP | Ear | 10 | 0 | 0.42 | 1.000 |
| EIL | Ear | 4 | 0 | 0.17 | 1.000 |
| ERF | Ear | 17 | 0 | 0.72 | 1.000 |
| FAR1 | Ear | 15 | 2 | 0.63 | 0.129 |
| G2-like | Ear | 11 | 0 | 0.46 | 1.000 |
| GATA | Ear | 10 | 0 | 0.42 | 1.000 |
| GeBP | Ear | 14 | 0 | 0.59 | 1.000 |
| GRAS | Ear | 21 | 1 | 0.88 | 0.595 |
| GRF | Ear | 8 | 0 | 0.34 | 1.000 |
| HB-other | Ear | 14 | 0 | 0.59 | 1.000 |
| HB-PHD | Ear | 2 | 0 | 0.08 | 1.000 |
| HD-ZIP | Ear | 19 | 1 | 0.80 | 0.558 |
| HSF | Ear | 12 | 1 | 0.50 | 0.403 |
| LBD | Ear | 3 | 0 | 0.13 | 1.000 |
| LFY | Ear | 0 | 0 | 0.00 | - |
| LSD | Ear | 3 | 0 | 0.13 | 1.000 |
| M-type | Ear | 6 | 1 | 0.25 | 0.227 |
| MIKC | Ear | 23 | 2 | 0.97 | 0.252 |
| MYB | Ear | 23 | 2 | 0.97 | 0.252 |
| MYB_related | Ear | 42 | 4 | 1.77 | 0.099 |
| NAC | Ear | 25 | 0 | 1.05 | 1.000 |
| NF-X1 | Ear | 2 | 0 | 0.08 | 1.000 |
| NF-YA | Ear | 10 | 0 | 0.42 | 1.000 |
| NF-YB | Ear | 7 | 0 | 0.29 | 1.000 |
| NF-YC | Ear | 7 | 0 | 0.29 | 1.000 |
| Nin-like | Ear | 11 | 1 | 0.46 | 0.377 |
| RAV | Ear | 0 | 0 | 0.00 | - |
| S1Fa-like | Ear | 0 | 0 | 0.00 | - |
| SBP | Ear | 12 | 0 | 0.50 | 1.000 |
| SRS | Ear | 2 | 0 | 0.08 | 1.000 |
| STAT | Ear | 1 | 0 | 0.04 | 1.000 |
| TALE | Ear | 12 | 0 | 0.50 | 1.000 |
| TCP | Ear | 9 | 0 | 0.38 | 1.000 |
| Trihelix | Ear | 22 | 0 | 0.93 | 1.000 |
| VOZ | Ear | 2 | 0 | 0.08 | 1.000 |
| Whirly | Ear | 2 | 0 | 0.08 | 1.000 |
| WOX | Ear | 0 | 0 | 0.00 | - |
| WRKY | Ear | 20 | 0 | 0.84 | 1.000 |
| YABBY | Ear | 4 | 0 | 0.17 | 1.000 |
| ZF-HD | Ear | 1 | 0 | 0.04 | 1.000 |
| ALL | Ear | 649 | 24 | 27.30 | 0.774 |
| AP2 | Leaf | 8 | 0 | 0.28 | 1.000 |
| ARF | Leaf | 27 | 0 | 0.94 | 1.000 |
| ARR-B | Leaf | 8 | 0 | 0.28 | 1.000 |
| B3 | Leaf | 16 | 1 | 0.56 | 0.433 |
| BBR-BPC | Leaf | 4 | 0 | 0.14 | 1.000 |
| BES1 | Leaf | 3 | 0 | 0.10 | 1.000 |
| bHLH | Leaf | 41 | 1 | 1.43 | 0.766 |
| bZIP | Leaf | 42 | 0 | 1.46 | 1.000 |
| C2H2 | Leaf | 29 | 2 | 1.01 | 0.268 |
| C3H | Leaf | 41 | 0 | 1.43 | 1.000 |
| CAMTA | Leaf | 8 | 0 | 0.28 | 1.000 |
| CO-like | Leaf | 5 | 0 | 0.17 | 1.000 |
| CPP | Leaf | 7 | 0 | 0.24 | 1.000 |
| DBB | Leaf | 6 | 0 | 0.21 | 1.000 |
| Dof | Leaf | 9 | 0 | 0.31 | 1.000 |
| E2F/DP | Leaf | 10 | 0 | 0.35 | 1.000 |
| EIL | Leaf | 4 | 0 | 0.14 | 1.000 |
| ERF | Leaf | 15 | 0 | 0.52 | 1.000 |
| FAR1 | Leaf | 14 | 0 | 0.49 | 1.000 |
| G2-like | Leaf | 16 | 0 | 0.56 | 1.000 |
| GATA | Leaf | 14 | 0 | 0.49 | 1.000 |
| GeBP | Leaf | 14 | 0 | 0.49 | 1.000 |
| GRAS | Leaf | 20 | 1 | 0.70 | 0.508 |
| GRF | Leaf | 6 | 0 | 0.21 | 1.000 |
| HB-other | Leaf | 15 | 2 | 0.52 | 0.094 |
| HB-PHD | Leaf | 2 | 0 | 0.07 | 1.000 |
| HD-ZIP | Leaf | 16 | 0 | 0.56 | 1.000 |
| HSF | Leaf | 10 | 0 | 0.35 | 1.000 |
| LBD | Leaf | 2 | 1 | 0.07 | 0.068 |
| LFY | Leaf | 0 | 0 | 0.00 | - |
| LSD | Leaf | 3 | 0 | 0.10 | 1.000 |
| M-type | Leaf | 3 | 0 | 0.10 | 1.000 |
| MIKC | Leaf | 9 | 2 | 0.31 | 0.037 |
| MYB | Leaf | 31 | 2 | 1.08 | 0.294 |
| MYB_related | Leaf | 44 | 1 | 1.53 | 0.790 |
| NAC | Leaf | 28 | 2 | 0.97 | 0.255 |
| NF-X1 | Leaf | 2 | 0 | 0.07 | 1.000 |
| NF-YA | Leaf | 9 | 0 | 0.31 | 1.000 |
| NF-YB | Leaf | 5 | 0 | 0.17 | 1.000 |
| NF-YC | Leaf | 8 | 0 | 0.28 | 1.000 |
| Nin-like | Leaf | 10 | 0 | 0.35 | 1.000 |
| RAV | Leaf | 0 | 0 | 0.00 | - |
| S1Fa-like | Leaf | 0 | 0 | 0.00 | - |
| SBP | Leaf | 12 | 0 | 0.42 | 1.000 |
| SRS | Leaf | 0 | 0 | 0.00 | - |
| STAT | Leaf | 1 | 0 | 0.03 | 1.000 |
| TALE | Leaf | 12 | 0 | 0.42 | 1.000 |
| TCP | Leaf | 8 | 0 | 0.28 | 1.000 |
| Trihelix | Leaf | 22 | 1 | 0.77 | 0.541 |
| VOZ | Leaf | 2 | 0 | 0.07 | 1.000 |
| Whirly | Leaf | 2 | 0 | 0.07 | 1.000 |
| WOX | Leaf | 0 | 0 | 0.00 | - |
| WRKY | Leaf | 16 | 0 | 0.56 | 1.000 |
| YABBY | Leaf | 4 | 0 | 0.14 | 1.000 |
| ZF-HD | Leaf | 1 | 0 | 0.03 | 1.000 |
| ALL | Leaf | 628 | 16 | 21.85 | 0.927 |
| AP2 | Stem | 8 | 0 | 0.26 | 1.000 |
| ARF | Stem | 27 | 3 | 0.87 | 0.056 |
| ARR-B | Stem | 8 | 0 | 0.26 | 1.000 |
| B3 | Stem | 14 | 0 | 0.45 | 1.000 |
| BBR-BPC | Stem | 4 | 0 | 0.13 | 1.000 |
| BES1 | Stem | 3 | 0 | 0.10 | 1.000 |
| bHLH | Stem | 50 | 2 | 1.62 | 0.485 |
| bZIP | Stem | 47 | 1 | 1.52 | 0.788 |
| C2H2 | Stem | 28 | 2 | 0.91 | 0.229 |
| C3H | Stem | 41 | 1 | 1.33 | 0.741 |
| CAMTA | Stem | 8 | 0 | 0.26 | 1.000 |
| CO-like | Stem | 4 | 0 | 0.13 | 1.000 |
| CPP | Stem | 7 | 0 | 0.23 | 1.000 |
| DBB | Stem | 6 | 0 | 0.19 | 1.000 |
| Dof | Stem | 8 | 0 | 0.26 | 1.000 |
| E2F/DP | Stem | 10 | 0 | 0.32 | 1.000 |
| EIL | Stem | 4 | 1 | 0.13 | 0.123 |
| ERF | Stem | 16 | 0 | 0.52 | 1.000 |
| FAR1 | Stem | 15 | 0 | 0.49 | 1.000 |
| G2-like | Stem | 14 | 0 | 0.45 | 1.000 |
| GATA | Stem | 12 | 0 | 0.39 | 1.000 |
| GeBP | Stem | 13 | 0 | 0.42 | 1.000 |
| GRAS | Stem | 20 | 0 | 0.65 | 1.000 |
| GRF | Stem | 7 | 0 | 0.23 | 1.000 |
| HB-other | Stem | 15 | 2 | 0.49 | 0.083 |
| HB-PHD | Stem | 2 | 0 | 0.06 | 1.000 |
| HD-ZIP | Stem | 17 | 1 | 0.55 | 0.429 |
| HSF | Stem | 14 | 0 | 0.45 | 1.000 |
| LBD | Stem | 2 | 0 | 0.06 | 1.000 |
| LFY | Stem | 0 | 0 | 0.00 | - |
| LSD | Stem | 3 | 0 | 0.10 | 1.000 |
| M-type | Stem | 4 | 1 | 0.13 | 0.123 |
| MIKC | Stem | 10 | 2 | 0.32 | 0.040 |
| MYB | Stem | 23 | 2 | 0.75 | 0.170 |
| MYB_related | Stem | 42 | 1 | 1.36 | 0.750 |
| NAC | Stem | 29 | 0 | 0.94 | 1.000 |
| NF-X1 | Stem | 2 | 0 | 0.06 | 1.000 |
| NF-YA | Stem | 10 | 1 | 0.32 | 0.281 |
| NF-YB | Stem | 6 | 0 | 0.19 | 1.000 |
| NF-YC | Stem | 7 | 0 | 0.23 | 1.000 |
| Nin-like | Stem | 11 | 0 | 0.36 | 1.000 |
| RAV | Stem | 0 | 0 | 0.00 | - |
| S1Fa-like | Stem | 0 | 0 | 0.00 | - |
| SBP | Stem | 11 | 0 | 0.36 | 1.000 |
| SRS | Stem | 2 | 0 | 0.06 | 1.000 |
| STAT | Stem | 1 | 0 | 0.03 | 1.000 |
| TALE | Stem | 13 | 0 | 0.42 | 1.000 |
| TCP | Stem | 6 | 1 | 0.19 | 0.179 |
| Trihelix | Stem | 23 | 0 | 0.75 | 1.000 |
| VOZ | Stem | 2 | 0 | 0.06 | 1.000 |
| Whirly | Stem | 2 | 0 | 0.06 | 1.000 |
| WOX | Stem | 0 | 0 | 0.00 | - |
| WRKY | Stem | 19 | 0 | 0.62 | 1.000 |
| YABBY | Stem | 4 | 0 | 0.13 | 1.000 |
| ZF-HD | Stem | 0 | 0 | 0.00 | - |
| ALL | Stem | 640 | 20 | 20.73 | 0.599 |
| AP2 | Union | 10 | 0 | 0.77 | 1.000 |
| ARF | Union | 27 | 6 | 2.07 | 0.015 |
| ARR-B | Union | 8 | 0 | 0.61 | 1.000 |
| B3 | Union | 18 | 2 | 1.38 | 0.407 |
| BBR-BPC | Union | 4 | 0 | 0.31 | 1.000 |
| BES1 | Union | 3 | 0 | 0.23 | 1.000 |
| bHLH | Union | 53 | 4 | 4.07 | 0.588 |
| bZIP | Union | 52 | 1 | 3.99 | 0.984 |
| C2H2 | Union | 31 | 4 | 2.38 | 0.211 |
| C3H | Union | 42 | 2 | 3.22 | 0.843 |
| CAMTA | Union | 8 | 0 | 0.61 | 1.000 |
| CO-like | Union | 5 | 0 | 0.38 | 1.000 |
| CPP | Union | 7 | 1 | 0.54 | 0.428 |
| DBB | Union | 6 | 0 | 0.46 | 1.000 |
| Dof | Union | 9 | 0 | 0.69 | 1.000 |
| E2F/DP | Union | 10 | 0 | 0.77 | 1.000 |
| EIL | Union | 4 | 1 | 0.31 | 0.273 |
| ERF | Union | 18 | 0 | 1.38 | 1.000 |
| FAR1 | Union | 15 | 2 | 1.15 | 0.322 |
| G2-like | Union | 18 | 0 | 1.38 | 1.000 |
| GATA | Union | 15 | 0 | 1.15 | 1.000 |
| GeBP | Union | 15 | 0 | 1.15 | 1.000 |
| GRAS | Union | 23 | 2 | 1.77 | 0.536 |
| GRF | Union | 8 | 0 | 0.61 | 1.000 |
| HB-other | Union | 15 | 3 | 1.15 | 0.103 |
| HB-PHD | Union | 2 | 0 | 0.15 | 1.000 |
| HD-ZIP | Union | 20 | 2 | 1.53 | 0.461 |
| HSF | Union | 14 | 1 | 1.07 | 0.673 |
| LBD | Union | 3 | 1 | 0.23 | 0.213 |
| LFY | Union | 0 | 0 | 0.00 | - |
| LSD | Union | 3 | 0 | 0.23 | 1.000 |
| M-type | Union | 7 | 2 | 0.54 | 0.095 |
| MIKC | Union | 25 | 5 | 1.92 | 0.039 |
| MYB | Union | 32 | 3 | 2.46 | 0.450 |
| MYB_related | Union | 48 | 4 | 3.68 | 0.508 |
| NAC | Union | 35 | 2 | 2.69 | 0.761 |
| NF-X1 | Union | 2 | 0 | 0.15 | 1.000 |
| NF-YA | Union | 10 | 1 | 0.77 | 0.550 |
| NF-YB | Union | 7 | 0 | 0.54 | 1.000 |
| NF-YC | Union | 8 | 0 | 0.61 | 1.000 |
| Nin-like | Union | 11 | 1 | 0.84 | 0.585 |
| RAV | Union | 0 | 0 | 0.00 | - |
| S1Fa-like | Union | 0 | 0 | 0.00 | - |
| SBP | Union | 13 | 0 | 1.00 | 1.000 |
| SRS | Union | 2 | 0 | 0.15 | 1.000 |
| STAT | Union | 1 | 0 | 0.08 | 1.000 |
| TALE | Union | 14 | 0 | 1.07 | 1.000 |
| TCP | Union | 9 | 1 | 0.69 | 0.513 |
| Trihelix | Union | 24 | 1 | 1.84 | 0.853 |
| VOZ | Union | 2 | 0 | 0.15 | 1.000 |
| Whirly | Union | 2 | 0 | 0.15 | 1.000 |
| WOX | Union | 0 | 0 | 0.00 | - |
| WRKY | Union | 23 | 0 | 1.77 | 1.000 |
| YABBY | Union | 4 | 0 | 0.31 | 1.000 |
| ZF-HD | Union | 1 | 0 | 0.08 | 1.000 |
| ALL | Union | 725 | 50 | 55.64 | 0.809 |
