# Supplementary material for: The Role of cis Regulatory Evolution in Maize Domestication
Source: PLoS Genet. 2014 Nov 6;10(11):e1004745. doi: 10.1371/journal.pgen.1004745 (PMC4222645; doi:10.1371/journal.pgen.1004745)
Supplement: Table S12 — Degree of overlap between CCT (AB list) differentially expressed genes and genes in the 1.5 support intervals for QTL from a previous study. (DOCX) [file pgen.1004745.s018.docx]

Table S12: Degree of overlap between CCT (AB list) differentially expressed genes and genes in the 1.5 support intervals for QTL (containing at least 20 genes) from a previous study.

| **Trait** | **Tissue** | **Assayed Genes** | **Observed Overlap** | **Expected Overlap** | **FET p-value** |
| --- | --- | --- | --- | --- | --- |
| BARE | Ear | 0 | 0 | 0.00 | 1.00 |
| DIAM | Ear | 29 | 4 | 1.22 | 0.03 |
| DIS | Ear | 4 | 1 | 0.17 | 0.16 |
| DTP | Ear | 10 | 1 | 0.42 | 0.35 |
| GLCO | Ear | 3 | 0 | 0.13 | 1.00 |
| GLU | Ear | 0 | 0 | 0.00 | 1.00 |
| KRN | Ear | 15 | 2 | 0.63 | 0.13 |
| KW | Ear | 17 | 1 | 0.72 | 0.52 |
| LEN | Ear | 4 | 1 | 0.17 | 0.16 |
| PROL | Ear | 5 | 0 | 0.21 | 1.00 |
| STAM | Ear | 10 | 1 | 0.42 | 0.35 |
| BARE | Leaf | 0 | 0 | 0.00 | 1.00 |
| DIAM | Leaf | 28 | 0 | 0.97 | 1.00 |
| DIS | Leaf | 4 | 2 | 0.14 | 0.01 |
| DTP | Leaf | 9 | 0 | 0.31 | 1.00 |
| GLCO | Leaf | 3 | 0 | 0.10 | 1.00 |
| GLU | Leaf | 0 | 0 | 0.00 | 1.00 |
| KRN | Leaf | 13 | 0 | 0.45 | 1.00 |
| KW | Leaf | 17 | 2 | 0.59 | 0.12 |
| LEN | Leaf | 4 | 0 | 0.14 | 1.00 |
| PROL | Leaf | 5 | 0 | 0.17 | 1.00 |
| STAM | Leaf | 9 | 1 | 0.31 | 0.27 |
| BARE | Stem | 0 | 0 | 0.00 | 1.00 |
| DIAM | Stem | 28 | 1 | 0.91 | 0.60 |
| DIS | Stem | 4 | 0 | 0.13 | 1.00 |
| DTP | Stem | 10 | 0 | 0.32 | 1.00 |
| GLCO | Stem | 3 | 0 | 0.10 | 1.00 |
| GLU | Stem | 0 | 0 | 0.00 | 1.00 |
| KRN | Stem | 14 | 1 | 0.45 | 0.37 |
| KW | Stem | 18 | 3 | 0.58 | 0.02 |
| LEN | Stem | 4 | 0 | 0.13 | 1.00 |
| PROL | Stem | 5 | 0 | 0.16 | 1.00 |
| STAM | Stem | 10 | 0 | 0.32 | 1.00 |
