# Supplementary material for: The Role of cis Regulatory Evolution in Maize Domestication
Source: PLoS Genet. 2014 Nov 6;10(11):e1004745. doi: 10.1371/journal.pgen.1004745 (PMC4222645; doi:10.1371/journal.pgen.1004745)
Supplement: Table S13 — Degree overlap between our CCT and trans only differentially expressed genes and genes in metabolic pathways defined in KEGG. (DOCX) [file pgen.1004745.s019.docx]

Table S13: Degree overlap between our CCT and *trans* only differentially expressed genes and genes in metabolic pathways defined in KEGG.

| Pathway | Tissue | Group | Genes in Pathway | Assayed genes | Observed Overlap | Expected Overlap | p-value |
| --- | --- | --- | --- | --- | --- | --- | --- |
| Alpha-linoleic Acid Metabolism | Ear | CCT-A | 26 | 14 | 0 | 0.046 | 1.000 |
| Arachidonic Acid Metabolism | Ear | CCT-A | 10 | 7 | 0 | 0.023 | 1.000 |
| Biosynthesis of Unsaturated Fatty Acids | Ear | CCT-A | 33 | 16 | 0 | 0.052 | 1.000 |
| Cutin, Suberine, and Wax Biosynthesis | Ear | CCT-A | 10 | 5 | 0 | 0.016 | 1.000 |
| Ether Lipid Metabolism | Ear | CCT-A | 11 | 7 | 0 | 0.023 | 1.000 |
| Fatty Acid Biosynthesis | Ear | CCT-A | 32 | 19 | 0 | 0.062 | 1.000 |
| Fatty Acid Degradation | Ear | CCT-A | 34 | 27 | 0 | 0.088 | 1.000 |
| Fatty Acid Elongation | Ear | CCT-A | 16 | 8 | 0 | 0.026 | 1.000 |
| Glycerolipid Metabolism | Ear | CCT-A | 46 | 31 | 0 | 0.101 | 1.000 |
| Glycerophospholipid Metabolism | Ear | CCT-A | 64 | 45 | 0 | 0.147 | 1.000 |
| Linoleic Acid Metabolism | Ear | CCT-A | 12 | 5 | 0 | 0.016 | 1.000 |
| Sphingolipid Metabolism | Ear | CCT-A | 21 | 13 | 0 | 0.042 | 1.000 |
| Starch and sucrose metabolism | Ear | CCT-A | 98 | 59 | 0 | 0.192 | 1.000 |
| Steroid Biosynthesis | Ear | CCT-A | 25 | 15 | 0 | 0.049 | 1.000 |
| Synthesis/Degradation of Ketone Bodies | Ear | CCT-A | 8 | 8 | 0 | 0.026 | 1.000 |
| ALL | Ear | CCT-A | 353 | 223 | 0 | 0.727 | 1.000 |
| Alpha-linoleic Acid Metabolism | Ear | CCT-AB | 26 | 14 | 1 | 0.589 | 0.452 |
| Arachidonic Acid Metabolism | Ear | CCT-AB | 10 | 7 | 0 | 0.294 | 1.000 |
| Biosynthesis of Unsaturated Fatty Acids | Ear | CCT-AB | 33 | 16 | 0 | 0.673 | 1.000 |
| Cutin, Suberine, and Wax Biosynthesis | Ear | CCT-AB | 10 | 5 | 0 | 0.210 | 1.000 |
| Ether Lipid Metabolism | Ear | CCT-AB | 11 | 7 | 0 | 0.294 | 1.000 |
| Fatty Acid Biosynthesis | Ear | CCT-AB | 32 | 19 | 2 | 0.799 | 0.189 |
| Fatty Acid Degradation | Ear | CCT-AB | 34 | 27 | 1 | 1.136 | 0.687 |
| Fatty Acid Elongation | Ear | CCT-AB | 16 | 8 | 0 | 0.337 | 1.000 |
| Glycerolipid Metabolism | Ear | CCT-AB | 46 | 31 | 0 | 1.304 | 1.000 |
| Glycerophospholipid Metabolism | Ear | CCT-AB | 64 | 45 | 0 | 1.893 | 1.000 |
| Linoleic Acid Metabolism | Ear | CCT-AB | 12 | 5 | 1 | 0.210 | 0.193 |
| Sphingolipid Metabolism | Ear | CCT-AB | 21 | 13 | 0 | 0.547 | 1.000 |
| Starch and sucrose metabolism | Ear | CCT-AB | 98 | 59 | 3 | 2.482 | 0.454 |
| Steroid Biosynthesis | Ear | CCT-AB | 25 | 15 | 1 | 0.631 | 0.475 |
| Synthesis/Degradation of Ketone Bodies | Ear | CCT-AB | 8 | 8 | 1 | 0.337 | 0.291 |
| ALL | Ear | CCT-AB | 353 | 223 | 8 | 9.380 | 0.726 |
| Alpha-linoleic Acid Metabolism | Ear | CCT-ABC | 26 | 14 | 5 | 1.639 | 0.018 |
| Arachidonic Acid Metabolism | Ear | CCT-ABC | 10 | 7 | 1 | 0.820 | 0.582 |
| Biosynthesis of Unsaturated Fatty Acids | Ear | CCT-ABC | 33 | 16 | 2 | 1.874 | 0.575 |
| Cutin, Suberine, and Wax Biosynthesis | Ear | CCT-ABC | 10 | 5 | 0 | 0.585 | 1.000 |
| Ether Lipid Metabolism | Ear | CCT-ABC | 11 | 7 | 1 | 0.820 | 0.582 |
| Fatty Acid Biosynthesis | Ear | CCT-ABC | 32 | 19 | 3 | 2.225 | 0.388 |
| Fatty Acid Degradation | Ear | CCT-ABC | 34 | 27 | 5 | 3.162 | 0.203 |
| Fatty Acid Elongation | Ear | CCT-ABC | 16 | 8 | 0 | 0.937 | 1.000 |
| Glycerolipid Metabolism | Ear | CCT-ABC | 46 | 31 | 3 | 3.630 | 0.721 |
| Glycerophospholipid Metabolism | Ear | CCT-ABC | 64 | 45 | 5 | 5.269 | 0.619 |
| Linoleic Acid Metabolism | Ear | CCT-ABC | 12 | 5 | 1 | 0.585 | 0.464 |
| Sphingolipid Metabolism | Ear | CCT-ABC | 21 | 13 | 0 | 1.522 | 1.000 |
| Starch and sucrose metabolism | Ear | CCT-ABC | 98 | 59 | 7 | 6.909 | 0.545 |
| Steroid Biosynthesis | Ear | CCT-ABC | 25 | 15 | 2 | 1.756 | 0.539 |
| Synthesis/Degradation of Ketone Bodies | Ear | CCT-ABC | 8 | 8 | 1 | 0.937 | 0.631 |
| ALL | Ear | CCT-ABC | 353 | 223 | 28 | 26.113 | 0.376 |
| Alpha-linoleic Acid Metabolism | Ear | trans-A | 26 | 14 | 1 | 0.062 | 0.060 |
| Arachidonic Acid Metabolism | Ear | trans-A | 10 | 7 | 0 | 0.031 | 1.000 |
| Biosynthesis of Unsaturated Fatty Acids | Ear | trans-A | 33 | 16 | 0 | 0.070 | 1.000 |
| Cutin, Suberine, and Wax Biosynthesis | Ear | trans-A | 10 | 5 | 0 | 0.022 | 1.000 |
| Ether Lipid Metabolism | Ear | trans-A | 11 | 7 | 1 | 0.031 | 0.030 |
| Fatty Acid Biosynthesis | Ear | trans-A | 32 | 19 | 0 | 0.084 | 1.000 |
| Fatty Acid Degradation | Ear | trans-A | 34 | 27 | 0 | 0.119 | 1.000 |
| Fatty Acid Elongation | Ear | trans-A | 16 | 8 | 0 | 0.035 | 1.000 |
| Glycerolipid Metabolism | Ear | trans-A | 46 | 31 | 0 | 0.136 | 1.000 |
| Glycerophospholipid Metabolism | Ear | trans-A | 64 | 45 | 2 | 0.198 | 0.017 |
| Linoleic Acid Metabolism | Ear | trans-A | 12 | 5 | 1 | 0.022 | 0.022 |
| Sphingolipid Metabolism | Ear | trans-A | 21 | 13 | 0 | 0.057 | 1.000 |
| Starch and sucrose metabolism | Ear | trans-A | 98 | 59 | 2 | 0.259 | 0.028 |
| Steroid Biosynthesis | Ear | trans-A | 25 | 15 | 0 | 0.066 | 1.000 |
| Synthesis/Degradation of Ketone Bodies | Ear | trans-A | 8 | 8 | 0 | 0.035 | 1.000 |
| ALL | Ear | trans-A | 353 | 223 | 5 | 0.980 | 0.003 |
| Alpha-linoleic Acid Metabolism | Ear | trans-AB | 26 | 14 | 2 | 0.506 | 0.089 |
| Arachidonic Acid Metabolism | Ear | trans-AB | 10 | 7 | 0 | 0.253 | 1.000 |
| Biosynthesis of Unsaturated Fatty Acids | Ear | trans-AB | 33 | 16 | 1 | 0.578 | 0.445 |
| Cutin, Suberine, and Wax Biosynthesis | Ear | trans-AB | 10 | 5 | 2 | 0.181 | 0.012 |
| Ether Lipid Metabolism | Ear | trans-AB | 11 | 7 | 1 | 0.253 | 0.227 |
| Fatty Acid Biosynthesis | Ear | trans-AB | 32 | 19 | 1 | 0.687 | 0.503 |
| Fatty Acid Degradation | Ear | trans-AB | 34 | 27 | 2 | 0.976 | 0.255 |
| Fatty Acid Elongation | Ear | trans-AB | 16 | 8 | 0 | 0.289 | 1.000 |
| Glycerolipid Metabolism | Ear | trans-AB | 46 | 31 | 4 | 1.121 | 0.025 |
| Glycerophospholipid Metabolism | Ear | trans-AB | 64 | 45 | 5 | 1.627 | 0.023 |
| Linoleic Acid Metabolism | Ear | trans-AB | 12 | 5 | 1 | 0.181 | 0.168 |
| Sphingolipid Metabolism | Ear | trans-AB | 21 | 13 | 0 | 0.470 | 1.000 |
| Starch and sucrose metabolism | Ear | trans-AB | 98 | 59 | 3 | 2.133 | 0.360 |
| Steroid Biosynthesis | Ear | trans-AB | 25 | 15 | 0 | 0.542 | 1.000 |
| Synthesis/Degradation of Ketone Bodies | Ear | trans-AB | 8 | 8 | 0 | 0.289 | 1.000 |
| ALL | Ear | trans-AB | 353 | 223 | 15 | 8.062 | 0.016 |
| Alpha-linoleic Acid Metabolism | Ear | trans-ABC | 26 | 14 | 2 | 1.213 | 0.345 |
| Arachidonic Acid Metabolism | Ear | trans-ABC | 10 | 7 | 0 | 0.606 | 1.000 |
| Biosynthesis of Unsaturated Fatty Acids | Ear | trans-ABC | 33 | 16 | 1 | 1.386 | 0.766 |
| Cutin, Suberine, and Wax Biosynthesis | Ear | trans-ABC | 10 | 5 | 2 | 0.433 | 0.063 |
| Ether Lipid Metabolism | Ear | trans-ABC | 11 | 7 | 2 | 0.606 | 0.118 |
| Fatty Acid Biosynthesis | Ear | trans-ABC | 32 | 19 | 1 | 1.646 | 0.821 |
| Fatty Acid Degradation | Ear | trans-ABC | 34 | 27 | 3 | 2.339 | 0.418 |
| Fatty Acid Elongation | Ear | trans-ABC | 16 | 8 | 1 | 0.693 | 0.516 |
| Glycerolipid Metabolism | Ear | trans-ABC | 46 | 31 | 6 | 2.686 | 0.047 |
| Glycerophospholipid Metabolism | Ear | trans-ABC | 64 | 45 | 7 | 3.898 | 0.090 |
| Linoleic Acid Metabolism | Ear | trans-ABC | 12 | 5 | 1 | 0.433 | 0.364 |
| Sphingolipid Metabolism | Ear | trans-ABC | 21 | 13 | 0 | 1.126 | 1.000 |
| Starch and sucrose metabolism | Ear | trans-ABC | 98 | 59 | 5 | 5.111 | 0.588 |
| Steroid Biosynthesis | Ear | trans-ABC | 25 | 15 | 2 | 1.299 | 0.378 |
| Synthesis/Degradation of Ketone Bodies | Ear | trans-ABC | 8 | 8 | 0 | 0.693 | 1.000 |
| ALL | Ear | trans-ABC | 353 | 223 | 23 | 19.319 | 0.218 |
| Alpha-linoleic Acid Metabolism | Leaf | CCT-A | 26 | 13 | 0 | 0.022 | 1.000 |
| Arachidonic Acid Metabolism | Leaf | CCT-A | 10 | 7 | 0 | 0.012 | 1.000 |
| Biosynthesis of Unsaturated Fatty Acids | Leaf | CCT-A | 33 | 19 | 0 | 0.032 | 1.000 |
| Cutin, Suberine, and Wax Biosynthesis | Leaf | CCT-A | 10 | 6 | 0 | 0.010 | 1.000 |
| Ether Lipid Metabolism | Leaf | CCT-A | 11 | 7 | 0 | 0.012 | 1.000 |
| Fatty Acid Biosynthesis | Leaf | CCT-A | 32 | 19 | 0 | 0.032 | 1.000 |
| Fatty Acid Degradation | Leaf | CCT-A | 34 | 30 | 0 | 0.050 | 1.000 |
| Fatty Acid Elongation | Leaf | CCT-A | 16 | 9 | 0 | 0.015 | 1.000 |
| Glycerolipid Metabolism | Leaf | CCT-A | 46 | 34 | 0 | 0.057 | 1.000 |
| Glycerophospholipid Metabolism | Leaf | CCT-A | 64 | 47 | 0 | 0.079 | 1.000 |
| Linoleic Acid Metabolism | Leaf | CCT-A | 12 | 5 | 0 | 0.008 | 1.000 |
| Sphingolipid Metabolism | Leaf | CCT-A | 21 | 14 | 0 | 0.023 | 1.000 |
| Starch and sucrose metabolism | Leaf | CCT-A | 98 | 62 | 0 | 0.104 | 1.000 |
| Steroid Biosynthesis | Leaf | CCT-A | 25 | 15 | 0 | 0.025 | 1.000 |
| Synthesis/Degradation of Ketone Bodies | Leaf | CCT-A | 8 | 8 | 0 | 0.013 | 1.000 |
| ALL | Leaf | CCT-A | 353 | 236 | 0 | 0.394 | 1.000 |
| Alpha-linoleic Acid Metabolism | Leaf | CCT-AB | 26 | 13 | 0 | 0.452 | 1.000 |
| Arachidonic Acid Metabolism | Leaf | CCT-AB | 10 | 7 | 0 | 0.244 | 1.000 |
| Biosynthesis of Unsaturated Fatty Acids | Leaf | CCT-AB | 33 | 19 | 2 | 0.661 | 0.140 |
| Cutin, Suberine, and Wax Biosynthesis | Leaf | CCT-AB | 10 | 6 | 0 | 0.209 | 1.000 |
| Ether Lipid Metabolism | Leaf | CCT-AB | 11 | 7 | 1 | 0.244 | 0.220 |
| Fatty Acid Biosynthesis | Leaf | CCT-AB | 32 | 19 | 0 | 0.661 | 1.000 |
| Fatty Acid Degradation | Leaf | CCT-AB | 34 | 30 | 2 | 1.044 | 0.281 |
| Fatty Acid Elongation | Leaf | CCT-AB | 16 | 9 | 0 | 0.313 | 1.000 |
| Glycerolipid Metabolism | Leaf | CCT-AB | 46 | 34 | 1 | 1.183 | 0.700 |
| Glycerophospholipid Metabolism | Leaf | CCT-AB | 64 | 47 | 2 | 1.635 | 0.490 |
| Linoleic Acid Metabolism | Leaf | CCT-AB | 12 | 5 | 0 | 0.174 | 1.000 |
| Sphingolipid Metabolism | Leaf | CCT-AB | 21 | 14 | 1 | 0.487 | 0.391 |
| Starch and sucrose metabolism | Leaf | CCT-AB | 98 | 62 | 1 | 2.157 | 0.889 |
| Steroid Biosynthesis | Leaf | CCT-AB | 25 | 15 | 0 | 0.522 | 1.000 |
| Synthesis/Degradation of Ketone Bodies | Leaf | CCT-AB | 8 | 8 | 1 | 0.278 | 0.247 |
| ALL | Leaf | CCT-AB | 353 | 236 | 8 | 8.211 | 0.580 |
| Alpha-linoleic Acid Metabolism | Leaf | CCT-ABC | 26 | 13 | 2 | 1.310 | 0.383 |
| Arachidonic Acid Metabolism | Leaf | CCT-ABC | 10 | 7 | 0 | 0.706 | 1.000 |
| Biosynthesis of Unsaturated Fatty Acids | Leaf | CCT-ABC | 33 | 19 | 5 | 1.915 | 0.036 |
| Cutin, Suberine, and Wax Biosynthesis | Leaf | CCT-ABC | 10 | 6 | 2 | 0.605 | 0.116 |
| Ether Lipid Metabolism | Leaf | CCT-ABC | 11 | 7 | 1 | 0.706 | 0.525 |
| Fatty Acid Biosynthesis | Leaf | CCT-ABC | 32 | 19 | 0 | 1.915 | 1.000 |
| Fatty Acid Degradation | Leaf | CCT-ABC | 34 | 30 | 8 | 3.024 | 0.008 |
| Fatty Acid Elongation | Leaf | CCT-ABC | 16 | 9 | 1 | 0.907 | 0.616 |
| Glycerolipid Metabolism | Leaf | CCT-ABC | 46 | 34 | 5 | 3.427 | 0.255 |
| Glycerophospholipid Metabolism | Leaf | CCT-ABC | 64 | 47 | 4 | 4.738 | 0.711 |
| Linoleic Acid Metabolism | Leaf | CCT-ABC | 12 | 5 | 0 | 0.504 | 1.000 |
| Sphingolipid Metabolism | Leaf | CCT-ABC | 21 | 14 | 2 | 1.411 | 0.420 |
| Starch and sucrose metabolism | Leaf | CCT-ABC | 98 | 62 | 6 | 6.250 | 0.604 |
| Steroid Biosynthesis | Leaf | CCT-ABC | 25 | 15 | 0 | 1.512 | 1.000 |
| Synthesis/Degradation of Ketone Bodies | Leaf | CCT-ABC | 8 | 8 | 1 | 0.806 | 0.573 |
| ALL | Leaf | CCT-ABC | 353 | 236 | 27 | 23.790 | 0.271 |
| Alpha-linoleic Acid Metabolism | Leaf | trans-A | 26 | 13 | 0 | 0.028 | 1.000 |
| Arachidonic Acid Metabolism | Leaf | trans-A | 10 | 7 | 0 | 0.015 | 1.000 |
| Biosynthesis of Unsaturated Fatty Acids | Leaf | trans-A | 33 | 19 | 0 | 0.040 | 1.000 |
| Cutin, Suberine, and Wax Biosynthesis | Leaf | trans-A | 10 | 6 | 0 | 0.013 | 1.000 |
| Ether Lipid Metabolism | Leaf | trans-A | 11 | 7 | 0 | 0.015 | 1.000 |
| Fatty Acid Biosynthesis | Leaf | trans-A | 32 | 19 | 0 | 0.040 | 1.000 |
| Fatty Acid Degradation | Leaf | trans-A | 34 | 30 | 0 | 0.064 | 1.000 |
| Fatty Acid Elongation | Leaf | trans-A | 16 | 9 | 0 | 0.019 | 1.000 |
| Glycerolipid Metabolism | Leaf | trans-A | 46 | 34 | 0 | 0.072 | 1.000 |
| Glycerophospholipid Metabolism | Leaf | trans-A | 64 | 47 | 0 | 0.100 | 1.000 |
| Linoleic Acid Metabolism | Leaf | trans-A | 12 | 5 | 0 | 0.011 | 1.000 |
| Sphingolipid Metabolism | Leaf | trans-A | 21 | 14 | 0 | 0.030 | 1.000 |
| Starch and sucrose metabolism | Leaf | trans-A | 98 | 62 | 0 | 0.132 | 1.000 |
| Steroid Biosynthesis | Leaf | trans-A | 25 | 15 | 0 | 0.032 | 1.000 |
| Synthesis/Degradation of Ketone Bodies | Leaf | trans-A | 8 | 8 | 0 | 0.017 | 1.000 |
| ALL | Leaf | trans-A | 353 | 236 | 0 | 0.502 | 1.000 |
| Alpha-linoleic Acid Metabolism | Leaf | trans-AB | 26 | 13 | 1 | 0.477 | 0.385 |
| Arachidonic Acid Metabolism | Leaf | trans-AB | 10 | 7 | 0 | 0.257 | 1.000 |
| Biosynthesis of Unsaturated Fatty Acids | Leaf | trans-AB | 33 | 19 | 0 | 0.697 | 1.000 |
| Cutin, Suberine, and Wax Biosynthesis | Leaf | trans-AB | 10 | 6 | 0 | 0.220 | 1.000 |
| Ether Lipid Metabolism | Leaf | trans-AB | 11 | 7 | 0 | 0.257 | 1.000 |
| Fatty Acid Biosynthesis | Leaf | trans-AB | 32 | 19 | 1 | 0.697 | 0.509 |
| Fatty Acid Degradation | Leaf | trans-AB | 34 | 30 | 0 | 1.101 | 1.000 |
| Fatty Acid Elongation | Leaf | trans-AB | 16 | 9 | 0 | 0.330 | 1.000 |
| Glycerolipid Metabolism | Leaf | trans-AB | 46 | 34 | 1 | 1.247 | 0.720 |
| Glycerophospholipid Metabolism | Leaf | trans-AB | 64 | 47 | 0 | 1.724 | 1.000 |
| Linoleic Acid Metabolism | Leaf | trans-AB | 12 | 5 | 1 | 0.183 | 0.171 |
| Sphingolipid Metabolism | Leaf | trans-AB | 21 | 14 | 0 | 0.514 | 1.000 |
| Starch and sucrose metabolism | Leaf | trans-AB | 98 | 62 | 3 | 2.275 | 0.399 |
| Steroid Biosynthesis | Leaf | trans-AB | 25 | 15 | 1 | 0.550 | 0.429 |
| Synthesis/Degradation of Ketone Bodies | Leaf | trans-AB | 8 | 8 | 0 | 0.294 | 1.000 |
| ALL | Leaf | trans-AB | 353 | 236 | 7 | 8.659 | 0.768 |
| Alpha-linoleic Acid Metabolism | Leaf | trans-ABC | 26 | 13 | 3 | 1.257 | 0.124 |
| Arachidonic Acid Metabolism | Leaf | trans-ABC | 10 | 7 | 1 | 0.677 | 0.509 |
| Biosynthesis of Unsaturated Fatty Acids | Leaf | trans-ABC | 33 | 19 | 1 | 1.837 | 0.855 |
| Cutin, Suberine, and Wax Biosynthesis | Leaf | trans-ABC | 10 | 6 | 1 | 0.580 | 0.457 |
| Ether Lipid Metabolism | Leaf | trans-ABC | 11 | 7 | 1 | 0.677 | 0.509 |
| Fatty Acid Biosynthesis | Leaf | trans-ABC | 32 | 19 | 2 | 1.837 | 0.561 |
| Fatty Acid Degradation | Leaf | trans-ABC | 34 | 30 | 3 | 2.901 | 0.565 |
| Fatty Acid Elongation | Leaf | trans-ABC | 16 | 9 | 0 | 0.870 | 1.000 |
| Glycerolipid Metabolism | Leaf | trans-ABC | 46 | 34 | 4 | 3.288 | 0.420 |
| Glycerophospholipid Metabolism | Leaf | trans-ABC | 64 | 47 | 3 | 4.545 | 0.846 |
| Linoleic Acid Metabolism | Leaf | trans-ABC | 12 | 5 | 2 | 0.484 | 0.077 |
| Sphingolipid Metabolism | Leaf | trans-ABC | 21 | 14 | 1 | 1.354 | 0.759 |
| Starch and sucrose metabolism | Leaf | trans-ABC | 98 | 62 | 3 | 5.996 | 0.947 |
| Steroid Biosynthesis | Leaf | trans-ABC | 25 | 15 | 3 | 1.451 | 0.171 |
| Synthesis/Degradation of Ketone Bodies | Leaf | trans-ABC | 8 | 8 | 1 | 0.774 | 0.557 |
| ALL | Leaf | trans-ABC | 353 | 236 | 18 | 22.822 | 0.884 |
| Alpha-linoleic Acid Metabolism | Stem | CCT-A | 26 | 15 | 0 | 0.030 | 1.000 |
| Arachidonic Acid Metabolism | Stem | CCT-A | 10 | 7 | 0 | 0.014 | 1.000 |
| Biosynthesis of Unsaturated Fatty Acids | Stem | CCT-A | 33 | 17 | 0 | 0.034 | 1.000 |
| Cutin, Suberine, and Wax Biosynthesis | Stem | CCT-A | 10 | 6 | 0 | 0.012 | 1.000 |
| Ether Lipid Metabolism | Stem | CCT-A | 11 | 7 | 0 | 0.014 | 1.000 |
| Fatty Acid Biosynthesis | Stem | CCT-A | 32 | 19 | 0 | 0.039 | 1.000 |
| Fatty Acid Degradation | Stem | CCT-A | 34 | 30 | 0 | 0.061 | 1.000 |
| Fatty Acid Elongation | Stem | CCT-A | 16 | 8 | 0 | 0.016 | 1.000 |
| Glycerolipid Metabolism | Stem | CCT-A | 46 | 32 | 0 | 0.065 | 1.000 |
| Glycerophospholipid Metabolism | Stem | CCT-A | 64 | 47 | 0 | 0.095 | 1.000 |
| Linoleic Acid Metabolism | Stem | CCT-A | 12 | 6 | 0 | 0.012 | 1.000 |
| Sphingolipid Metabolism | Stem | CCT-A | 21 | 14 | 0 | 0.028 | 1.000 |
| Starch and sucrose metabolism | Stem | CCT-A | 98 | 61 | 1 | 0.124 | 0.117 |
| Steroid Biosynthesis | Stem | CCT-A | 25 | 16 | 0 | 0.032 | 1.000 |
| Synthesis/Degradation of Ketone Bodies | Stem | CCT-A | 8 | 8 | 0 | 0.016 | 1.000 |
| ALL | Stem | CCT-A | 353 | 235 | 1 | 0.477 | 0.382 |
| Alpha-linoleic Acid Metabolism | Stem | CCT-AB | 26 | 15 | 1 | 0.486 | 0.390 |
| Arachidonic Acid Metabolism | Stem | CCT-AB | 10 | 7 | 0 | 0.227 | 1.000 |
| Biosynthesis of Unsaturated Fatty Acids | Stem | CCT-AB | 33 | 17 | 1 | 0.551 | 0.429 |
| Cutin, Suberine, and Wax Biosynthesis | Stem | CCT-AB | 10 | 6 | 0 | 0.194 | 1.000 |
| Ether Lipid Metabolism | Stem | CCT-AB | 11 | 7 | 1 | 0.227 | 0.206 |
| Fatty Acid Biosynthesis | Stem | CCT-AB | 32 | 19 | 1 | 0.615 | 0.465 |
| Fatty Acid Degradation | Stem | CCT-AB | 34 | 30 | 1 | 0.972 | 0.628 |
| Fatty Acid Elongation | Stem | CCT-AB | 16 | 8 | 0 | 0.259 | 1.000 |
| Glycerolipid Metabolism | Stem | CCT-AB | 46 | 32 | 0 | 1.037 | 1.000 |
| Glycerophospholipid Metabolism | Stem | CCT-AB | 64 | 47 | 2 | 1.523 | 0.453 |
| Linoleic Acid Metabolism | Stem | CCT-AB | 12 | 6 | 0 | 0.194 | 1.000 |
| Sphingolipid Metabolism | Stem | CCT-AB | 21 | 14 | 0 | 0.454 | 1.000 |
| Starch and sucrose metabolism | Stem | CCT-AB | 98 | 61 | 1 | 1.976 | 0.866 |
| Steroid Biosynthesis | Stem | CCT-AB | 25 | 16 | 1 | 0.518 | 0.410 |
| Synthesis/Degradation of Ketone Bodies | Stem | CCT-AB | 8 | 8 | 1 | 0.259 | 0.232 |
| ALL | Stem | CCT-AB | 353 | 235 | 8 | 7.613 | 0.494 |
| Alpha-linoleic Acid Metabolism | Stem | CCT-ABC | 26 | 15 | 3 | 1.546 | 0.196 |
| Arachidonic Acid Metabolism | Stem | CCT-ABC | 10 | 7 | 1 | 0.721 | 0.533 |
| Biosynthesis of Unsaturated Fatty Acids | Stem | CCT-ABC | 33 | 17 | 2 | 1.752 | 0.535 |
| Cutin, Suberine, and Wax Biosynthesis | Stem | CCT-ABC | 10 | 6 | 0 | 0.618 | 1.000 |
| Ether Lipid Metabolism | Stem | CCT-ABC | 11 | 7 | 2 | 0.721 | 0.157 |
| Fatty Acid Biosynthesis | Stem | CCT-ABC | 32 | 19 | 1 | 1.958 | 0.874 |
| Fatty Acid Degradation | Stem | CCT-ABC | 34 | 30 | 4 | 3.091 | 0.374 |
| Fatty Acid Elongation | Stem | CCT-ABC | 16 | 8 | 1 | 0.824 | 0.581 |
| Glycerolipid Metabolism | Stem | CCT-ABC | 46 | 32 | 1 | 3.297 | 0.969 |
| Glycerophospholipid Metabolism | Stem | CCT-ABC | 64 | 47 | 9 | 4.843 | 0.048 |
| Linoleic Acid Metabolism | Stem | CCT-ABC | 12 | 6 | 0 | 0.618 | 1.000 |
| Sphingolipid Metabolism | Stem | CCT-ABC | 21 | 14 | 0 | 1.443 | 1.000 |
| Starch and sucrose metabolism | Stem | CCT-ABC | 98 | 61 | 9 | 6.286 | 0.172 |
| Steroid Biosynthesis | Stem | CCT-ABC | 25 | 16 | 1 | 1.649 | 0.825 |
| Synthesis/Degradation of Ketone Bodies | Stem | CCT-ABC | 8 | 8 | 1 | 0.824 | 0.581 |
| ALL | Stem | CCT-ABC | 353 | 235 | 29 | 24.215 | 0.176 |
| Alpha-linoleic Acid Metabolism | Stem | trans-A | 26 | 15 | 0 | 0.006 | 1.000 |
| Arachidonic Acid Metabolism | Stem | trans-A | 10 | 7 | 0 | 0.003 | 1.000 |
| Biosynthesis of Unsaturated Fatty Acids | Stem | trans-A | 33 | 17 | 0 | 0.006 | 1.000 |
| Cutin, Suberine, and Wax Biosynthesis | Stem | trans-A | 10 | 6 | 0 | 0.002 | 1.000 |
| Ether Lipid Metabolism | Stem | trans-A | 11 | 7 | 0 | 0.003 | 1.000 |
| Fatty Acid Biosynthesis | Stem | trans-A | 32 | 19 | 0 | 0.007 | 1.000 |
| Fatty Acid Degradation | Stem | trans-A | 34 | 30 | 0 | 0.011 | 1.000 |
| Fatty Acid Elongation | Stem | trans-A | 16 | 8 | 0 | 0.003 | 1.000 |
| Glycerolipid Metabolism | Stem | trans-A | 46 | 32 | 0 | 0.012 | 1.000 |
| Glycerophospholipid Metabolism | Stem | trans-A | 64 | 47 | 0 | 0.018 | 1.000 |
| Linoleic Acid Metabolism | Stem | trans-A | 12 | 6 | 0 | 0.002 | 1.000 |
| Sphingolipid Metabolism | Stem | trans-A | 21 | 14 | 0 | 0.005 | 1.000 |
| Starch and sucrose metabolism | Stem | trans-A | 98 | 61 | 0 | 0.023 | 1.000 |
| Steroid Biosynthesis | Stem | trans-A | 25 | 16 | 0 | 0.006 | 1.000 |
| Synthesis/Degradation of Ketone Bodies | Stem | trans-A | 8 | 8 | 0 | 0.003 | 1.000 |
| ALL | Stem | trans-A | 353 | 235 | 0 | 0.088 | 1.000 |
| Alpha-linoleic Acid Metabolism | Stem | trans-AB | 26 | 15 | 0 | 0.168 | 1.000 |
| Arachidonic Acid Metabolism | Stem | trans-AB | 10 | 7 | 0 | 0.078 | 1.000 |
| Biosynthesis of Unsaturated Fatty Acids | Stem | trans-AB | 33 | 17 | 0 | 0.190 | 1.000 |
| Cutin, Suberine, and Wax Biosynthesis | Stem | trans-AB | 10 | 6 | 0 | 0.067 | 1.000 |
| Ether Lipid Metabolism | Stem | trans-AB | 11 | 7 | 0 | 0.078 | 1.000 |
| Fatty Acid Biosynthesis | Stem | trans-AB | 32 | 19 | 0 | 0.213 | 1.000 |
| Fatty Acid Degradation | Stem | trans-AB | 34 | 30 | 0 | 0.336 | 1.000 |
| Fatty Acid Elongation | Stem | trans-AB | 16 | 8 | 1 | 0.090 | 0.086 |
| Glycerolipid Metabolism | Stem | trans-AB | 46 | 32 | 0 | 0.358 | 1.000 |
| Glycerophospholipid Metabolism | Stem | trans-AB | 64 | 47 | 0 | 0.526 | 1.000 |
| Linoleic Acid Metabolism | Stem | trans-AB | 12 | 6 | 0 | 0.067 | 1.000 |
| Sphingolipid Metabolism | Stem | trans-AB | 21 | 14 | 0 | 0.157 | 1.000 |
| Starch and sucrose metabolism | Stem | trans-AB | 98 | 61 | 1 | 0.683 | 0.498 |
| Steroid Biosynthesis | Stem | trans-AB | 25 | 16 | 0 | 0.179 | 1.000 |
| Synthesis/Degradation of Ketone Bodies | Stem | trans-AB | 8 | 8 | 0 | 0.090 | 1.000 |
| ALL | Stem | trans-AB | 353 | 235 | 2 | 2.632 | 0.743 |
| Alpha-linoleic Acid Metabolism | Stem | trans-ABC | 26 | 15 | 0 | 0.602 | 1.000 |
| Arachidonic Acid Metabolism | Stem | trans-ABC | 10 | 7 | 0 | 0.281 | 1.000 |
| Biosynthesis of Unsaturated Fatty Acids | Stem | trans-ABC | 33 | 17 | 0 | 0.682 | 1.000 |
| Cutin, Suberine, and Wax Biosynthesis | Stem | trans-ABC | 10 | 6 | 0 | 0.241 | 1.000 |
| Ether Lipid Metabolism | Stem | trans-ABC | 11 | 7 | 0 | 0.281 | 1.000 |
| Fatty Acid Biosynthesis | Stem | trans-ABC | 32 | 19 | 0 | 0.763 | 1.000 |
| Fatty Acid Degradation | Stem | trans-ABC | 34 | 30 | 1 | 1.204 | 0.708 |
| Fatty Acid Elongation | Stem | trans-ABC | 16 | 8 | 1 | 0.321 | 0.279 |
| Glycerolipid Metabolism | Stem | trans-ABC | 46 | 32 | 1 | 1.284 | 0.731 |
| Glycerophospholipid Metabolism | Stem | trans-ABC | 64 | 47 | 1 | 1.886 | 0.855 |
| Linoleic Acid Metabolism | Stem | trans-ABC | 12 | 6 | 0 | 0.241 | 1.000 |
| Sphingolipid Metabolism | Stem | trans-ABC | 21 | 14 | 0 | 0.562 | 1.000 |
| Starch and sucrose metabolism | Stem | trans-ABC | 98 | 61 | 4 | 2.448 | 0.229 |
| Steroid Biosynthesis | Stem | trans-ABC | 25 | 16 | 1 | 0.642 | 0.481 |
| Synthesis/Degradation of Ketone Bodies | Stem | trans-ABC | 8 | 8 | 1 | 0.321 | 0.279 |
| ALL | Stem | trans-ABC | 353 | 235 | 8 | 9.432 | 0.732 |
