# Supplementary material for: The Role of cis Regulatory Evolution in Maize Domestication
Source: PLoS Genet. 2014 Nov 6;10(11):e1004745. doi: 10.1371/journal.pgen.1004745 (PMC4222645; doi:10.1371/journal.pgen.1004745)
Supplement: Table S14 — Significantly enriched and depleted GO terms from CCT and trans only gene lists including tissue, group, accession, description, counts, rate of occurrence, and FDR corrected p-values. (DOCX) [file pgen.1004745.s020.docx]

Table S14: Significantly enriched and depleted GO terms from CCT and *trans* only gene lists including tissue, group, accession, description, counts, rate of occurrence, and FDR corrected p-values.

| **Tissue** | **Group^1^** | **GO Accession** | **GO Description** | **Number Candidate Genes in Accession** | **Number genes in accession** | **Proportion in CCT genes** | **Proportion of assayed genes** | **FDR** |
| --- | --- | --- | --- | --- | --- | --- | --- | --- |
| Leaf | CCT-ABC | GO:0003677 | DNA binding ^2^ | 45 | 774 | 0.058 | 0.059 | 0.008 |
| Ear | *trans*-A | GO:0015995 | chlorophyll biosynthetic process | 3 | 13 | 0.231 | 0.001 | 0.045 |
| Ear | *trans*-AB | GO:0001071 | nucleic acid binding transcription factor activity | 26 | 228 | 0.114 | 0.017 | 0.0005 |
| Ear | *trans*-AB | GO:0003700 | sequence-specific DNA binding transcription factor activity | 26 | 228 | 0.114 | 0.017 | 0.0005 |
| Ear | *trans*-AB | GO:0006355 | regulation of transcription, DNA-dependent | 40 | 474 | 0.084 | 0.036 | 0.001 |
| Ear | *trans*-AB | GO:0043565 | sequence-specific DNA binding | 20 | 160 | 0.125 | 0.012 | 0.001 |
| Ear | *trans*-AB | GO:0015995 | chlorophyll biosynthetic process | 6 | 13 | 0.462 | 0.001 | 0.001 |
| Ear | *trans*-ABC | GO:0001071 | nucleic acid binding transcription factor activity | 45 | 228 | 0.197 | 0.017 | 0.0003 |
| Ear | *trans*-ABC | GO:0003700 | sequence-specific DNA binding transcription factor activity | 45 | 228 | 0.197 | 0.017 | 0.0003 |
| Ear | *trans*-ABC | GO:0006355 | regulation of transcription, DNA-dependent | 69 | 474 | 0.146 | 0.036 | 0.016 |
| Ear | *trans*-ABC | GO:0015995 | chlorophyll biosynthetic process | 7 | 13 | 0.538 | 0.001 | 0.018 |
| Leaf | *trans*-AB | GO:0005840 | ribosome | 30 | 295 | 0.107 | 0.022 | 0.002 |
| Leaf | *trans*-AB | GO:0019843 | rRNA binding | 6 | 19 | 0.316 | 0.001 | 0.036 |
| Leaf | *trans*-AB | GO:0051301 | cell division | 11 | 78 | 0.141 | 0.006 | 0.049 |
| Leaf | *trans*-ABC | GO:0051301 | cell division | 23 | 78 | 0.295 | 0.006 | 0.0005 |
| Leaf | *trans*-ABC | GO:0000166 | nucleotide binding | 170 | 1329 | 0.128 | 0.101 | 0.034 |
| Leaf | *trans*-ABC | GO:0007049 | cell cycle | 19 | 77 | 0.247 | 0.006 | 0.035 |

^1^ Group indicating the candidate list (CCT or consistent *trans* only) and level of list (A, AB, or ABC list).

^2^ Under-represented GO term.
