# Supplementary material for: The Role of cis Regulatory Evolution in Maize Domestication
Source: PLoS Genet. 2014 Nov 6;10(11):e1004745. doi: 10.1371/journal.pgen.1004745 (PMC4222645; doi:10.1371/journal.pgen.1004745)
Supplement: Table S15 — Numbers of biological replicates of F1 hybrid and parent inbred lines for RNAseq expression study with hybrid replicates internal and parent around the perimeter (shaded gray). (DOCX) [file pgen.1004745.s021.docx]

Table S15: Numbers of biological replicates of F_1_ hybrid and parent inbred lines for RNAseq expression study with hybrid replicates internal and parent around the perimeter (shaded gray).

|  | Inbred | B73 | CML103 | Ki3 | Mo17 | Oh43 | W22 |
| --- | --- | --- | --- | --- | --- | --- | --- |
| Inbred |  | 2/2/2 | 2/2/2 | 2/2/2 | 2/2/2 | 2/2/2 | 2/2/2 |
| TIL01 | 2/2/2 | 2/2/2 |  |  | 0/2/2 | 2/2/2 | 2/2/2 |
| TIL03 | 2/1/1 | 2/1/1 | 2/2/2 | 1/2/2 |  | 1/2/2 | 2/2/2 |
| TIL05 | 2/2/2 | 2/2/2 |  |  |  |  |  |
| TIL09 | 2/2/2 | 2/2/1 |  | 2/2/2 | 3/2/2 | 2/2/2 |  |
| TIL10 | 2/2/2 |  |  |  |  | 1/2/2 |  |
| TIL11 | 2/2/2 | 2/2/2 | 2/2/2 | 2/2/2 |  | 2/2/2 | 2/2/2 |
| TIL14 | 2/2/2 | 4/2/2 | 2/2/2 | 2/1/2 | 2/2/2 |  | 1/2/2 |
| TIL15 | 2/2/2 |  |  |  |  | 2/2/2 |  |
| TIL25 | 2/2/2 | 4/3/2 |  |  |  | 3/2/2 | 2/2/2 |

The first, second and third number in each cell are for ear/leaf/stem.
