# Supplementary material for: The Role of cis Regulatory Evolution in Maize Domestication
Source: PLoS Genet. 2014 Nov 6;10(11):e1004745. doi: 10.1371/journal.pgen.1004745 (PMC4222645; doi:10.1371/journal.pgen.1004745)
Supplement: Table S16 — Adapter name, barcode sequence, and barcode length for Illumina adapters used in RNAseq libraries. (DOCX) [file pgen.1004745.s022.docx]

Table S16: Adapter name, barcode sequence, and barcode length for Illumina adapters used in RNAseq libraries.

| **Adapter #** | **Adapter Name** | **Barcode Sequence** | **Barcode Length** |
| --- | --- | --- | --- |
| **1** | PE YC3 | GCATGT | 5 nt |
| **2** | PE YC4 | TGTGCT | 5 nt |
| **3** | PE YC5 | AGTCAT | 5 nt |
| **4** | PE YC6 | GTAAGT | 5 nt |
| **5** | PE YC7 | TCCTCT | 5 nt |
| **6** | PE YC8 | CAGGTT | 5 nt |
| **7** | PE JM 1 | TCCAT | 4 nt |
| **8** | PE JM 2 | TAGCT | 4 nt |
| **9** | PE JM 3 | GTTCT | 4 nt |
| **10** | PE JM 4 | CGATT | 4 nt |
| **11** | PE TB 1 | ATCGT | 4 nt |
| **12** | PE TB 2 | GCTAT | 4 nt |
| **13** | PE TB 3 | TGGAT | 4 nt |
| **14** | PE TB 4 | ATGCT | 4 nt |
| **15** | PE ZL1 | CACTAT | 5 nt |
