# Supplementary material for: The Role of cis Regulatory Evolution in Maize Domestication
Source: PLoS Genet. 2014 Nov 6;10(11):e1004745. doi: 10.1371/journal.pgen.1004745 (PMC4222645; doi:10.1371/journal.pgen.1004745)
Supplement: Table S17 — Number of genomic paired end reads and coverage obtained for constructing pseudo-transcriptomes. (DOCX) [file pgen.1004745.s023.docx]

Table S17: Number of genomic paired-end reads and coverage obtained for constructing pseudo-transcriptomes.

| **Inbred Line** | **# Reads** | **genome coverage** |
| --- | --- | --- |
| CML103 | 4.46E+08 | 21.24 |
| Ki3 | 4.38E+08 | 19.85 |
| Mo17 | 2.57E+08 | 11.37 |
| Oh43 | 5.59E+08 | 20.56 |
| TI01 | 3.44E+08 | 14.5 |
| TI03 | 3.16E+08 | 13.15 |
| TI05 | 4.76E+08 | 17.8 |
| TI09 | 3.42E+08 | 15.21 |
| TI10 | 5.29E+08 | 24.29 |
| TI11 | 3.41E+08 | 15.97 |
| TI14 | 3.22E+08 | 13.82 |
| TI15 | 5.39E+08 | 24.22 |
| TI25 | 4.27E+08 | 19.93 |
| W22 | 3.07E+08 | 13.19 |
| Average | 4.03E+08 | 17.50714 |
