# Supplementary material for: The Role of cis Regulatory Evolution in Maize Domestication
Source: PLoS Genet. 2014 Nov 6;10(11):e1004745. doi: 10.1371/journal.pgen.1004745 (PMC4222645; doi:10.1371/journal.pgen.1004745)
Supplement: Table S18 — F1 and parent maize∶teosinte read depth ratios for the 29 maize-teosinte comparisons. (DOCX) [file pgen.1004745.s024.docx]

Table S18: F_1_ and parent maize:teosinte read depth ratios for the 29 maize-teosinte comparisons.

| **Maize Inbred Line** | **Teo Inbred Line** | **Tissue** | **F_1_ Maize** | **F_1_ Teo** | **F_1_ Maize: Teo** | **Parent Maize** | **Parent Teo** | **Parent Maize: Teo** |
| --- | --- | --- | --- | --- | --- | --- | --- | --- |
| B73 | TI01 | Ear | 548658 | 535692 | 1.024 | 1339974 | 1363641 | 0.983 |
| B73 | TI03 | Ear | 1332962 | 1308315 | 1.019 | 1340408 | 1525404 | 0.879 |
| B73 | TI05 | Ear | 1392308 | 1222311 | 1.139 | 1469974 | 1302726 | 1.128 |
| B73 | TI09 | Ear | 1274064 | 1239145 | 1.028 | 1547180 | 1588708 | 0.974 |
| B73 | TI11 | Ear | 1256619 | 1228425 | 1.023 | 1445006 | 1562947 | 0.925 |
| B73 | TI14 | Ear | 2074683 | 1995865 | 1.039 | 1236656 | 1416900 | 0.873 |
| B73 | TI25 | Ear | 3456903 | 3368114 | 1.026 | 1994057 | 2139121 | 0.932 |
| CML103 | TI03 | Ear | 1164973 | 1187798 | 0.981 | 1557274 | 1444854 | 1.078 |
| CML103 | TI11 | Ear | 1269732 | 1326158 | 0.957 | 1642249 | 1464618 | 1.121 |
| CML103 | TI14 | Ear | 1013806 | 1041822 | 0.973 | 1424549 | 1340820 | 1.062 |
| Ki3 | TI03 | Ear | 617137 | 606828 | 1.017 | 1726466 | 1427226 | 1.210 |
| Ki3 | TI09 | Ear | 1162241 | 1132419 | 1.026 | 1943166 | 1474948 | 1.317 |
| Ki3 | TI11 | Ear | 1448147 | 1398338 | 1.036 | 1863953 | 1476410 | 1.262 |
| Ki3 | TI14 | Ear | 993927 | 976965 | 1.017 | 1581755 | 1328591 | 1.191 |
| Mo17 | TI09 | Ear | 1958806 | 1880818 | 1.041 | 1658029 | 1483218 | 1.118 |
| Mo17 | TI14 | Ear | 1246819 | 1220213 | 1.022 | 1310484 | 1336602 | 0.980 |
| Oh43 | TI01 | Ear | 682952 | 616802 | 1.107 | 1416268 | 942657 | 1.502 |
| Oh43 | TI03 | Ear | 686516 | 677380 | 1.013 | 1415084 | 1415957 | 0.999 |
| Oh43 | TI09 | Ear | 1226005 | 1183559 | 1.036 | 1596935 | 1418369 | 1.126 |
| Oh43 | TI10 | Ear | 1506506 | 1441053 | 1.045 | 1938758 | 1926464 | 1.006 |
| Oh43 | TI11 | Ear | 1682156 | 1645563 | 1.022 | 1510584 | 1418991 | 1.065 |
| Oh43 | TI15 | Ear | 1724507 | 1650954 | 1.045 | 2148502 | 2115180 | 1.016 |
| Oh43 | TI25 | Ear | 2527003 | 2462697 | 1.026 | 1991136 | 1870861 | 1.064 |
| W22 | TI01 | Ear | 1000021 | 1004214 | 0.996 | 1133016 | 981002 | 1.155 |
| W22 | TI03 | Ear | 1008985 | 1022282 | 0.987 | 1132417 | 1485944 | 0.762 |
| W22 | TI11 | Ear | 1225371 | 1277373 | 0.959 | 1205877 | 1500105 | 0.804 |
| W22 | TI14 | Ear | 581738 | 596940 | 0.975 | 1037965 | 1368023 | 0.759 |
| W22 | TI25 | Ear | 1610782 | 1634502 | 0.985 | 1604672 | 1964191 | 0.817 |
| B73 | TI01 | Leaf | 614147 | 593725 | 1.034 | 1304947 | 843494 | 1.547 |
| B73 | TI03 | Leaf | 722405 | 706888 | 1.022 | 1317628 | 740227 | 1.780 |
| B73 | TI05 | Leaf | 1519969 | 1339917 | 1.134 | 1484715 | 1376235 | 1.079 |
| B73 | TI09 | Leaf | 1180324 | 1118945 | 1.055 | 1544089 | 1628858 | 0.948 |
| B73 | TI11 | Leaf | 1796345 | 1768058 | 1.016 | 1402705 | 1590793 | 0.882 |
| B73 | TI14 | Leaf | 771348 | 760024 | 1.015 | 1215483 | 1412315 | 0.861 |
| B73 | TI25 | Leaf | 3187576 | 3123353 | 1.021 | 1941537 | 2124205 | 0.914 |
| CML103 | TI03 | Leaf | 963230 | 993682 | 0.969 | 1081099 | 716207 | 1.509 |
| CML103 | TI11 | Leaf | 1228084 | 1286509 | 0.955 | 1096632 | 1476668 | 0.743 |
| CML103 | TI14 | Leaf | 1280894 | 1300407 | 0.985 | 961537 | 1328958 | 0.724 |
| Ki3 | TI03 | Leaf | 971472 | 943044 | 1.030 | 1487526 | 695060 | 2.140 |
| Ki3 | TI09 | Leaf | 1025711 | 979150 | 1.048 | 1689534 | 1487056 | 1.136 |
| Ki3 | TI11 | Leaf | 1549114 | 1519538 | 1.019 | 1576152 | 1499228 | 1.051 |
| Ki3 | TI14 | Leaf | 620782 | 599048 | 1.036 | 1315165 | 1304559 | 1.008 |
| Mo17 | TI01 | Leaf | 1138774 | 1093467 | 1.041 | 1301346 | 788342 | 1.651 |
| Mo17 | TI09 | Leaf | 1613073 | 1543590 | 1.045 | 1526586 | 1523472 | 1.002 |
| Mo17 | TI14 | Leaf | 1275297 | 1247578 | 1.022 | 1200954 | 1334127 | 0.900 |
| Oh43 | TI01 | Leaf | 934191 | 889704 | 1.050 | 1317475 | 763348 | 1.726 |
| Oh43 | TI03 | Leaf | 900744 | 880510 | 1.023 | 1340806 | 675853 | 1.984 |
| Oh43 | TI09 | Leaf | 1364811 | 1230586 | 1.109 | 1498127 | 1430481 | 1.047 |
| Oh43 | TI10 | Leaf | 1330090 | 1271902 | 1.046 | 1890127 | 2016184 | 0.937 |
| Oh43 | TI11 | Leaf | 1536284 | 1486283 | 1.034 | 1386565 | 1424464 | 0.973 |
| Oh43 | TI15 | Leaf | 1409167 | 1332572 | 1.057 | 2079685 | 2300457 | 0.904 |
| Oh43 | TI25 | Leaf | 1998547 | 1994485 | 1.002 | 1849022 | 1832188 | 1.009 |
| W22 | TI01 | Leaf | 949562 | 948154 | 1.001 | 997523 | 807510 | 1.235 |
| W22 | TI03 | Leaf | 1089639 | 1094997 | 0.995 | 1022238 | 726916 | 1.406 |
| W22 | TI11 | Leaf | 1222795 | 1271521 | 0.962 | 1047670 | 1510414 | 0.694 |
| W22 | TI14 | Leaf | 959118 | 1011552 | 0.948 | 918896 | 1359265 | 0.676 |
| W22 | TI25 | Leaf | 1817700 | 1794040 | 1.013 | 1426677 | 1939475 | 0.736 |
| B73 | TI01 | Stem | 814435 | 794588 | 1.025 | 1479151 | 1016998 | 1.454 |
| B73 | TI03 | Stem | 654052 | 638347 | 1.025 | 1470660 | 697646 | 2.108 |
| B73 | TI05 | Stem | 1390695 | 1232787 | 1.128 | 1632831 | 1458056 | 1.120 |
| B73 | TI09 | Stem | 1063362 | 1030058 | 1.032 | 1725026 | 1582994 | 1.090 |
| B73 | TI11 | Stem | 1709106 | 1676796 | 1.019 | 1581561 | 1611862 | 0.981 |
| B73 | TI14 | Stem | 1503683 | 1473554 | 1.020 | 1358245 | 1152415 | 1.179 |
| B73 | TI25 | Stem | 2559312 | 2509334 | 1.020 | 2204511 | 1936663 | 1.138 |
| CML103 | TI03 | Stem | 1630067 | 1654612 | 0.985 | 1046551 | 673020 | 1.555 |
| CML103 | TI11 | Stem | 1624132 | 1735240 | 0.936 | 1070394 | 1497879 | 0.715 |
| CML103 | TI14 | Stem | 1406737 | 1442810 | 0.975 | 921490 | 1064439 | 0.866 |
| Ki3 | TI03 | Stem | 1564709 | 1524616 | 1.026 | 1383812 | 663211 | 2.087 |
| Ki3 | TI09 | Stem | 1360852 | 1277590 | 1.065 | 1570617 | 1463943 | 1.073 |
| Ki3 | TI11 | Stem | 1506271 | 1471533 | 1.024 | 1466064 | 1533337 | 0.956 |
| Ki3 | TI14 | Stem | 1433974 | 1397364 | 1.026 | 1210270 | 1065864 | 1.135 |
| Mo17 | TI01 | Stem | 1445541 | 1383394 | 1.045 | 1339462 | 953847 | 1.404 |
| Mo17 | TI09 | Stem | 1755099 | 1664349 | 1.055 | 1568777 | 1493021 | 1.051 |
| Mo17 | TI14 | Stem | 1509504 | 1465292 | 1.030 | 1227890 | 1085299 | 1.131 |
| Oh43 | TI01 | Stem | 2228997 | 2122547 | 1.050 | 1296435 | 917137 | 1.414 |
| Oh43 | TI03 | Stem | 1514809 | 1476199 | 1.026 | 1299271 | 641848 | 2.024 |
| Oh43 | TI09 | Stem | 1681694 | 1534144 | 1.096 | 1478440 | 1407299 | 1.051 |
| Oh43 | TI10 | Stem | 2202089 | 2114800 | 1.041 | 1817984 | 1931009 | 0.941 |
| Oh43 | TI11 | Stem | 842869 | 832338 | 1.013 | 1376958 | 1459489 | 0.943 |
| Oh43 | TI15 | Stem | 2232337 | 2125974 | 1.050 | 2017899 | 1879172 | 1.074 |
| Oh43 | TI25 | Stem | 2015348 | 1970527 | 1.023 | 1851354 | 1696149 | 1.092 |
| W22 | TI01 | Stem | 1545802 | 1552932 | 0.995 | 1144455 | 968242 | 1.182 |
| W22 | TI03 | Stem | 1674588 | 1710410 | 0.979 | 1164236 | 692688 | 1.681 |
| W22 | TI11 | Stem | 1693814 | 1720733 | 0.984 | 1224561 | 1545934 | 0.792 |
| W22 | TI14 | Stem | 1230948 | 1274305 | 0.966 | 1047982 | 1099034 | 0.954 |
| W22 | TI25 | Stem | 2347061 | 2406159 | 0.975 | 1638250 | 1779791 | 0.920 |
