# Supplementary material for: The Role of cis Regulatory Evolution in Maize Domestication
Source: PLoS Genet. 2014 Nov 6;10(11):e1004745. doi: 10.1371/journal.pgen.1004745 (PMC4222645; doi:10.1371/journal.pgen.1004745)
Supplement: Table S19 — Spearman correlation between all biological replicate pairs of the same genotype. (DOCX) [file pgen.1004745.s025.docx]

Table S19: Spearman correlation between all biological replicate pairs of the same genotype.

| Genotype | Tissue | BioRep1 | BioRep2 | Correlation |
| --- | --- | --- | --- | --- |
| B73_TI01 | Ear | B73_TI01_2_PL0006 | B73_TI01_3_PL0004 | 0.953 |
| B73_TI03 | Ear | B73_TI03_1_PL0004 | B73_TI03_4_PL0006 | 0.974 |
| B73_TI05 | Ear | B73_TI05_12_PL0003 | B73_TI05_5_PL0006 | 0.966 |
| B73_TI09 | Ear | B73_TI09_1_PL0001 | B73_TI09_8_PL0003 | 0.976 |
| B73_TI11 | Ear | B73_TI11_4_PL0004 | B73_TI11_6_PL0003 | 0.973 |
| B73_TI14 | Ear | B73_TI14_11_PL0001 | B73_TI14_3_PL0001 | 0.970 |
| B73_TI14 | Ear | B73_TI14_11_PL0001 | B73_TI14_7_PL0001 | 0.968 |
| B73_TI14 | Ear | B73_TI14_11_PL0001 | B73_TI14_9_PL0001 | 0.973 |
| B73_TI14 | Ear | B73_TI14_3_PL0001 | B73_TI14_7_PL0001 | 0.974 |
| B73_TI14 | Ear | B73_TI14_3_PL0001 | B73_TI14_9_PL0001 | 0.965 |
| B73_TI14 | Ear | B73_TI14_7_PL0001 | B73_TI14_9_PL0001 | 0.962 |
| B73_TI25 | Ear | B73_TI25_10_PL0004 | B73_TI25_11_PL0002 | 0.979 |
| B73_TI25 | Ear | B73_TI25_10_PL0004 | B73_TI25_14_PL0001 | 0.975 |
| B73_TI25 | Ear | B73_TI25_10_PL0004 | B73_TI25_4_PL0001 | 0.977 |
| B73_TI25 | Ear | B73_TI25_11_PL0002 | B73_TI25_14_PL0001 | 0.978 |
| B73_TI25 | Ear | B73_TI25_11_PL0002 | B73_TI25_4_PL0001 | 0.978 |
| B73_TI25 | Ear | B73_TI25_14_PL0001 | B73_TI25_4_PL0001 | 0.979 |
| CML103_TI03 | Ear | CML103_TI03_2_PL0001 | CML103_TI03_9_PL0004 | 0.957 |
| CML103_TI11 | Ear | CML103_TI11_11_PL0004 | CML103_TI11_13_PL0003 | 0.977 |
| CML103_TI14 | Ear | CML103_TI14_12_PL0004 | CML103_TI14_6_PL0001 | 0.970 |
| CML103_TI25 | Ear | CML103_TI25_1_PL0002 | CML103_TI25_12_PL0002 | 0.975 |
| Ki3_TI03 | Ear | Ki3_TI03_10_PL0002 | NA | ^a^ |
| Ki3_TI09 | Ear | Ki3_TI09_14_PL0002 | Ki3_TI09_6_PL0004 | 0.967 |
| Ki3_TI11 | Ear | Ki3_TI11_7_PL0003 | Ki3_TI11_9_PL0002 | 0.981 |
| Ki3_TI14 | Ear | Ki3_TI14_11_PL0003 | Ki3_TI14_3_PL0002 | 0.975 |
| Mo17_TI09 | Ear | Mo17_TI09_13_PL0001 | Mo17_TI09_13_PL0004 | 0.974 |
| Mo17_TI09 | Ear | Mo17_TI09_13_PL0001 | Mo17_TI09_5_PL0001 | 0.975 |
| Mo17_TI09 | Ear | Mo17_TI09_13_PL0004 | Mo17_TI09_5_PL0001 | 0.971 |
| Mo17_TI14 | Ear | Mo17_TI14_7_PL0002 | Mo17_TI14_7_PL0004 | 0.975 |
| Oh43_TI01 | Ear | Oh43_TI01_9_PL0003 | NA | ^a^ |
| Oh43_TI03 | Ear | Oh43_TI03_5_PL0004 | NA | ^a^ |
| Oh43_TI09 | Ear | Oh43_TI09_14_PL0003 | Oh43_TI09_8_PL0004 | 0.974 |
| Oh43_TI10 | Ear | Oh43_TI10_1_PL0003 | Oh43_TI10_1_PL0006 | 0.982 |
| Oh43_TI11 | Ear | Oh43_TI11_10_PL0001 | Oh43_TI11_13_PL0006 | 0.847 |
| Oh43_TI11 | Ear | Oh43_TI11_10_PL0001 | Oh43_TI11_8_PL0001 | 0.975 |
| Oh43_TI11 | Ear | Oh43_TI11_13_PL0006 | Oh43_TI11_8_PL0001 | 0.850 |
| Oh43_TI15 | Ear | Oh43_TI15_12_PL0001 | Oh43_TI15_5_PL0003 | 0.974 |
| Oh43_TI25 | Ear | Oh43_TI25_13_PL0002 | Oh43_TI25_2_PL0002 | 0.976 |
| Oh43_TI25 | Ear | Oh43_TI25_13_PL0002 | Oh43_TI25_8_PL0002 | 0.983 |
| Oh43_TI25 | Ear | Oh43_TI25_2_PL0002 | Oh43_TI25_8_PL0002 | 0.981 |
| W22_TI01 | Ear | W22_TI01_2_PL0003 | W22_TI01_4_PL0002 | 0.970 |
| W22_TI03 | Ear | W22_TI03_3_PL0003 | W22_TI03_6_PL0002 | 0.974 |
| W22_TI11 | Ear | W22_TI11_2_PL0004 | W22_TI11_4_PL0003 | 0.974 |
| W22_TI14 | Ear | W22_TI14_10_PL0003 | NA | ^a^ |
| W22_TI25 | Ear | W22_TI25_14_PL0004 | W22_TI25_5_PL0002 | 0.972 |
| B73_TI01 | Leaf | B73_TI01_1_PL0009 | B73_TI01_13_PL0005 | 0.930 |
| B73_TI03 | Leaf | B73_TI03_10_PL0005 | NA | ^a^ |
| B73_TI05 | Leaf | B73_TI05_6_PL0006 | B73_TI05_9_PL0009 | 0.964 |
| B73_TI09 | Leaf | B73_TI09_8_PL0007 | B73_TI09_9_PL0005 | 0.908 |
| B73_TI11 | Leaf | B73_TI11_4_PL0008 | B73_TI11_5_PL0009 | 0.935 |
| B73_TI14 | Leaf | B73_TI14_1_PL0007 | B73_TI14_2_PL0005 | 0.930 |
| B73_TI25 | Leaf | B73_TI25_10_PL0009 | B73_TI25_12_PL0005 | 0.940 |
| B73_TI25 | Leaf | B73_TI25_10_PL0009 | B73_TI25_7_PL0006 | 0.947 |
| B73_TI25 | Leaf | B73_TI25_12_PL0005 | B73_TI25_7_PL0006 | 0.879 |
| CML103_TI03 | Leaf | CML103_TI03_5_PL0007 | CML103_TI03_7_PL0005 | 0.959 |
| CML103_TI11 | Leaf | CML103_TI11_11_PL0009 | CML103_TI11_8_PL0006 | 0.956 |
| CML103_TI14 | Leaf | CML103_TI14_12_PL0009 | CML103_TI14_9_PL0006 | 0.964 |
| CML103_TI25 | Leaf | CML103_TI25_3_PL0009 | CML103_TI25_6_PL0008 | 0.930 |
| Ki3_TI03 | Leaf | Ki3_TI03_2_PL0007 | Ki3_TI03_3_PL0005 | 0.946 |
| Ki3_TI09 | Leaf | Ki3_TI09_12_PL0007 | Ki3_TI09_2_PL0008 | 0.933 |
| Ki3_TI11 | Leaf | Ki3_TI11_4_PL0009 | Ki3_TI11_7_PL0008 | 0.963 |
| Ki3_TI14 | Leaf | Ki3_TI14_8_PL0008 | NA | ^a^ |
| Mo17_TI01 | Leaf | Mo17_TI01_13_PL0007 | Mo17_TI01_3_PL0008 | 0.879 |
| Mo17_TI09 | Leaf | Mo17_TI09_11_PL0005 | Mo17_TI09_13_PL0009 | 0.909 |
| Mo17_TI14 | Leaf | Mo17_TI14_12_PL0008 | Mo17_TI14_6_PL0009 | 0.813 |
| Mo17_TI25 | Leaf | Mo17_TI25_13_PL0008 | Mo17_TI25_6_PL0005 | 0.944 |
| Oh43_TI01 | Leaf | Oh43_TI01_1_PL0008 | Oh43_TI01_6_PL0007 | 0.961 |
| Oh43_TI03 | Leaf | Oh43_TI03_3_PL0007 | Oh43_TI03_4_PL0005 | 0.954 |
| Oh43_TI09 | Leaf | Oh43_TI09_10_PL0006 | Oh43_TI09_14_PL0009 | 0.944 |
| Oh43_TI10 | Leaf | Oh43_TI10_1_PL0005 | Oh43_TI10_4_PL0007 | 0.811 |
| Oh43_TI11 | Leaf | Oh43_TI11_14_PL0008 | Oh43_TI11_8_PL0009 | 0.969 |
| Oh43_TI15 | Leaf | Oh43_TI15_14_PL0005 | Oh43_TI15_9_PL0007 | 0.875 |
| Oh43_TI25 | Leaf | Oh43_TI25_10_PL0008 | Oh43_TI25_7_PL0009 | 0.950 |
| W22_TI01 | Leaf | W22_TI01_14_PL0007 | W22_TI01_5_PL0008 | 0.961 |
| W22_TI03 | Leaf | W22_TI03_11_PL0007 | W22_TI03_9_PL0008 | 0.940 |
| W22_TI11 | Leaf | W22_TI11_5_PL0005 | W22_TI11_7_PL0007 | 0.880 |
| W22_TI14 | Leaf | W22_TI14_10_PL0007 | W22_TI14_2_PL0009 | 0.828 |
| W22_TI25 | Leaf | W22_TI25_11_PL0008 | W22_TI25_8_PL0005 | 0.972 |
| B73_TI01 | Stem | B73_TI01_4_PL0011 | B73_TI01_4_PL0013 | 0.949 |
| B73_TI03 | Stem | B73_TI03_2_PL0011 | NA | ^a^ |
| B73_TI05 | Stem | B73_TI05_14_PL0006 | B73_TI05_14_PL0012 | 0.961 |
| B73_TI09 | Stem | B73_TI09_11_PL0010 | NA | ^a^ |
| B73_TI11 | Stem | B73_TI11_12_PL0006 | B73_TI11_13_PL0011 | 0.961 |
| B73_TI14 | Stem | B73_TI14_1_PL0010 | B73_TI14_1_PL0011 | 0.954 |
| B73_TI25 | Stem | B73_TI25_10_PL0013 | B73_TI25_3_PL0012 | 0.941 |
| CML103_TI03 | Stem | CML103_TI03_7_PL0010 | CML103_TI03_9_PL0011 | 0.961 |
| CML103_TI11 | Stem | CML103_TI11_11_PL0013 | CML103_TI11_12_PL0010 | 0.975 |
| CML103_TI14 | Stem | CML103_TI14_12_PL0013 | CML103_TI14_13_PL0010 | 0.964 |
| CML103_TI25 | Stem | CML103_TI25_12_PL0011 | CML103_TI25_5_PL0012 | 0.964 |
| Ki3_TI03 | Stem | Ki3_TI03_1_PL0012 | Ki3_TI03_3_PL0010 | 0.941 |
| Ki3_TI09 | Stem | Ki3_TI09_11_PL0006 | Ki3_TI09_8_PL0011 | 0.947 |
| Ki3_TI11 | Stem | Ki3_TI11_14_PL0011 | Ki3_TI11_6_PL0012 | 0.965 |
| Ki3_TI14 | Stem | Ki3_TI14_7_PL0012 | Ki3_TI14_8_PL0013 | 0.961 |
| Mo17_TI01 | Stem | Mo17_TI01_11_PL0011 | Mo17_TI01_7_PL0013 | 0.955 |
| Mo17_TI09 | Stem | Mo17_TI09_13_PL0013 | Mo17_TI09_3_PL0011 | 0.951 |
| Mo17_TI14 | Stem | Mo17_TI14_11_PL0012 | Mo17_TI14_9_PL0013 | 0.961 |
| Mo17_TI25 | Stem | Mo17_TI25_12_PL0012 | Mo17_TI25_6_PL0010 | 0.966 |
| Oh43_TI01 | Stem | Oh43_TI01_3_PL0006 | Oh43_TI01_5_PL0013 | 0.937 |
| Oh43_TI01 | Stem | Oh43_TI01_3_PL0006 | Oh43_TI01_8_PL0010 | 0.944 |
| Oh43_TI01 | Stem | Oh43_TI01_5_PL0013 | Oh43_TI01_8_PL0010 | 0.965 |
| Oh43_TI03 | Stem | Oh43_TI03_2_PL0013 | Oh43_TI03_4_PL0010 | 0.967 |
| Oh43_TI09 | Stem | Oh43_TI09_14_PL0010 | Oh43_TI09_14_PL0013 | 0.972 |
| Oh43_TI10 | Stem | Oh43_TI10_3_PL0013 | Oh43_TI10_5_PL0010 | 0.976 |
| Oh43_TI11 | Stem | Oh43_TI11_13_PL0012 | NA | ^a^ |
| Oh43_TI15 | Stem | Oh43_TI15_2_PL0012 | Oh43_TI15_5_PL0011 | 0.976 |
| Oh43_TI25 | Stem | Oh43_TI25_2_PL0010 | Oh43_TI25_9_PL0012 | 0.953 |
| W22_TI01 | Stem | W22_TI01_10_PL0011 | W22_TI01_4_PL0012 | 0.968 |
| W22_TI03 | Stem | W22_TI03_7_PL0011 | W22_TI03_8_PL0012 | 0.957 |
| W22_TI11 | Stem | W22_TI11_1_PL0013 | W22_TI11_9_PL0010 | 0.967 |
| W22_TI14 | Stem | W22_TI14_6_PL0011 | W22_TI14_6_PL0013 | 0.970 |
| W22_TI25 | Stem | W22_TI25_10_PL0010 | W22_TI25_10_PL0012 | 0.947 |
| B73 | Ear | B73_1_PL0014 | B73_1_PL0015 | 0.982 |
| CML103 | Ear | CML103_2_PL0014 | CML103_2_PL0015 | 0.977 |
| Ki3 | Ear | Ki3_3_PL0014 | Ki3_3_PL0015 | 0.939 |
| Mo17 | Ear | Mo17_4_PL0014 | Mo17_4_PL0015 | 0.975 |
| Oh43 | Ear | Oh43_5_PL0014 | Oh43_5_PL0015 | 0.983 |
| TIL01 | Ear | TIL01_6_PL0014 | TIL01_6_PL0015 | 0.948 |
| TIL03 | Ear | TIL03_7_PL0014 | TIL03_7_PL0015 | 0.916 |
| TIL05 | Ear | TIL05_8_PL0014 | TIL05_8_PL0015 | 0.934 |
| TIL09 | Ear | TIL09_15_PL0014 | TIL09_9_PL0015 | 0.969 |
| TIL10 | Ear | TIL10_10_PL0015 | TIL10_9_PL0014 | 0.955 |
| TIL11 | Ear | TIL11_10_PL0014 | TIL11_11_PL0015 | 0.972 |
| TIL14 | Ear | TIL14_11_PL0014 | TIL14_12_PL0015 | 0.972 |
| TIL15 | Ear | TIL15_12_PL0014 | TIL15_13_PL0015 | 0.965 |
| TIL25 | Ear | TIL25_13_PL0014 | TIL25_14_PL0015 | 0.959 |
| W22 | Ear | W22_14_PL0014 | W22_15_PL0015 | 0.969 |
| B73 | Leaf | B73_1_PL0016 | B73_1_PL0017 | 0.952 |
| CML103 | Leaf | CML103_2_PL0016 | CML103_2_PL0017 | 0.971 |
| Ki3 | Leaf | Ki3_3_PL0016 | Ki3_3_PL0017 | 0.968 |
| Mo17 | Leaf | Mo17_4_PL0016 | Mo17_4_PL0017 | 0.883 |
| Oh43 | Leaf | Oh43_5_PL0016 | Oh43_5_PL0017 | 0.962 |
| TIL01 | Leaf | TIL01_6_PL0016 | TIL01_6_PL0017 | 0.949 |
| TIL03 | Leaf | TIL03_7_PL0016 | NA | ^a^ |
| TIL05 | Leaf | TIL05_7_PL0017 | TIL05_8_PL0016 | 0.925 |
| TIL09 | Leaf | TIL09_8_PL0017 | TIL09_9_PL0016 | 0.959 |
| TIL10 | Leaf | TIL10_10_PL0016 | TIL10_9_PL0017 | 0.964 |
| TIL11 | Leaf | TIL11_10_PL0017 | TIL11_11_PL0016 | 0.956 |
| TIL14 | Leaf | TIL14_11_PL0017 | TIL14_12_PL0016 | 0.964 |
| TIL15 | Leaf | TIL15_12_PL0017 | TIL15_13_PL0016 | 0.970 |
| TIL25 | Leaf | TIL25_13_PL0017 | TIL25_14_PL0016 | 0.968 |
| W22 | Leaf | W22_14_PL0017 | W22_15_PL0016 | 0.962 |
| B73 | Stem | B73_1_PL0018 | B73_1_PL0019 | 0.929 |
| CML103 | Stem | CML103_2_PL0018 | CML103_2_PL0019 | 0.960 |
| Ki3 | Stem | Ki3_3_PL0018 | Ki3_3_PL0019 | 0.963 |
| Mo17 | Stem | Mo17_4_PL0018 | Mo17_4_PL0019 | 0.961 |
| Oh43 | Stem | Oh43_5_PL0018 | Oh43_5_PL0019 | 0.971 |
| TIL01 | Stem | TIL01_6_PL0018 | TIL01_6_PL0019 | 0.943 |
| TIL03 | Stem | TIL03_7_PL0018 | NA | ^a^ |
| TIL05 | Stem | TIL05_7_PL0019 | TIL05_8_PL0018 | 0.946 |
| TIL09 | Stem | TIL09_8_PL0019 | TIL09_9_PL0018 | 0.958 |
| TIL10 | Stem | TIL10_10_PL0018 | TIL10_9_PL0019 | 0.947 |
| TIL11 | Stem | TIL11_10_PL0019 | TIL11_11_PL0018 | 0.963 |
| TIL14 | Stem | TIL14_11_PL0019 | TIL14_12_PL0018 | 0.960 |
| TIL15 | Stem | TIL15_12_PL0019 | TIL15_13_PL0018 | 0.939 |
| TIL25 | Stem | TIL25_13_PL0019 | TIL25_14_PL0018 | 0.960 |
| W22 | Stem | W22_14_PL0019 | W22_15_PL0018 | 0.968 |

^a^ Only one biological replicate exists from this genotype.
