# Supplementary material for: The Role of cis Regulatory Evolution in Maize Domestication
Source: PLoS Genet. 2014 Nov 6;10(11):e1004745. doi: 10.1371/journal.pgen.1004745 (PMC4222645; doi:10.1371/journal.pgen.1004745)
Supplement: Table S20 — Comparison of RNAseq expression data with expectations identified in the literature. (DOCX) [file pgen.1004745.s026.docx]

Table S20: RNAseq expression data for putative or documented domestication genes.

|  | **Ear** | | | **Leaf** | | | **Stem** | | |
| --- | --- | --- | --- | --- | --- | --- | --- | --- | --- |
| **GeneID** | **F1 M:T** | **Parent M:T** | **No. F1:M:T** | **F1 M:T** | **Parent M:T** | **No. F1:M:T** | **F1 M:T** | **Parent M:T** | **No. F1:M:T** |
| AC233950.1_FG002 *tb1* | 1041:  687 | 934:  443 | 17:5:8 |  |  |  | 170:  119 | 54:  53 | 17:6:8 |
| GRMZM2G070034 *OsMADS56* | 599:  436 | 312:  202 | 20:6:6 | 1119:  74 | 1413:  59 | 20:6:6 | 231:  23 | 113:  12 | 20:6:6 |
| GRMZM2G074124 *ZmSh1-5.1* | 70:  276 | 15:  205 | 6:4:2 |  |  |  |  |  |  |
| GRMZM2G085873 *ZmSh1.1* | 17:  137 | 5:  220 | 5:3:3 |  |  |  |  |  |  |
| GRMZM2G101511 *tga1* | 102:  114 | 118:  276 | 8:4:4 |  |  |  |  |  |  |
| GRMZM2G180190  *zfl2* | 152:  123 | 256:  63 | 9:5:4 |  |  |  |  |  |  |
| GRMZM2G026223 *zagl1* | 2200:  1469 | 1877:  756 | 17:6:6 | 825:  530 | 289:  246 | 18:6:6 | 434:  304 | 293:  171 | 18:6:6 |
| GRMZM2G370777 *zmm19* | 645:  815 | 157:5  70 | 21:6:9 | 5740:  5856 | 3455:  4107 | 22:6:9 | 13257:  12458 | 5935:  5979 | 22:6:9 |
