# Supplementary material for: The Role of cis Regulatory Evolution in Maize Domestication
Source: PLoS Genet. 2014 Nov 6;10(11):e1004745. doi: 10.1371/journal.pgen.1004745 (PMC4222645; doi:10.1371/journal.pgen.1004745)
Supplement: Table S21 — Increase in size and percent of CCT A, B, and C gene lists when using 5% Storey's FDR for significant binomial and Fisher's exact tests. (DOCX) [file pgen.1004745.s027.docx]

Table S21: Increase in size and percent of CCT A, B, and C gene lists when using 5% Storey’s FDR for significant binomial and Fisher’s exact tests.

| CCT list | Additional Ear (N) | Additional Leaf (N) | Additional Stem (N) | Additional Ear (%) | Additional Leaf (%) | Additional Stem (%) |
| --- | --- | --- | --- | --- | --- | --- |
| A | 1 | 2 | 1 | 2.3% | 8.7% | 3.6% |
| B | 29 | 21 | 33 | 5.4% | 4.7% | 7.6% |
| C | 106 | 116 | 122 | 9.7% | 12.1% | 11.5% |
