# Supplementary material for: The Role of cis Regulatory Evolution in Maize Domestication
Source: PLoS Genet. 2014 Nov 6;10(11):e1004745. doi: 10.1371/journal.pgen.1004745 (PMC4222645; doi:10.1371/journal.pgen.1004745)
Supplement: Text S1 — Supplemental methods and results. (DOCX) [file pgen.1004745.s028.docx]

**Supplemental Methods and Results**

**Weighting and scaling**

Data were scaled and weighted in several ways to account for variable sequencing depth in the course of our analyses. The three statistics that were weighted to account for sequencing depth were: (1) parent read depths for individual maize-teosinte comparisons, (2) overall parent read depth for maize versus teosinte, and (3) percent crosses with consistent *cis* and *trans* expression patterns.

Parent data for individual maize-teosinte comparisons were scaled to the sequencing depth of the parent line with less sequencing reads. This was done by dividing all read depths from both parent inbred lines by the total amount of sequence data collected for each inbred and then multiplying read depths from both inbred lines by the lesser amount of reads collected. For example, if the B73-TIL01 comparison had 20 million B73 reads and a depth at segregating sites of 20 and TIL01 had 10 million reads and depth of 10, B73 read depth was calculated as: 20 depth / 20 million * 10 million and TIL01 read depth was calculated as 10 depth / 10 million * 10 million.

To measure overall maize and teosinte read depth at segregating sites, we combined read depth at segregating sites from the various crosses into a single overall comparison. We did this in different ways for the parent inbred comparisons and the F_1_ hybrid crosses. The independently collected F1 hybrid RNAseq reads were combined by summing gene read depth at segregating SNPs across all hybrids. The parent inbred maize-teosinte comparisons used the same parent inbred RNAseq reads for each “cross” that it was involved in, resulting in non-independence of parent comparisons with shared maize or teosinte parent inbred lines. Consequently we combined maize and teosinte parent expression by calculating the weighted percent maize read depth for each gene. This was done by (A) calculating the percent maize depth (unscaled) per million reads and total depth at each segregating site (B) averaging this percent maize for each gene while weighting each site’s contribution by the total depth of that site and finally (C) calculating final maize and teosinte depth count by multiplying gene maize percent by total depth across all segregating sites and inbred parents and rounding to the nearest whole number.

An important component of the analysis was designation of CCT genes as having consistent *cis* expression in a weighted percentage of crosses. This weighted percentage was calculated as the percentage of assayed F_1_ crosses that favored the maize or teosinte allele weighted by the overall depth of each maize-teosinte comparison. This method for making a weighted percentage was also used in generating lists of genes in the *cis* only (weighted by F_1_ depth) and *trans* only regulatory categories (weighted by parent depth) with consistent expression patterns.

**Drop1 F_1_ analysis**

A drop1 analysis of the F_1_ data was done to assess the average influence of any single data point on overall *cis* effect obtained by summing the F_1_ read depth. This was done by calculating the difference in *cis* effect, log_2_(hybrid ratio), for any single gene caused by dropping a single F_1_ contribution to the read counts and computing an average for each gene. This analysis reveals the vast majority of genes (99.75%) having less than a 0.1 average difference in log_2_(hybrid) value when dropping a single F_1_ contribution to expression ratio (Figure S5). Consequently, we conclude that the average influence of individual F_1_ comparisons is minor and proceeded with calculating overall maize versus teosinte comparisons.

**Alternative FDR cutoff (5%) for significant *cis* and *trans***

While we used an FDR of 0.5% for primary analysis as done by McManus *et al.* [1], we also investigated the increase in size of CCT-ABC gene lists when using a higher FDR (5%) to designate significant binomial and Fisher’s exact tests. The use of 5% FDR is expected to shift genes into the *cis* + *trans* and *cis* x *trans* regulatory categories due to previously non-significant binomial tests (for the F_1_ *cis* effect and parent overall effect) and Fisher’s exact tests (*trans*) becoming significant. This would result in fewer conserved, *cis* only, and *trans* only genes. Of direct impact on this analysis in terms of candidate genes, we expected to only gain genes in the candidate CCT gene lists that show consistent *cis* regulatory effects.

We calculated the increased size in terms of raw number and percent of the CCT gene lists when using a 5% FDR cutoff. As expected, the only difference was adding previously non-CCT genes to the various CCT-ABC gene lists. Table S21 shows the count and percent increase of A, B, and C for each tissue, which was typically quite small with the largest increase in size being for the C list with an increase of approximately 10%. The smallest CCT-A candidate gene lists also saw the smallest increase in size (1, 2, and 1 gene for ear, leaf, and stem respectively). Since the increase in number and percent was relatively minor for the higher confidence CCT-AB gene lists, we used CCT gene lists generated with 0.5% FDR for downstream analyses.

**Calling and filtering of segregating sites with GATK**

GATK [2,3] was used to call variants (both indel and SNP) as well as filter variants by several criteria. The *UnifiedGenotyper* tool and *VariantFiltration* tool were called separately for indel and SNP variants. The important parameter for *UnifiedGenotyper* in both indel and SNP calling was -stand_emit_conf set to 1.0. This sets the minimum confidence for a variant to be output and the setting of 1.0 is very relaxed to allow maximum recovery of tentative variants (segregating sites). The *VariantFiltration* tool used four custom filters that ignored sites with too many/too large fraction of reads with low mapping quality (HARD_TO_VALIDATE), ignored sites if strand bias was too high (Indel/SNPSBFilter), ignored sites if total read depth was less than four or greater than 100 (Indel/SNPDPFilter), and ignored sites with too long homo-polymer runs (SNPHRunFilter). SNPs were further filtered to be ignored if occurring in clusters of three or more in a 10 bp window (-cluster 3 -window 10).

The explicit code used for these calls is as follows for indel variants:

java –jar GenomeAnalysisTK.jar -T UnifiedGenotyper -glm INDEL -R maize_agp_v2.fasta -stand_call_conf 2.0 -stand_emit_conf 1.0 -dcov 100 -A DepthOfCoverage -I alignment.bam -o GATK_indel.vcf

java -jar GenomeAnalysisTK.jar -T VariantFiltration -R maize_agp_v2.fasta -filter "MQ0 >= 4 && ((MQ0 / DP) > 0.1)" -filter "QUAL<30.0" -filter "SB>-1.0" -filter "DP>100 || DP<4" -filterName HARD_TO_VALIDATE -filterName IndelQUALFilter -filterName IndelSBFilter -filterName IndelDPFilter --variant GATK_indel.vcf -o GATK_indel_filtered.vcf

The explicit code used for these calls is as follows for SNP variants:

java –jar GenomeAnalysisTK.jar -T UnifiedGenotyper -glm SNP -R maize_agp_v2.fasta -stand_call_conf 2.0 -stand_emit_conf 1.0 -dcov 100 -A DepthOfCoverage -I alignment.bam -o GATK_snp.vcf

java GenomeAnalysisTK.jar -T VariantFiltration -R maize_agp_v2.fasta --mask:VCF GATK_indel_filtered.vcf --maskName CloseToIndel -filter "MQ0 >= 4 && ((MQ0 / DP) > 0.1)" -filter "SB>=0.10" -filter "HRun>=4" -filter "DP>100 || DP<4" -filterName HARD_TO_VALIDATE -filterName SNPSBFilter -filterName SNPHRunFilter -filterName SNPDPFilter -cluster 3 -window 10 --variant GATK_snp.vcf -o GATK_snp_filtered.vcf

***de novo* assembly**

As an alternative to pseudo-transcriptomes, we briefly explored the possibility of using *de novo* transcriptomes assembled for each parental line from RNAseq data. If such accurate parental transcriptomes were available, allele-specific expression levels could be estimated for matched transcripts (paired by clustering or anchoring to the B73 annotated transcriptome) in terms of RPKM values obtained using any standard RNAseq analysis pipeline. Preliminary tests of this strategy were performed using Trinity [4] as the transcriptome assembler. These tests showed that the assembly was unable to properly represent gene duplication events, often producing chimeras of two paralogs, different in the two lines being compared. This effect is probably exacerbated by the fact that only single-end reads were used in our assembly. Since this problem significantly complicates a meaningful comparison of allele expression, we decided not to pursue this avenue in the present work.

**Other domestication candidate genes**

We examined six genes identified in the literature as potential maize domestication genes and two known domestication genes (*tb1* and *tga1*) and compared the RNAseq expression data to expectations for these genes (Table S20). The RNAseq data for *tb1* and *tga1* match published qRT-PCR expression data. Three of the six other genes had predicted expression biases based on gene function and all three fit these expectations. The three remaining putative domestication genes (*OsMADS56*, *zagl1*, and *zfl2*) all show a maize-teosinte expression difference, however neither phenotype nor gene function provide a prediction as to whether the maize or teosinte allele should be expressed more highly. Here we review these eight genes.

The evolution of maize plant and ear architecture is controlled in part by *teosinte branched1* (*tb1*) [5]. In domesticated maize, high expression of *tb1* leads to arrest of bud outgrowth and a more apically dominant plant, as well as changes in ear morphology. In the ear RNAseq data, *tb1* was classified as *cis* + *trans* with slightly less than a two-fold increase in maize. This is consistent with expectations from the literature [5]. In our collection of teosinte inbreds, two lines (TIL03 and TIL09) are known to carry the maize haplotype at *tb1*. Thus, F_1_s with these teosinte inbreds should not show a maize-teosinte expression difference and they do not. F_1_s involving TIL03 and TIL09 having a sum maize:teosinte expression ratio of 239:261 (approximately equal expression) while other F_1_s sum to 802:426 (nearly 2-fold higher maize expression). This precisely matches published expectation and it is an impressive example of the power of our experiment to accurately quantify allele specific expression for genes and alleles of interest.

The maize domestication gene *teosinte glume architecture* (*tga1*) controls the development of the fruitcase that covers the kernels in teosinte which is lacking in maize [6]. Published qRT-PCR data show equal expression of the maize and teosinte alleles, and an amino acid substitution was implicated as the causative polymorphism that was the target of selection during domestication. Consistent with this information, the RNAseq data show equal allele-specific expression in the F_1_s although higher teosinte expression in the parent data because the fruitcase structure develops in the teosinte ear but not in the maize ear. Again, the data match published information.

Pod corn, a phenotype with the kernels covered by a glume, has been shown to be caused by increased expression of *zea mays MADS19* (*zmm19*) in the developing female inflorescence [7]. In the RNAseq data, the teosinte allele is expressed 3.63X higher than the maize allele and the gene is classified as *cis* + *trans*. This higher expression of the teosinte allele agrees with the prediction that higher expression of *zmm19* contributes to glume growth and encased kernels (a teosinte phenotype). Interestingly, this expression difference is specific to the ear and expression is very close to 1:1 in the leaf and stem, suggesting that there may be tissue specific regulatory elements in its promoter.

Two YABBY transcription factors (*ZmSh1-1* and *ZmSh1-5.1*) have been identified as candidates for genes controlling ear shattering in teosinte and loss of shattering in maize [8]. Based on analysis of these genes in *Sorghum*, the prediction is that the maize (non-shattering) allele should be expressed lower than the teosinte allele [8]. Although there is RNAseq data for only a few F1s, the data meet the prediction with lower expression of the maize allele. Furthermore, we have experimentally confirmed this result by qRT-PCR (Doebley lab, unpublished).

The *Arabidopsis* ortholog of *zea agamous like1* (*zagl1*), *SOC1*, was identified as a selection candidate gene [9]. In Arabidopsis, this gene (*SOC1*) functions in the flowering time pathway [10]. QTL fine-mapping data from the Doebley Lab (unpublished) show that the maize allele confers more rows of kernels in the ear. The RNAseq data shows elevation of maize expression in the ear, however we have no prior hypothesis to predict that higher expression is expected based in phenotype or gene function.

The maize homolog of *OsMADS56* (GRMZM2G070034) was identified as a putative target of selection and shown with expression microarray analysis to have higher expression in maize [11]. The RNAseq data show much higher expression for the maize allele in all three experimental tissues and the gene is classified as having largely *cis* effects (average *cis* effect of 2.57 and average *trans* of 0.25). In rice, over-expression of *OsMADS56* leads to delayed flowering in long day conditions [12], however flowering time is not a domestication trait. Many flowering time genes also affect inflorescence structure, and this gene was potentially under selection for an effect on inflorescence structure. We have no prior hypothesis to predict that higher expression in maize is expected based on phenotype or gene function.

One of the maize homologs of the *Antirrhinum* *floricaula* and *Arabidopsis* *leafy* gene called *zfl2* has been designated as a putative maize domestication gene based on location near a QTL for kernel row number and a genetic complementation test [13]. *zfl2* was also tagged as a selection candidate gene [11]. In *Antirrhinum* and *Arabidopsis*, the gene functions in the flowering time pathway [14]. Although flowering time is not a domestication trait, *zfl2* influences kernel row number and inflorescence structure in maize [13] and these are domestication traits. RNAseq data show slightly higher allele specific expression of the maize allele. There are no published qRT-PCR data to which the RNAseq data can be compared.

Taken as a group, these eight genes fit one or more of the following criteria: (1) show a maize-teosinte expression bias that is consistent with published qRT-PCR data, (2) show a maize-teosinte expression bias that is consistent with expectations based on gene function, and (3) show a maize-teosinte expression bias that supports the gene’s role as a domestication candidate gene, although the directionality of the bias was not predicted.

**Supplemental Text References:**

1. McManus CJ, Coolon JD, Duff MO, Eipper-Mains J, Graveley BR, et al. (2010) Regulatory divergence in Drosophila revealed by mRNA-seq. Genome Res 20: 816–825. doi:10.1101/gr.102491.109.

2. McKenna A, Hanna M, Banks E, Sivachenko A, Cibulskis K, et al. (2010) The Genome Analysis Toolkit: a MapReduce framework for analyzing next-generation DNA sequencing data. Genome Res 20: 1297–1303. doi:10.1101/gr.107524.110.

3. DePristo MA, Banks E, Poplin R, Garimella K V, Maguire JR, et al. (2011) A framework for variation discovery and genotyping using next-generation DNA sequencing data. Nat Genet 43: 491–498. doi:10.1038/ng.806.

4. Grabherr MG, Haas BJ, Yassour M, Levin JZ, Thompson DA, et al. (2011) Full-length transcriptome assembly from RNA-Seq data without a reference genome. Nat Biotechnol 29: 644–652. doi:10.1038/nbt.1883.

5. Studer A, Zhao Q, Ross-Ibarra J, Doebley J (2011) Identification of a functional transposon insertion in the maize domestication gene tb1. Nat Genet 43: 1160–1163. doi:10.1038/ng.942.

6. Wang H, Nussbaum-Wagler T, Li B, Zhao Q, Vigouroux Y, et al. (2005) The origin of the naked grains of maize. Nature 436: 714–719. doi:10.1038/nature03863.

7. Wingen LU, Münster T, Faigl W, Deleu W, Sommer H, et al. (2012) Molecular genetic basis of pod corn (Tunicate maize). Proc Natl Acad Sci 109: 7115–7120. doi:10.1073/pnas.1111670109.

8. Lin Z, Li X, Shannon LM, Yeh C-T, Wang ML, et al. (2012) Parallel domestication of the Shattering1 genes in cereals. Nat Genet 44: 720–724. doi:10.1038/ng.2281.

9. Vigouroux Y, McMullen M, Hittinger CT, Houchins K, Schulz L, et al. (2002) Identifying genes of agronomic importance in maize by screening microsatellites for evidence of selection during domestication. Proc Natl Acad Sci U S A 99: 9650–9655. doi:10.1073/pnas.112324299.

10. Lee H (2000) The AGAMOUS-LIKE 20 MADS domain protein integrates floral inductive pathways in Arabidopsis. Genes Dev 14: 2366–2376. doi:10.1101/gad.813600.

11. Hufford MB, Xu X, van Heerwaarden J, Pyhäjärvi T, Chia J-M, et al. (2012) Comparative population genomics of maize domestication and improvement. Nat Genet 44: 808–811. doi:10.1038/ng.2309.

12. Ryu C-H, Lee S, Cho L-H, Kim SL, Lee Y-S, et al. (2009) OsMADS50 and OsMADS56 function antagonistically in regulating long day (LD)-dependent flowering in rice. Plant Cell Environ 32: 1412–1427. doi:10.1111/j.1365-3040.2009.02008.x.

13. Bomblies K, Doebley JF (2006) Pleiotropic effects of the duplicate maize FLORICAULA/LEAFY genes zfl1 and zfl2 on traits under selection during maize domestication. Genetics 172: 519–531. doi:10.1534/genetics.105.048595.

14. Weigel D, Alvarez J, Smyth DR, Yanofsky MF, Meyerowitz EM (1992) LEAFY controls floral meristem identity in Arabidopsis. Cell 69: 843–859.
